# Estimating Dengue Transmission Intensity from Case-Notification Data from Multiple Countries

**DOI:** 10.1371/journal.pntd.0004833

**Published:** 2016-07-11

**Authors:** Natsuko Imai, Ilaria Dorigatti, Simon Cauchemez, Neil M. Ferguson

**Affiliations:** 1 MRC Centre for Outbreak Analysis and Modelling, Department of Infectious Disease Epidemiology, Imperial College London, London, United Kingdom; 2 Mathematical Modelling of Infectious Diseases Unit, Institut Pasteur, Paris, France; Institute for Disease Modeling, UNITED STATES

## Abstract

**Background:**

Despite being the most widely distributed mosquito-borne viral infection, estimates of dengue transmission intensity and associated burden remain ambiguous. With advances in the development of novel control measures, obtaining robust estimates of average dengue transmission intensity is key for assessing the burden of disease and the likely impact of interventions.

**Methodology/Principle Findings:**

We estimated the force of infection (*λ*) and corresponding basic reproduction numbers (R_0_) by fitting catalytic models to age-stratified incidence data identified from the literature. We compared estimates derived from incidence and seroprevalence data and assessed the level of under-reporting of dengue disease. In addition, we estimated the relative contribution of primary to quaternary infections to the observed burden of dengue disease incidence. The majority of *R*_0_ estimates ranged from one to five and the force of infection estimates from incidence data were consistent with those previously estimated from seroprevalence data. The baseline reporting rate (or the probability of detecting a secondary infection) was generally low (<25%) and varied within and between countries.

**Conclusions/Significance:**

As expected, estimates varied widely across and within countries, highlighting the spatio-temporally heterogeneous nature of dengue transmission. Although seroprevalence data provide the maximum information, the incidence models presented in this paper provide a method for estimating dengue transmission intensity from age-stratified incidence data, which will be an important consideration in areas where seroprevalence data are not available.

## Author Summary

With 40% of the world’s population at risk of infection, dengue imposes a significant public health burden. Yet estimates of baseline transmission intensity are still sparse, making it difficult to implement efficient control programs. The authors used incidence data, which are abundant compared to seroprevalence data, to estimate dengue transmission intensity in 13 countries. Estimates derived from incidence data were comparable to those from seroprevalence data, an important conclusion for areas where seroprevalence data are not available. Additionally, the estimated baseline reporting rates and the contribution of primary to tertiary/quaternary infections to observed disease in each country will help to highlight potential weaknesses in the country or region’s surveillance system.

## Introduction

Dengue is the most widely distributed mosquito-borne viral infection, but assessment of its geographic variation in transmission remains challenging. Analysis based on mapping the probability of occurrence of dengue estimated that dengue causes 390 million annual infections worldwide [1]. However, these estimates relied on assuming a direct linear correlation between the probability of occurrence and incidence, rather than estimating transmission intensity as quantified by the force of infection or reproduction number. Here we develop methods to estimate transmission intensity from routine, age-stratified surveillance data on suspected dengue case incidence.

All four serotypes of dengue virus (DENV-1, 2, 3, and 4) can cause dengue fever with the risk of severe dengue increasing with subsequent heterologous infections. Once infected, individuals develop long-lived protective homotypic immunity and short-lived heterotypic immunity [2,3]. Once antibody levels wane below the threshold required to provide protection, antibody-dependent enhancement (ADE) becomes a risk, leading to secondary heterologous infection having an increased risk of causing clinically apparent disease [4,5]. Hence, while the majority of primary dengue infections are asymptomatic [6,7], secondary heterologous infection has been identified as a major risk factor for symptomatic and severe dengue [8–10]. Therefore the majority of cases seen in hospitals [11] or reported via surveillance systems [12] tend to be secondary infections [7].

In previous work, we estimated dengue transmission intensity from age-stratified seroprevalence data but highlighted the relative paucity of seroprevalence data compared with routine surveillance data on the incidence of suspected dengue [13]. This reflects dengue fever, dengue haemorrhagic fever (DHF), and dengue shock syndrome (DSS) being notifiable diseases in most countries [14–18]. Indeed, in many countries, incidence reports are the only type of data available. However the clinical diagnostic criteria vary and different countries have their own reporting standards [19]. The World Health Organisation (WHO) collates surveillance data from dengue affected countries via its DengueNet system, but the data are not always updated regularly and there can be inconsistencies with other sources (e.g. WHO regional offices or countries) of national and subnational data [19].

The lack of systematic data on dengue incidence, the lack of standardised reporting procedures or diagnostic criteria, and the lack of integration between private and public sectors makes accurate estimation of the true dengue burden difficult [20]. Previous studies have attempted to estimate the burden of dengue and associated economic costs in South East Asia and South America by calculating expansion factors from systematic literature reviews, collation of existing data, and population-based cohorts [20–24]. However, the lack of standardisation also affects the validity of expansion factors (calculated by dividing the cumulative incidence of dengue cohort studies by that from passive data at national and local levels) as estimates of underreporting. Due to the wide spectrum of clinical manifestations and the lack of routine laboratory testing, dengue is globally underreported and analyses of officially reported dengue numbers need to take this into account [25].

While reported incidence levels cannot be relied upon to directly quantify disease burden, the age distribution of dengue cases provides more reliable information on dengue transmission intensity. Here we propose an approach for estimating average transmission intensity—as quantified by the force of infection (*λ*) or basic reproduction number (*R*_0_)–from age-stratified incidence data. We compare estimates derived from seroprevalence and incidence data and assess the level of under-reporting of dengue disease. In addition, we estimate the relative contribution of primary to quaternary infections to the observed burden of dengue disease incidence.

## Methods

### Literature search

Web of Knowledge and PubMed were searched for age-stratified incidence data since 1980 as we were interested in contemporary dengue transmission and wanted to be consistent with our previous study where we collated age-stratified seroprevalence data [13]. Search terms used were ‘dengue’ and ‘age’ and (‘incidence’ or ‘cases’ or ‘notifications’ or ‘notified cases’) with inclusion criteria mapped to subject headings. Additional web-based searches were performed to augment the primary literature search. Data were extracted from published datasets where authors reported age-stratified incidence data with corresponding population age-structure data.

### Estimating the force of infection and reporting rates

We considered a population stratified into *M* age groups and denote *a*_*j*_ and *a*_*j+1*_ the lower and upper age bounds respectively of age group *j* (*j = 0*,*…*, *M-1*). Our model assumes perfect homotypic protection following infection with any serotype. Thus, an individual can experience a maximum of four dengue infections in their life (corresponding to the four dengue serotypes). Ideally, we would allow forces of infection to vary by serotype (DENV-1 to DENV-4). However as serotype-specific data were not available, we assumed circulating serotypes were equally transmissible, i.e. had the same force of infection, *λ*, which did not vary over time. The incidence of primary infections (*I*_1_) for any one serotype for people in an age group *j* was calculated as the integral of the probability of being seronegative to all four strains at age *a* multiplied by four times the constant serotype-specific infection hazard, *λ* (since primary infection can occur with any of the four serotypes). Age *a* spans the range [*a*_*j*_,*a*_*j*+1_], as described by the bounds of integrations (Eq 1).

$$\begin{array}{ll} I_{1}\left(j\right) & =\int_{a_{j}}^{a_{j+1}}4\lambda {\left(e^{-\lambda a}\right)}^{4}da\\ & =\int_{a_{j}}^{a_{j+1}}4\lambda e^{-4\lambda a}da=\left(e^{-4\lambda a_{j}}-e^{-4\lambda a_{j+1}}\right) \end{array}$$ (1)

The incidence of secondary, tertiary, and quaternary infections in age group *j* (*I*_2_(*j*), *I*_3_(*j*), and *I*_4_(*j*) respectively) are calculated in a similar fashion. If fewer than four serotypes have circulated in an area, then the number of infections an individual can have changes accordingly. Full details are given in the Supporting Information (S1 Text).

The average observed annual disease incidence rate per person in age group *j* is then given by the weighted sum of the primary to quaternary infection rates (Eq 2):

$$D\left(j\right)=\frac{\rho }{w\left(j\right)}\left\{I_{2}\left(j\right)+\gamma_{1}\left(I_{1}\left(j\right)+\gamma_{3}\left(I_{3}\left(j\right)+I_{4}\left(j\right)\right)\right)+B\right\}$$ (2)

where *w*(*j*) = *a*_*j*+1_ − *a*_*j*_ is the width of age group *j*, *ρ* is the probability that a secondary infection results in a detected dengue case (reporting rate), *γ*_1_ is the probability that a primary infection is detected relative to a secondary infection, and *γ*_3_ is the probability that a tertiary or quaternary infection is detected relative to a primary infection. Here *B* is a baseline risk of disease used to represent any non-dengue related illnesses that are misdiagnosed as dengue, and was only estimated when fitting suspected dengue incidence data where laboratory confirmation was lacking.

We assumed that secondary infections were more likely to be symptomatic than primary infections [7,26] and that post-secondary infections were even less likely to be symptomatic than primary infections, i.e. *ρ>γ*_1_>*γ*_3_. Single values of *γ*_1_ and *γ*_3_ were estimated per country. For datasets that reported DHF only, we assumed that DHF cases only arose from secondary infections and set *γ*_1_ and *γ*_3_ to zero [27,28]. Where fewer than four serotypes were in circulation, we adjusted our calculation of the expected incidence accordingly—full details are given in the S1 Text. Where data on the age distribution of the population was not provided in the source publications, the population age-structure closest to the survey population was used (taken from census data or from United Nations estimates) [29]. For the first model variant examined (model 1), we assumed a single baseline reporting rate (*ρ*) across all age groups. We also explored whether baseline reporting rates might differ with age (model 2) by estimating different reporting rates in children (*ρ*_*young*_) and adults (*ρ*_*old*_), also fitting the age threshold (*a*_*threshold*_) defining the boundary between these groups (*ρ*_*young*_) for age *a < a*_*threshold*_, otherwise *ρ*_*old*_).

Where incidence data were available for multiple years, we fitted models 1 and 2 to individual years (model variants 1A and 2A). We also examined fitting to the cumulative incidence across the observation period, as this gives a better estimate of the long-term average distribution of incidence across age groups (models 1B and 2B). When fitting to the cumulative incidence we calculated the expected disease incidence by multiplying the annual expected disease incidence by the number of years in the study. Overall, for models 1A and 1B, we estimated up to 5 parameters (*λ*, *ρ*, *γ*_1_, *γ*_3_ and *B*), while for models 2A and 2B we estimated up 7 parameters (*λ*, *ρ*_*young*_, *ρ*_*old*_, *a*_*threshold*_, *γ*_1_, *γ*_3_ and *B*). All models were fitted to the data using a Metropolis-Hastings Markov Chain Monte Carlo (MH MCMC) algorithm using a Dirichlet-multinomial log-likelihood with uniform priors in version 3.1.0 of the R statistical language [30]. Full details are given in the S1 Text.

### Calculating the basic reproduction number (*R*_0_)

We assumed dengue transmission was at endemic equilibrium and that the force of infection (*λ*) was constant in time. Since we did not have serotype-specific data, we additionally assumed that all serotypes in circulation were equally abundant and equally transmissible, i.e. had the same force of infection and basic reproduction number, and that there were no interactions between serotypes. We estimated a strain-specific basic reproduction number (*R*_0_) from the single force of infection (*λ*) estimated under two different assumptions about the number of infections required to acquire complete immunity. Under assumption one, complete protection is acquired upon quaternary infection. Under assumption two, complete protection is reached after secondary infection (or if tertiary and quaternary infections occur, they are not infectious). These assumptions match that of our previous work estimating the force of infection from serological data and allowed us to compare the *R*_0_ estimates obtained from both types of data [13]. Full details are given in the S1 Text.

### Comparing force of infection estimates by data type

We used weighted regression to assess how comparable force of infection estimates obtained from cumulative incidence data were with those derived from seroprevalence data described previously [13] and from four additional seroprevalence datasets (see Table S1 in S1 Text). Location- and time-matched incidence and serology data were not available, so we matched datasets by country, region, and survey year. Since seroprevalence data represent all past infections, we compared force of infection estimates with those obtained from cumulative incidence data rather than yearly incidence data where possible (see Table S2 in S1 Text for full details on pairings). We used the weighted regression method described by Ripley and Thompson [31] which explicitly accounts for measurement errors in both force of infection estimates from seroprevalence data (y-axis) and incidence data (x-axis) to estimate the maximum likelihood estimate (MLE) line. This was implemented using the *deming* package in R [32]. Full details are given in the S1 Text.

## Results

**Fig 1:**
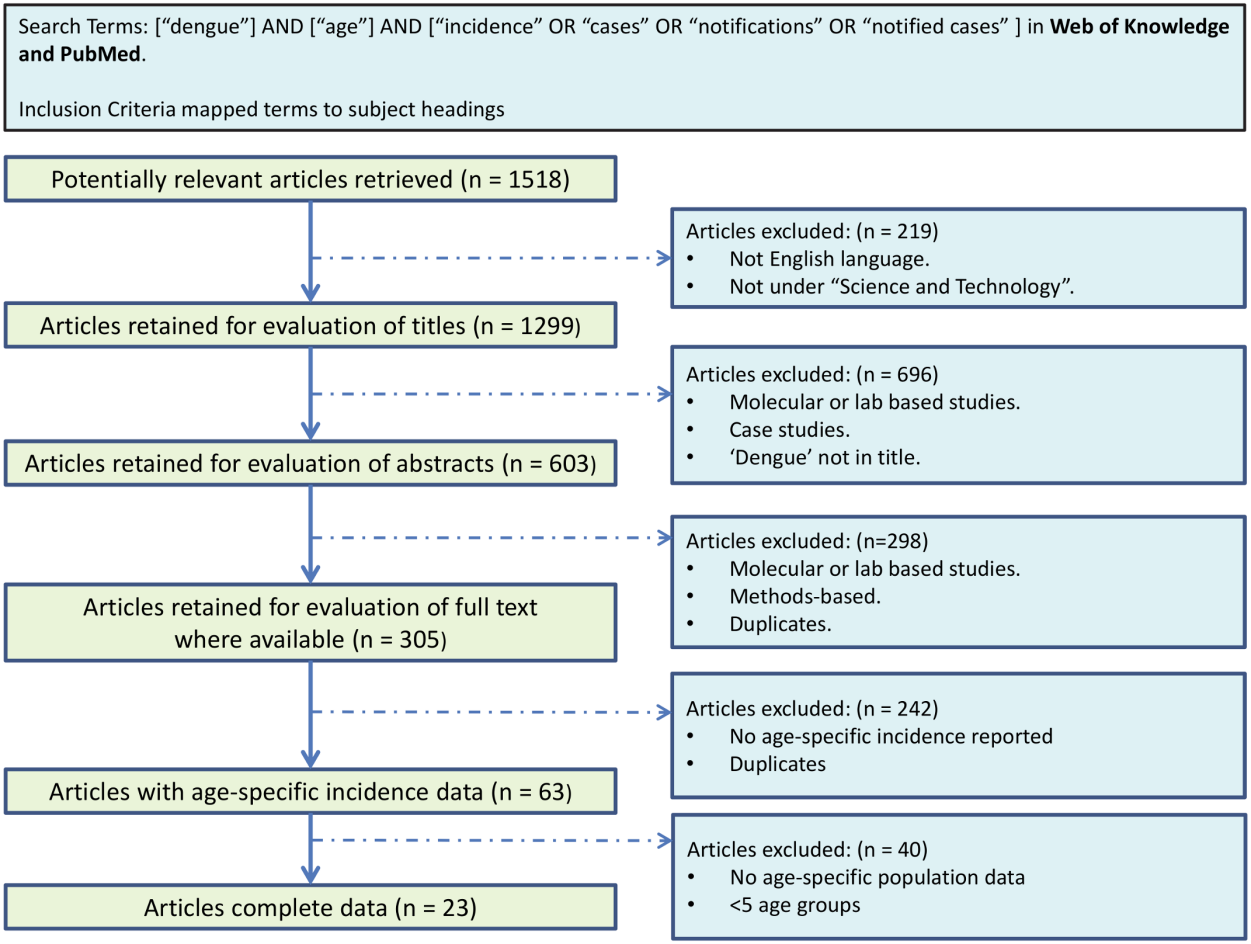
Flowchart describing the literature search process for age-stratified incidence data.

We identified 23 papers reporting incidence data. Fig 1 describes the search process and Table 1 summarises the studies identified. Seven papers reported age-stratified incidence data from multiple years, one paper reported data where the number of serotypes in circulation had changed over the survey years, six papers reported cumulative age-stratified incidence data, eight papers reported age-stratified incidence data from a single year, and two papers reported age-stratified incidence data from multiple countries.

**Table 1:** Summary of cross-sectional incidence datasets identified and associated demographics.

Country	Survey Year	Region	Diagnosis	# serotypes in circulation	DF/DHF/DSS	Age range sampled	Estimated denominator population of study (3sf)^	Population size of study region	Urban/Rural	Ref
Brazil	1995–2001	Pernambuco State	Lab confirmed	2	All cases	0–80+	8360000	8.5M	Urban/Rural	[35]
	2002–2006	Pernambuco State	Lab confirmed	3	All cases	0–80+	8360000	8.5M	Urban/Rural	[35]
	2000–2009	Vitoria	Lab confirmed	3	All cases	0–80+	292000	0.28M–0.32M	Urban/Rural	[36]
	2001–2006	Amazon	Clinical	4	All cases	0–70	3480000	23.6M	Rural/Urban	[37]
Cambodia	2006–2008	Kampong Chan Province	Lab confirmed*	4	All cases	0–20	805000	90000	Urban/Rural	[38]
	2006–2007	Kampong Chan	Lab confirmed	4	All cases	0–14	14500	90000	Urban/Rural	[39]
China	1978–1988	Guangzhou	Clinical	4	All cases	0–71+	69700000	11.64M	Urban	[33]
	1989–1999	Guangzhou	Clinical	4	All cases	0–71+	69000000	11.64M	Urban	[33]
	2000–2009	Guangzhou	Clinical	4	All cases	0–71+	39500000	11.64M	Urban	[33]
	2005–2011	Guangdong	Clinical	4	All cases	0–80+	88900000	104.3M	Urban	[14]
Laos	2000–2006	National	Clinical/Lab	4	All cases	0–15+	4980000	5.4M	Urban/Rural	[40]
	2010	Savannakhet Province	Clinical	4	All cases	0–40+	4880000	0.83M	Urban	[41]
	2010	National	Clinical	4	All cases	0–40+	6390000	6.5M	Urban/Rural	[42]
Nicaragua	1999–2001	Leon	Lab confirmed	3	All cases	0–55	360000	0.39M	Urban	[43]
Philippines	1998–2005	National	Clinical/Lab	4	All cases	0–15+	71700000	77.7M	Urban/Rural	[40]
Puerto Rico	2006	Patillas	Lab confirmed	4	All cases	0–40+	16700	20200	Urban	[44]
	2007	National	Lab confirmed	4	All cases	0–70+	3820000	3.8M	Urban/Rural	[45]
	2010	National	Lab confirmed	4	All cases	0–70+	3720000	3.7M	Urban/Rural	[12]
	1994	National	Lab confirmed	3	All cases	0–75+	3530000	3.5M	Urban/Rural	[46]
	1995–1997	National	Lab confirmed	3	All cases	0–75+	3530000	3.5M	Urban/Rural	[46]
Singapore	1999–2005	National	Clinical/Lab	4	All cases	0–15+	2620000	4M	Urban	[40]
	2005	National	Lab confirmed	4	DF/DHF	0–80	3450000	4.3M	Urban	[47]
	2005	National	Lab confirmed	4	All cases	0–55+	4270000	4.3M	Urban	[48]
	2007	National	Lab confirmed	4	All cases	1–55+	4590000	4.6M	Urban	[48]
Sri Lanka	1997	National	Clinical	4	DF/DHF	0–65	17300000	17.3M	Urban/Rural	[49]
	1996–2005	National	Clinical	4	All cases	0–15+	17700000	17.3M	Urban/Rural	[40]
Taiwan	2003–2009	Kaohsiung City	Lab confirmed	4	All cases	0–74+	10600000	1.5M	Urban	[50]
Thailand	2000–2010	National	Clinical	4	All cases	0–65+	797000	66.4M	Urban/Rural	[51]
	2006–2007	Ratchaburi	Lab confirmed	4	All cases	0–14	6380	38208	Urban	[39]
	2000	Bangkok	Lab confirmed	4	DHF	0–65	5050	6355144	Urban	[34]
	2000	Ratchaburi	Lab confirmed	4	DHF	0–65	1370	791217	Urban	[34]
	2010	Rayong	Lab confirmed	4	All cases	0–72	1060	616916	Urban	[34]
Vietnam	1998–2009	Hanoi	Lab confirmed	4	All cases	0–80	6350000	6.5M	Urban	[52]
Yemen	2010	Hadramout	Lab confirmed	3	All cases	0–55+	797000	0.7M	Urban/Rural	[53]

*with active surveillance.

^Calculated from the population size and reported incidence if survey numbers were not given in the source publication.

The identified studies provided a total of 34 datasets from 13 countries. The years included ranged from 1978 to 2011. The dataset reporting incidence data from 1978 was included since data were presented for the eleven-year time period of 1978–1988 [33]. Of the 23 papers reporting incidence data, ten reported dengue incidence at the national level and only two studies reported cases detected via active as well as passive surveillance. Three additional surveys were obtained from the Ministry of Health in Thailand that reported age-specific incidence from Bangkok (2000), Ratchaburi (2000), and Rayong (2010) [34].

**Fig 2:**
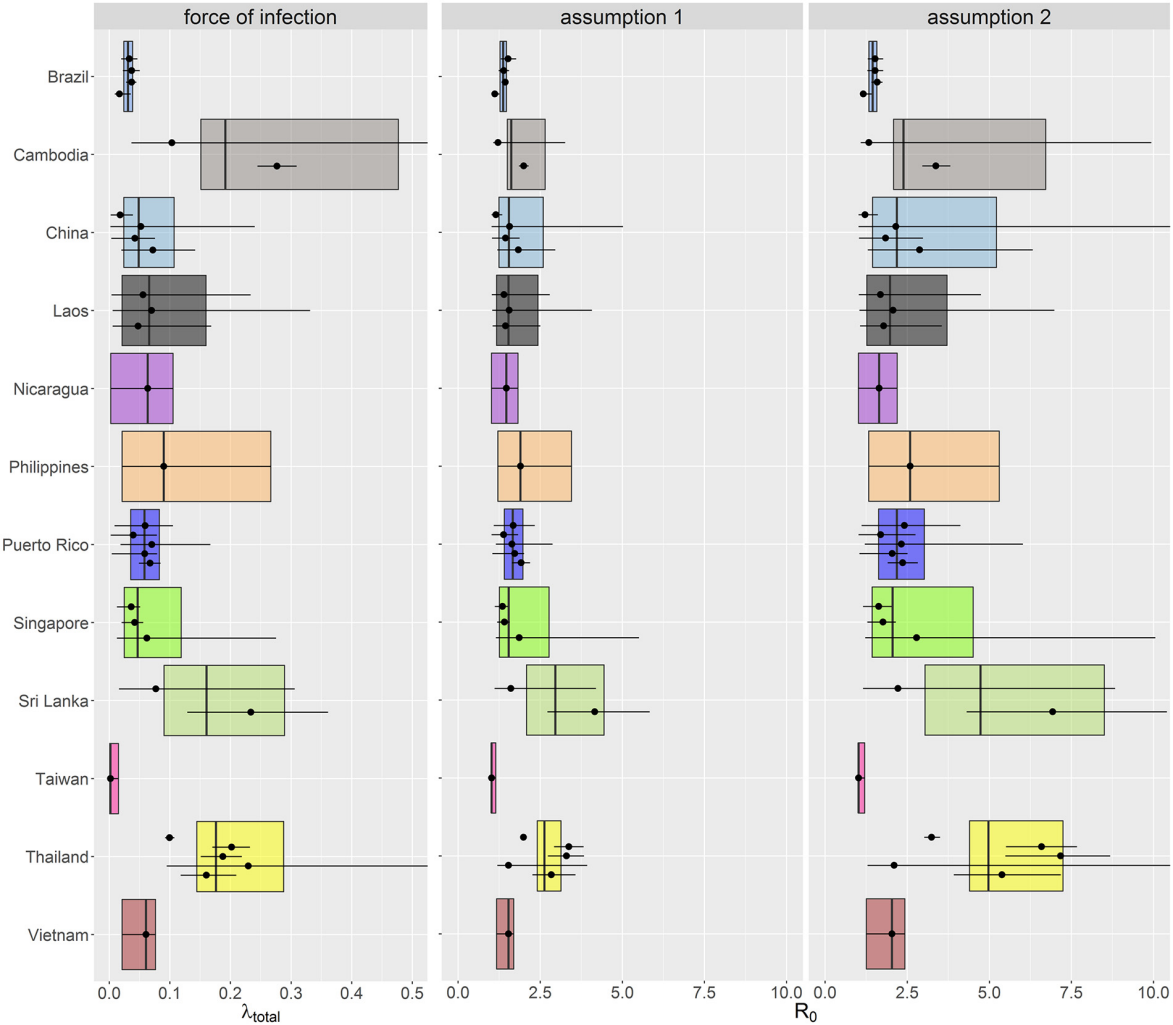
Total force of infection and corresponding R_0_ estimates from the model fitted to the incidence data grouped by country. Each dot represents the posterior median estimate and the error bars show the 95% CrI for each dataset. The box represents the country-specific central estimate calculated by taking the mean values of the MCMC output for each country (the line and limits of the box represents the posterior median and the 95% CrI respectively). R_0_ assumption one: complete protection acquired upon quaternary infection, assumption two: complete protection reached after secondary infection.

As expected, force of infection estimates varied widely between countries, with less variation seen within countries. Fig 2 shows the distribution of the total force of infection (*λ*_*total*_) grouped by country (calculated by multiplying the serotype-specific force of infection by the number of serotypes in circulation). Individual estimates are given in the S1 Text.

**Fig 3:**
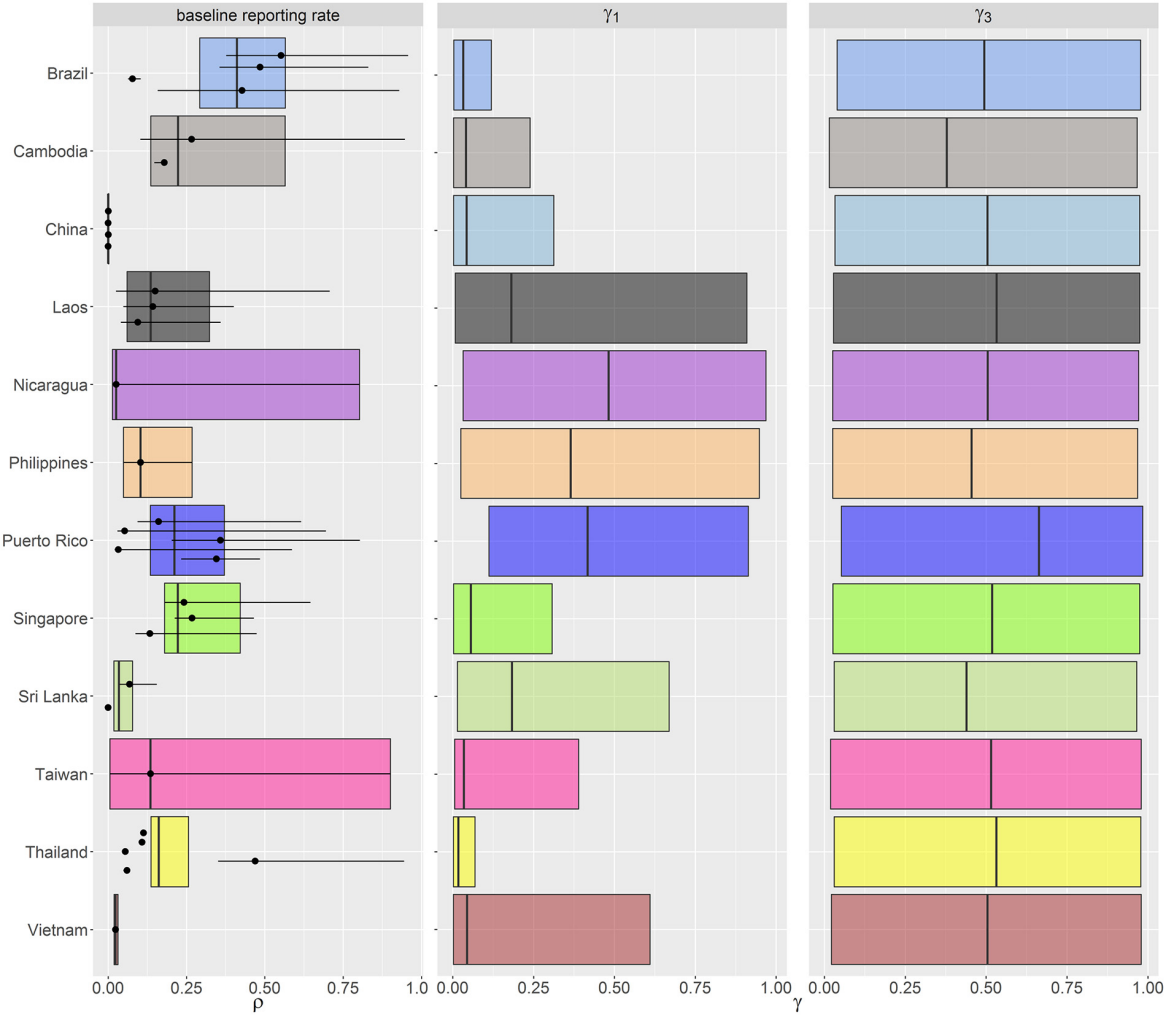
Summary of estimated reporting rates showing the baseline reporting rate or probability of detecting a secondary infection (ρ), the probability of detecting a primary infection (γ1) relative to a secondary infection, and the probability of detecting a tertiary/quaternary infection (γ3) relative to a primary infection. Each point represents the posterior median estimate and the error bars show the 95% CrI for each dataset. The box represents the country-specific central estimate calculated by taking the mean values of the MCMC output for each country (the line and limits of the box represents the posterior median and the 95% CrI respectively). A single overall value of γ1 and γ3 were estimated per country.

Estimates of *R*_0_ varied according to the assumptions made regarding host immunity. Assuming only primary and secondary infections are infectious (assumption two) gave up to two-fold higher estimates of *R*_0_ than when assuming tertiary and quaternary infections are also infectious (Fig 2). This is consistent with our previous results analysing seroprevalence data [13]. Some force of infection estimates in Cambodia were very high, perhaps as a result of the active surveillance undertaken as part of the study by Vong *et al*. [38] (for all parameter estimates see S1 Text). The baseline reporting rate (*ρ*), defined as the probability of detecting a secondary infection, was less than 15% when averaged across all studies (Fig 3). The median probability of detecting a primary infection relative to that of detecting a secondary infection (*γ*_1_) was less than 25% for the majority of datasets. However, the credible intervals for some *γ*_1_ estimates were wide. The data proved uninformative about the contribution of post-secondary infections to disease incidence, as our estimates of *γ*_3_ (Fig 3) reflected the prior distribution assumed for that parameter (uniform from 0 to 1).

The baseline reporting rates (*ρ*) varied substantially by country (Fig 3), likely reflecting differences in healthcare seeking behaviour and surveillance. Generally, estimated reporting rates in the Americas were higher than in South East Asia, with Singapore having the highest rate within SE Asia. Reporting rates also varied within each country depending on survey year or survey region, which may reflect differences in local healthcare systems or changes in public awareness after epidemics.

**Fig 4:**
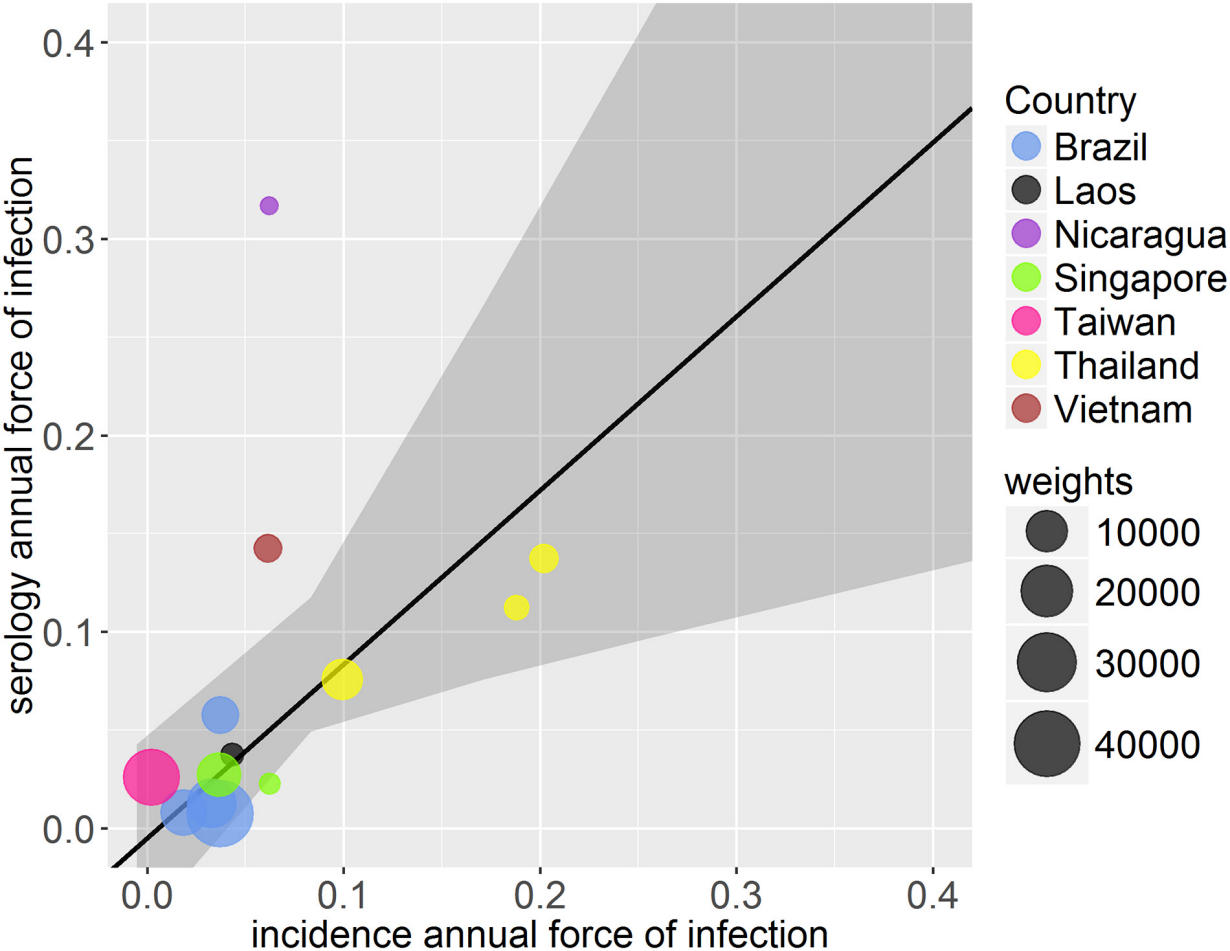
Comparison of weighted deming regression of force of infection estimates by country from cumulative incidence data and seroprevalence data. Each point is weighted depending on the error in both serology and incidence estimates, represented by the size of circles (larger circles indicating greater weight, i.e. smaller error).

**Fig 5:**
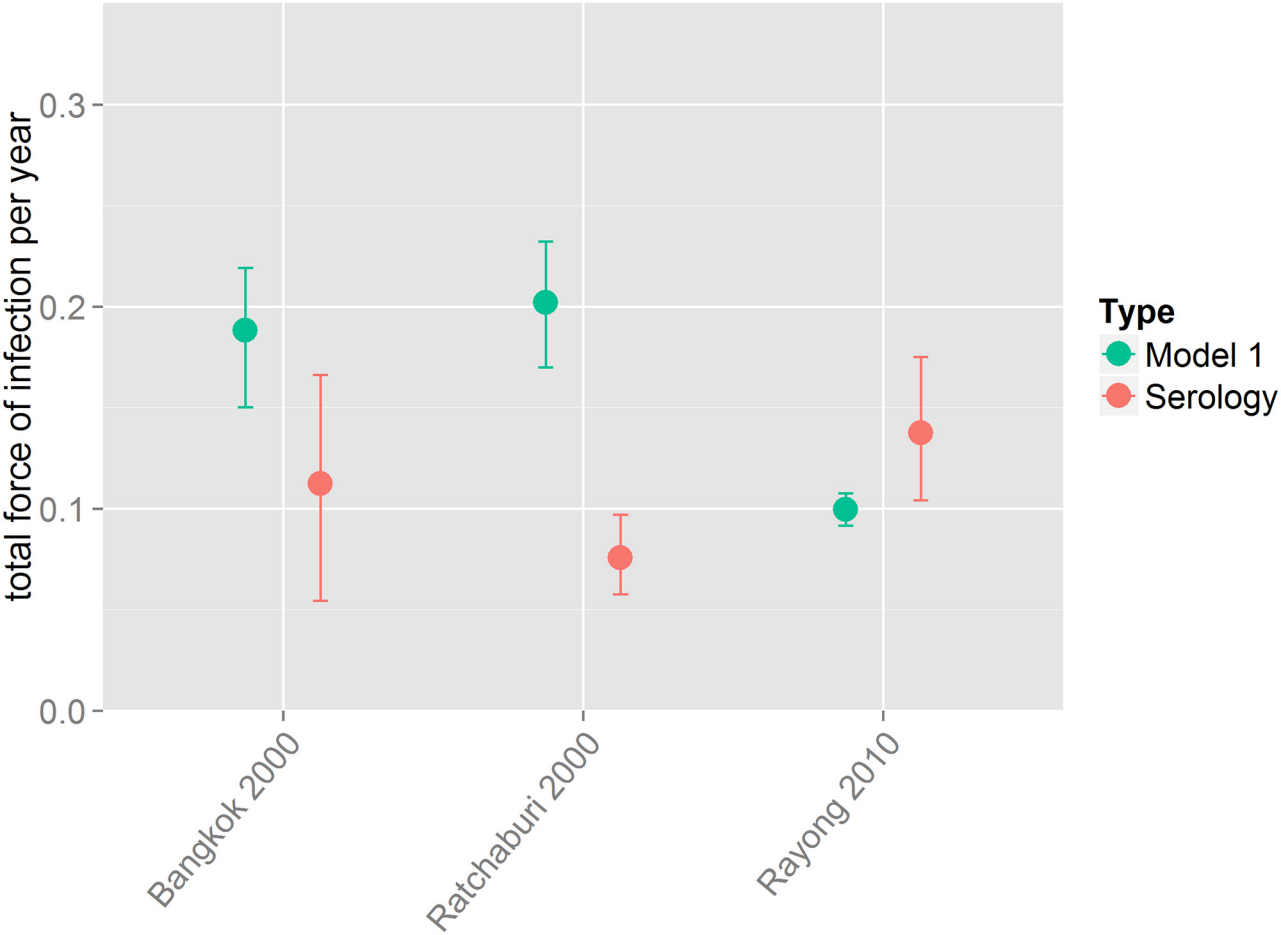
Posterior median estimates of the total force of infection from the model fitted to incidence data (model 1) and model A (as described in [13]) to age-stratified seroprevalence data (serology) from Thailand where incidence and serology data were available from the same year and location.

We used weighted regression to compare the force of infection estimates obtained from age-stratified seroprevalence data to cumulative incidence data. Estimates obtained from the model fitted to the cumulative incidence data were largely comparable to force of infection estimates from seroprevalence data (Fig 4). The majority of the total force of infection (*λ*_*total*_) estimates from incidence data (calculated by multiplying the serotype-specific force of infection by the number of serotypes in circulation) were comparable to those obtained from seroprevalence data when *λ*_*total*_ was smaller than ~0.1 with greater uncertainty as the force of infection increased. In two of the three locations in Thailand where region and time matching seroprevalence and incidence data were available [34], the force of infection estimates obtained from the models fitted to incidence data and serology data had overlapping 95% credible intervals. In Ratchaburi the estimate obtained from seroprevalence data was smaller than that from incidence data (Fig 5).

## Discussion

From a literature search we selected 23 papers reporting age-stratified case notification data in 13 countries from 1978–2010. For each dataset we estimated dengue transmission intensity as quantified by the force of infection (*λ*) and the basic reproduction number (*R*_0_). Where possible we fitted to the cumulative incidence data as fitting to yearly incidence data gave less stable estimates (model fits to yearly incidence data are given in the S1 Text) The total force of infection (*λ*_*total*_) estimated from cumulative incidence data were then compared with previous *λ* estimates from seroprevalence data.

The incidence model presented in this paper provides a method for estimating dengue transmission intensity in areas where seroprevalence data are not available. Force of infection estimates and corresponding basic reproduction numbers varied widely across and within countries as expected, highlighting the heterogeneous nature of dengue transmission spatially and temporally. The majority of our *R*_0_ estimates ranged from 1 to 5, similar to our estimates obtained from seroprevalence data [13]. Similarly to our serology-based estimates, force of infection estimates were generally higher in South East Asia than for Latin America. Since we had no serotype-specific notification data, we assumed that all serotypes were equally transmissible and equally abundant. If serotype-specific notification data were available, serotype-specific forces of infection could be estimated. Although we assumed that dengue transmission intensity does not vary with age, is constant in time and equal for all serotypes in circulation, previous studies have shown that transmissibility can differ substantially not only between serotypes [13,54] but also seasonally, yearly [54], and spatially [55]. However, given the available data it was not possible to estimate serotype-specific or time-varying forces of infection. Multiple cross-sectional surveys or cohort studies are required to estimate how forces of infection have changed by age over time, and serotype-specific data are needed to resolve differences between serotypes.

Due to the lack of incidence and serology data collected in the same year and region, we matched cumulative incidence and serology datasets according to the year or region (see S1 Text). While overall estimates from incidence data were comparable with those derived from seroprevalence data, it would nonetheless be beneficial to validate this model with more incidence and serology datasets collected simultaneously in the same geographical location.

Generally, estimated reporting rates (*ρ*) in the Americas were higher than those in South East Asia with Singapore having the highest rate within South East Asia, consistent with their well-established dengue surveillance program [56]. Reporting rate estimates also varied within each country depending on survey year or survey region reflecting variation in healthcare and surveillance systems [19]. Reporting rates are also likely to change in response to recent or current epidemics which affect public awareness of dengue and thus healthcare seeking behaviour [57]. Additionally, in an epidemic year clinicians may preferentially diagnose a febrile illness as dengue without laboratory testing [58]. We hypothesised that severity or disease reporting differed by age group and estimated age-dependent reporting rates (*ρ*_*young*_ and *ρ*_*old*_) and the age at which reporting rates changed (*A*_*threshold*_). However due to the wide age bands of the available data, we were not able to explore this fully. Full details are given in the S1 Text.

Since the majority of notified dengue cases are diagnosed as secondary dengue infections [4,5,7,11,12,59], we assumed that the probability of detecting a primary case would be smaller than the probability of detecting a secondary case, and that the probability of detecting a tertiary or quaternary case would be smaller than the probability of detecting a primary case (*γ*_3_<*γ*_1_<*ρ*). The probability of detecting a primary case was consistently low relative to a secondary case (Fig 3) at less than 50%, the majority being under 25%. However, we were not able to estimate the probability of detecting a tertiary/quaternary case (relative to a primary case) from the available data. A prospective cohort study in Nicaragua found that the proportion of inapparent to symptomatic infection did not differ according to whether an individual had a primary, secondary, or tertiary infection [60].

Overall, the impact of cross-immunity and the contribution of tertiary and quaternary infections to onward transmission are not well quantified. While there is evidence that tertiary and quaternary infections occur [61,54], there is little quantitative data on the infectiousness or severity of such infections relative to primary and secondary infections. Additionally, clinically apparent tertiary or quaternary infections are not routinely reported, nor can they be tested for retrospectively [61]. Wikramaratna *et al*. showed that tertiary and quaternary infections allows for the high seroprevalence at very young ages observed in Haiti [62] and Nicaragua [63] better than when assuming complete protection after two heterologous infections [61].

Since the majority of dengue infections are mild or asymptomatic, even sensitive healthcare systems can substantially underestimate true rates of infection even for the supposedly more severe secondary infections, as shown by the low baseline reporting rates [11,3]. Furthermore, dengue has a wide spectrum of clinical manifestations making it difficult to accurately diagnose in the first instance [20]. Our estimates from Thailand (Fig 5) shows that even with data from the same location and year, it is difficult to make reliable comparisons between estimates obtained from seroprevalence and incidence data. We were also comparing force of infection estimates from seroprevalence data to those from incidence data from a single year (rather than cumulative incidence), which may have contributed to the observed discrepancy. Although incidence data are the most abundant form of data available on dengue transmission, surveillance systems and reporting procedures are not standardised within or across countries making it very difficult to reliably compare estimates [20]. Laboratory capacity and general public health infrastructure and surveillance systems vary widely and there is often no integration between private and public health sectors. With such variable data, it is very difficult to estimate dengue burden (or transmission intensity) consistently. Since non-serotype specific serological (IgG) surveys are relatively inexpensive to collect, it would be beneficial for such seroprevalence data to be collected routinely. Such data would provide better baseline estimates of overall transmission intensity against which incidence based-estimates could be calibrated to assess changes in transmission and identify weaknesses in surveillance systems.

## Supporting Information

S1 Text Supporting information file containing methods, results, and extra figures. (PDF)
