# Supplementary material for: Estimating Dengue Transmission Intensity from Case-Notification Data from Multiple Countries
Source: PLoS Negl Trop Dis. 2016 Jul 11;10(7):e0004833. doi: 10.1371/journal.pntd.0004833 (PMC4939939; doi:10.1371/journal.pntd.0004833)
Supplement: S1 Text — (PDF) [file pntd.0004833.s001.pdf]

Estimating dengue transmission intensity from case-notification data from multiple countries – Supporting Information

Authors: Natsuko Imai[1], Ilaria Dorigatti[1], Simon Cauchemez[2], Neil M. Ferguson[1]

Affiliations: 1. MRC Centre for Outbreak Analysis and Modelling, Department of Infectious Disease Epidemiology, Imperial College London, United Kingdom. **2.** Mathematical Modelling of Infectious Diseases Unit, Institut Pasteur, Paris, France.

1 Methods 2
1.1 Incidence Model 2
1.2 Calculating the Basic Reproduction Number (R_0) 7
1.3 Additional seroprevalence datasets and force of infection estimates 10
1.4 Weighted regression to compare estimates from seroprevalence and incidence data 11
2 Results 13
2.1 Parameter Estimates 13
2.2 DIC Comparison 38
2.3 Comparison of estimates obtained from seroprevalence and incidence data. 41
2.4 Model Fits by country 43
3 References 61

1 Methods

In the main text, only results from model 1B (cumulative incidence data) as described below are presented for clarity and to keep the paper concise.

1.1 Incidence Model

We consider a population stratified into *M* (where *M* varies by study) age groups and denote a_j and a_{j+1} the lower and upper age bounds respectively of age group *j* (*j=0,…, M-1*). Assuming life-long homologous protection after infection with any serotype, the number of infections an individual can acquire is finite (up to four). If fewer than four serotypes have circulated in any one area, then the number of infections an individual can have will change accordingly. Where four serotypes are known to be in circulation, and assuming a constant force of infection, λ, which is the same for all four serotypes, the incidence of primary infections caused by any one serotype in age group j, $I_1(j)$ is given by:

$$\begin{aligned} I_1(j) &= \int_{a_j}^{a_{j+1}} 4\lambda\left(e^{-\lambda a}\right)^4 da \\ &= \int_{a_j}^{a_{j+1}} 4\lambda e^{-4\lambda a} da = \left(e^{-4\lambda a_j} - e^{-4\lambda a_{j+1}}\right) \end{aligned} \tag{1.1}$$

The incidence of secondary infections in age group j, $I_2(j)$ is given by:

$$\begin{aligned} I_2(j) &= 4\int_{a_j}^{a_{j+1}} 3\lambda(1-e^{-\lambda a})\left(e^{-\lambda a}\right)^3 da \\ &= 4\left[e^{-3\lambda a_j} - e^{-3\lambda a_{j+1}}\right] - 3\left[e^{-4\lambda a_j} - e^{-4\lambda a_{j+1}}\right] \end{aligned} \tag{1.2}$$

The incidence of tertiary infections in an age group j, $I_3(j)$ is given by:

$$\begin{aligned} I_3(j) &= 4\int_{a_j}^{a_{j+1}} 3\lambda(1-e^{-\lambda a})^2\left(e^{-\lambda a}\right)^2 da \\ &= 4\int_{a_j}^{a_{j+1}} 3\lambda\left(e^{-2\lambda a} - 2e^{-3\lambda a} + e^{-4\lambda a}\right) da \\ &= 12\lambda\left(\left[-\frac{e^{-2\lambda a}}{2\lambda}\right]_{a_j}^{a_{j+1}} - \left[-\frac{2e^{-3\lambda a}}{3\lambda}\right]_{a_j}^{a_{j+1}} + \left[-\frac{e^{-4\lambda a}}{4\lambda}\right]_{a_j}^{a_{j+1}}\right) \\ &= 6\left[e^{-2\lambda a_j} - e^{-2\lambda a_{j+1}}\right] + 8\left[e^{-3\lambda a_{j+1}} - e^{-3\lambda a_j}\right] + 3\left[e^{-4\lambda a_j} - e^{-4\lambda a_{j+1}}\right] \end{aligned} \tag{1.3}$$

Finally, the incidence of quaternary infections in an age group j, $I_4(j)$ is given by:

$$
\begin{aligned}
I_4(j) &= 4\int_{a_j}^{a_{j+1}} \lambda\left(1-e^{-\lambda a}\right)^3 e^{-\lambda a} da \\
&= 4\lambda\int_{a_j}^{a_{j+1}} \left(e^{-\lambda a} - 3e^{-2\lambda a} + 3e^{-3\lambda a} - e^{-4\lambda a}\right) da \\
&= 4\lambda\left[\left[\frac{-e^{-\lambda a}}{\lambda}\right]_{a_j}^{a_{j+1}} - \left[-\frac{3e^{-2\lambda a}}{2\lambda}\right]_{a_j}^{a_{j+1}} + \left[-\frac{3e^{-3\lambda a}}{3\lambda}\right]_{a_j}^{a_{j+1}} - \left[-\frac{e^{-4\lambda a}}{4\lambda}\right]_{a_j}^{a_{j+1}}\right] \\
&= 4\left[e^{-\lambda a_j} - e^{-\lambda a_{j+1}} + e^{-3\lambda a_j} - e^{-3\lambda a_{j+1}}\right] + 6\left[e^{-2\lambda a_{j+1}} - e^{-2\lambda a_j}\right] + \left[e^{-4\lambda a_{j+1}} - e^{-4\lambda a_j}\right]
\end{aligned}
\tag{1.4}
$$

The total dengue infection incidence in an age group j, $T(j) = I_1 + I_2 + I_3 + I_4$ is, as expected:

$$
\begin{aligned}
T(j) &= \int_{a_j}^{a_{j+1}} \left[4\lambda\left(e^{-\lambda a}\right)^4 + 12\lambda(1-e^{-\lambda a})\left(e^{-\lambda a}\right)^3 + 12\lambda\left(1-e^{-\lambda a}\right)^2\left(e^{-\lambda a}\right)^2 + 4\lambda\left(1-e^{-\lambda a}\right)^3\left(e^{-\lambda a}\right)\right] da \\
&= 4\int_{a_j}^{a_{j+1}} \lambda e^{-\lambda a}\left(e^{-\lambda a} + \left[1-e^{-\lambda a}\right]\right)^3 da \\
&= \int_{a_j}^{a_{j+1}} 4\lambda e^{-\lambda a} da \\
&= 4e^{-\lambda a_j} - 4e^{-\lambda a_{j+1}}
\end{aligned}
$$

Ideally, we would allow forces of infection to vary by serotype (DENV-1 to DENV-4). However as serotype-specific data were not available, we assumed circulating serotypes were equally transmissible.

Model 1A and 1B: assuming a single reporting rate across all ages

The average annual incidence rate per person in an age group, $D(j)$, then becomes the weighted sum of the primary to quaternary infection rates:

$$
D(j) = \frac{1}{w(j)}\left\{\rho\left[I_2(j) + \gamma_1 I_1(j) + \gamma_3\left(I_3(j) + I_4(j)\right) + B\right]\right\} \tag{1.5}
$$

where $w(j) = a_{j+1} - a_j$ is the width of the age group j, ρ (baseline reporting rate) is the probability that a secondary infection results in a detected dengue case, γ_1 is the probability that a primary infection is detected relative to a secondary infection, and γ_3 is the probability that a tertiary or quaternary infection is detected relative to a primary infection. Here B is the baseline risk of infection used to represent any non-dengue related illnesses that are misdiagnosed as dengue (only estimated when cases have not been laboratory confirmed). Since we assumed that most symptomatic cases were secondary infections and that the majority of primary infections were asymptomatic, we estimated the probability of detecting a

primary infection relative to a secondary case and the probability of detecting a tertiary or quaternary infection relative to a primary case ($\rho > \gamma_1 > \gamma_3$). For model 1 we assumed a single baseline reporting rate (ρ) across all age groups and estimated four (or five) parameters: λ , ρ , γ_1 , γ_3 , (and B). Where multiple years of incidence data were available from the same survey we fitted each model variant to individual years (model 1A), and to the cumulative incidence (model 1B). When fitting to the cumulative incidence e.g. cumulative incidence over ten years, we multiplied the estimated annual disease incidence by 10 to take into account the survey period.

Models 2A and 2B: Assuming age-dependent reporting rates

Dengue reporting rates are likely to differ by age. In general, reporting rates may be higher in children, especially in South East Asia where dengue is generally a paediatric infection [1]. In the Americas where dengue is predominantly an adult disease, reporting rates may be higher in older children or adults. Therefore we allowed the baseline reporting rate ρ to differ depending on whether an individual was aged above or below a threshold age:

$$\begin{aligned} \rho &= \rho_{young}, \text{if } a < a_{threshold} \\ &= \rho_{old}, \text{if } a \geq a_{threshold} \end{aligned}$$

and estimated the value of $a_{threshold}$ for models 2A and 2B.

For model 2 we estimated six (or seven) parameters: λ , ρ_{young} , ρ_{old} , γ_1 , γ_3 , and $a_{threshold}$, (and B). Where multiple years of incidence data were available from the same survey we fitted each model variant to individual years (model 2A), and to the cumulative incidence (model 2B). When fitting to the cumulative incidence e.g. cumulative incidence over ten years, we multiplied the estimated annual disease incidence by 10 to take into account the survey period.

Priors

For all model variants (models 1A, 1B, 2A, and 2B) all parameters have uninformative uniform priors (Uniform([0,1])). For the age threshold the upper bound of the uniform distribution is the upper age bound of the oldest age group.

Likelihood

The expected number of cases per year in age-group j , (C_j) is:

$$C_j = n(j)D(j) \tag{1.6}$$

where $n(j)$ is the population size of age group j. Where population numbers were not available from the dataset, the population age-structure closest to the survey population was used (taken from census data or from United Nations estimates) [2].

We assumed that the number of cases reported in each age-group are Dirichlet-multinomially (DMN) distributed, as we expected the overall distribution of cases would be more overdispersed than what we would expect from a multinomial distribution [3]. The log-likelihood is given by:

$$\ln \ell \qquad {}_{j}\big) = -\left(\ln\Gamma\left(1/\psi + N\right) - \ln\Gamma\left(1/\psi\right)\right) \\ + \sum_{j=1}^{I}\left(\ln\Gamma\left(\frac{1}{\left(\psi / p_j\right)} + y_j\right) - \ln\Gamma\left(\frac{1}{\left(\psi / p_j\right)}\right)\right)$$

where ψ is the over-dispersion parameter characterising how different a DMN distribution is from the corresponding multinomial distribution (MN) with the same category probabilities (the larger the ψ, the greater the difference) and y_j is the observed number of cases in age class j. The probabilities (p_j) are then calculated as the expected proportion of cases in one age group relative to the total number of cases across all age groups.

$$p_j = \frac{C_j}{\sum_j C_j}$$

We then assumed that the total number of cases across all ages (N) was Poisson distributed:

$P(\mu = N) = \dfrac{\mu^N e^{-\mu}}{N!}$ where μ is the total expected number of cases across all age groups $\left(\sum_j C_j\right)$.

The full log-likelihood is then:

$$\ln \ell \qquad {}_{j}\big) = -\left(\ln\Gamma\left(1/\psi + N\right) - \ln\Gamma\left(1/\psi\right)\right) \\ + \sum_{j=1}^{I}\left(\ln\Gamma\left(\frac{1}{\left(\psi / p_j\right)} + y_j\right) - \ln\Gamma\left(\frac{1}{\left(\psi / p_j\right)}\right)\right) \\ + N\ln(\mu) - \mu - \ln(N!)$$

Expected incidence when there are fewer than four serotypes in circulation

When there are fewer than four serotypes in circulation, the maximum number of infections an individual can acquire also changes accordingly. Assuming an equal force of infection for all serotypes in circulation, the incidences are given by:

1) One serotype in circulation

With only one serotype in circulation, individuals will only be infected once in their lifetime. Thus the incidence of primary infection in age group j (with lower age bound a_j and upper age bound a_{j+1}) $I_1(j)$ is given by:

$$I_1(j) = \int_{a_j}^{a_{j+1}} \lambda\left(e^{-\lambda a}\right)da$$
$$= e^{-\lambda a_j} - e^{-\lambda a_{j+1}}$$

The incidence of disease in age group j is then given by:

$$D(j) = \frac{1}{w(j)}\left\{\rho\left[I_1(j) + B\right]\right\}.$$

2) Two serotypes in circulation

With two serotypes in circulation, individuals can have up to two infections in their lifetime.

The incidence of primary infection in age group j (with lower age bound a_j and upper age bound a_{j+1}) $I_1(j)$ is given by:

$$I_1(j) = 2\int_{a_j}^{a_{j+1}} \lambda\left(e^{-\lambda a}\right)^2 da$$
$$= \left(e^{-2\lambda a_j} - e^{-2\lambda a_{j+1}}\right)$$

and the incidence of secondary infection in age group j, $I_2(j)$ is given by:

$$I_2(j) = 2\int_{a_j}^{a_{j+1}} \lambda\left[1 - e^{-\lambda a}\right]\left(e^{-\lambda a}\right)da$$
$$= 2\lambda\left[\frac{e^{-\lambda a_j}}{\lambda} - \frac{e^{-\lambda a_{j+1}}}{\lambda} + \frac{e^{-2\lambda a_{j+1}}}{2\lambda} - \frac{e^{-2\lambda a_j}}{2\lambda}\right]$$
$$= 2\left[e^{-\lambda a_j} - e^{-\lambda a_{j+1}}\right] - \left[e^{-2\lambda a_j} - e^{-2\lambda a_{j+1}}\right]$$

The incidence of disease in age group j is then given by:

$$D(j) = \frac{1}{w(j)}\left\{\rho\left[I_2(j) + \gamma_1 I_1(j) + B\right]\right\}.$$

3) Three serotypes in circulation

With three serotypes in circulation, individuals can have a maximum of 3 infections.

The incidence of primary infection in age group j (with lower age bound a_j and upper age bound a_{j+1}) $I_1(j)$ is given by:

$$I_1(j) = 3\int_{a_j}^{a_{j+1}} \lambda\left(e^{-\lambda a}\right)^3 da$$
$$= \left(e^{-3\lambda a_j} - e^{-3\lambda a_{j+1}}\right)$$

The incidence of secondary infection in age group j, $I_2(j)$ is given by:

$$I_2(j) = 3\int_{a_j}^{a_{j+1}} 2\lambda\left[1-e^{-\lambda a}\right]\left(e^{-\lambda a}\right)^2 da$$
$$= 6\lambda\left[\frac{e^{-2\lambda a_j}}{2\lambda} - \frac{e^{-2\lambda a_{j+1}}}{2\lambda} + \frac{e^{-3\lambda a_{j+1}}}{3\lambda} - \frac{e^{-3\lambda a_j}}{3\lambda}\right]$$
$$= 3\left[e^{-2\lambda a_j} - e^{-2\lambda a_{j+1}}\right] - 2\left[e^{-3\lambda a_j} - e^{-3\lambda a_{j+1}}\right]$$

and the incidence of tertiary infection in age group j, $I_3(j)$ is given by:

$$I_3(j) = 3\int_{a_j}^{a_{j+1}} \lambda\left[1-e^{-\lambda a}\right]^2 \left(e^{-\lambda a}\right) da$$
$$= 3\lambda\left[\frac{e^{-\lambda a_j}}{\lambda} - \frac{e^{-\lambda a_{j+1}}}{\lambda} + \frac{2e^{-2\lambda a_{j+1}}}{2\lambda} - \frac{2e^{-2\lambda a_j}}{2\lambda} + \frac{e^{-3\lambda a_j}}{3\lambda} - \frac{e^{-3\lambda a_j}}{3\lambda}\right]$$
$$= 3\left[e^{-\lambda a_j} - e^{-\lambda a_{j+1}}\right] - 3\left[e^{-2\lambda a_j} - e^{-2\lambda a_{j+1}}\right] + \left[e^{-3\lambda a_j} - e^{-3\lambda a_{j+1}}\right]$$

The incidence of disease in age group j is then given by:

$$D(j) = \frac{1}{w(j)}\left\{\rho\left[I_2(j) + \gamma_1(I_1(j) + I_3(j)) + B\right]\right\}.$$

1.2 Calculating the Basic Reproduction Number (R_0)

We assumed dengue was at endemic equilibrium and that the force of infection (λ) was constant in time. We additionally assumed that all serotypes in circulation were equally transmissible. We calculated the serotype-specific basic reproduction number under the following two assumptions to match previous estimates from seroprevalence data, as described in Imai *et al* [4].

Assumption 1 – individuals can be infected four times, there is no cross-immunity between serotypes. The basic reproduction number for serotype i is given by [5]:

$$R_0 = \frac{1}{1-\int_0^\infty f(j) z_i(a) da}$$

where $f(a)$ is the proportion of the population aged a. $z_i(a)$ is the proportion of population aged a seropositive to serotype i and is given by:

$$z_i(a) = 1 - e^{-\frac{\lambda}{n}a}$$

where n is the number of serotypes in circulation.

Integrating between a_j and a_{j+1}:

$$R_0 = \frac{1}{1 - \int_{a_j}^{a_{j+1}} f(a)\left[1 - e^{-\frac{\lambda}{n}a}\right] da}$$

$$= \frac{1}{1 - \int_{a_j}^{a_{j+1}} \left[f(a) + f(a)e^{-\frac{\lambda}{n}a}\right] da}$$

$$= \frac{1}{1 - \int_{a_j}^{a_{j+1}} f(a)da + \int_{a_j}^{a_{j+1}} f(a)e^{-\frac{\lambda}{n}a} da}$$

$\int_{a_j}^{a_{j+1}} f(a)da$ = 1 so we are left with:

$$R_0 = \frac{1}{\int_{a_j}^{a_{j+1}} f(a)e^{-\frac{\lambda}{n}a} da}$$

$$= \frac{1}{\sum_j \frac{f(a_{j+1} \to a_j)}{a_{j+1} - a_j}\left[-\frac{n\exp[-\lambda a/n]}{\lambda}\right]_{a_j}^{a_{j+1}}}$$

$$= \frac{1}{\sum_j \frac{f(a_{j+1} \to a_j)}{a_{j+1} - a_j}\frac{n}{\lambda}\left[\exp\left[-\lambda a_j/n\right] - \exp\left[-\lambda a_{j+1}/n\right]\right]}$$

$$= \frac{\lambda}{\sum_j \frac{f(a_{j+1} \to a_j)}{a_{j+1} - a_j} n\left[\exp\left[-\lambda a_j/n\right] - \exp\left[-\lambda a_{j+1}/n\right]\right]}$$

Assumption 2 – Complete immunity after secondary infection.

If only primary and secondary infections can occur, we can relax the assumption of no cross-immunity between serotypes. The basic reproduction number (same for any serotype) is given by:

$$R_0 = \frac{1}{\int_0^\infty f(a)\left[x(a)+(n-1)z(a)\right]da}$$

where $f(a)$ is the proportion of the population aged a, n is the number of serotypes in circulation, $x(a)$ is the proportion of the population seronegative at age a calculated by: $x(a)=\exp[-\lambda a]$, and $z_i(a)$ is the proportion of the population seropositive for serotype i at age a calculated by:

$$\begin{aligned} z_i(a) &= \left[1-\exp[-\lambda a/n]\right]\left[\exp\left[-(n-1)\lambda a/n\right]\right] \\ &= \exp\left[-(n-1)\lambda a/n\right]-\exp[-n\lambda a/n] \\ &= \exp\left[-(n-1)\lambda a/n\right]-\exp[-\lambda a] \end{aligned}$$

Integrating between ages a_j and a_{j+1}:

$$\begin{aligned} R_0 &= \frac{1}{\int_{a_j}^{a_{j+1}} f(a)\left[x(a)+(n-1)z(a)\right]da} \\ &= \frac{1}{\int_{a_j}^{a_{j+1}} f(a)\left[(n-1)\exp\left[-(n-1)\lambda a/n\right]-(n-2)\exp[-\lambda a]\right]da} \\ &= \frac{1}{\sum_j \frac{f(a_{j+1}\to a_j)}{a_{j+1}-a_j}\left[\begin{array}{l} \frac{n\left(\exp\left[-(n-1)\lambda a_j/n\right]-\exp\left[-(n-1)\lambda a_{j+1}/n\right]\right)}{\lambda} - \\ (n-2)\left(\frac{\left(\exp\left[-\lambda a_j\right]-\exp\left[-\lambda a_{j+1}\right]\right)}{\lambda}\right) \end{array}\right]} \\ &= \frac{\lambda}{\sum_j \frac{p(a_{j+1}\to a_j)}{a_{j+1}-a_j}\left[n\left(\exp\left[-(n-1)\lambda a_j/n\right]-\exp\left[-(n-1)\lambda a_{j+1}/n\right]\right)-(n-2)\left(\exp\left[-\lambda a_j\right]-\exp\left[-\lambda a_{j+1}\right]\right)\right]} \end{aligned}$$

1.3 Additional seroprevalence datasets and force of infection estimates

In addition to the force of infection estimates calculated from the seroprevalence datasets described in Imai *et al.* [4], we estimated λ using model A (as described in [4]) from four additional datasets from Brazil and Taiwan (Table S1). Model fits are shown in Fig. S1.

Table S1: Parameter estimates from extra seroprevalence datasets

Country	Author	Region	Urban/Rural	λ force of infection (95% CrI)	γ over-dispersion* (95% CrI)	R_0		Ref
						Assumption 1 (95% CrI)	Assumption 2 (95% CrI)	
Brazil	Fernando	Paco do Lumiar<br>Sao Jose de Ribamar<br>Estado do Maranho	Urban	0·013 (0·011-0·021)	0·070 (0·044-0·270)	1·09 (1·08-1·15)	1·12 (1·10-1·21)	[6]
Brazil	da Silva-Nunes	Amazon	Rural	0·008 (0·007-0·017)	0·026 (0·009-0·319)	1·05 (1·05-1·12)	1·06 (1·05-1·16)	[7]
Brazil	Lima	Campinas	Urban	0·007 (0·006-0·016)	0·111 (0·079-0·319)	1·05 (1·04-1·11)	1·06 (1·05-1·15)	[8]
Taiwan	Shu	Liuchiu Hsiang	Urban	0·026 (0·023-0·037)	0·157 (0·112-0·380)	1·24 (1·21-1·34)	1·38 (1·32-1·60)	[9]

*over-dispersion parameter representing heterogeneity in the seroprevalence with age-group.

Fig. S1: Model fits from non-serotype specific model A (as described previously [4]**) fitted to the extra seroprevalence datasets described in Table S1. 95% exact confidence intervals around data points, posterior median (line) and 95% credible interval (shaded area) shown.**

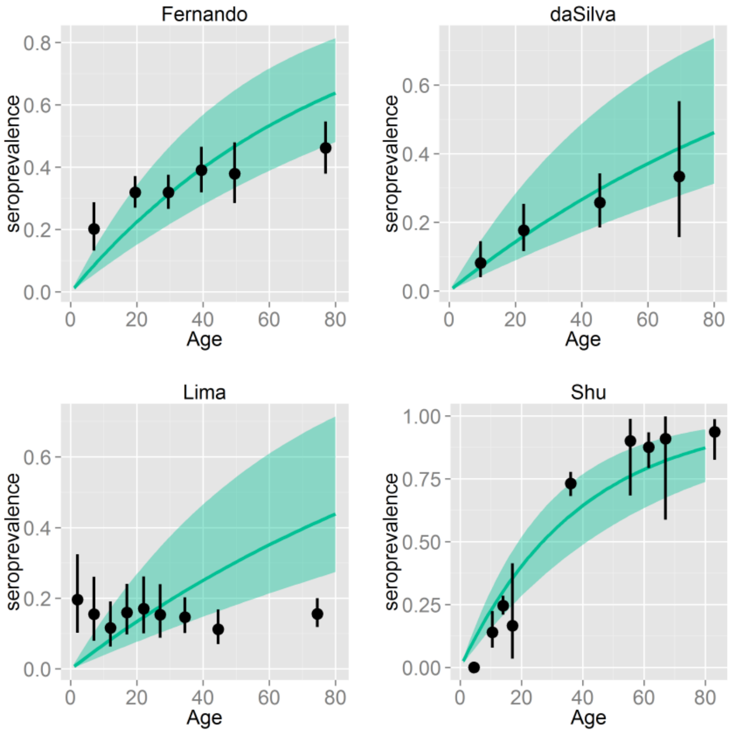

1.4 Weighted regression to compare estimates from seroprevalence and incidence data

We used a linear regression to compare the force of infection estimates obtained from seroprevalence (λ_{sero}) and incidence (λ_{inc}) data using the method described by Ripley and Thompson [10], which explicitly accounts for measurement errors in both the x and y to estimate the MLE line. This was implemented using the *deming* package in R [11]. Since seroprevalence and incidence data collected in the same year and geographical location were not available, the serology datasets were matched to corresponding incidence datasets as closely as possible by country, region, and survey year (Table S2). Since seroprevalence data represent all past infections, we compared these estimates to those obtained from cumulative incidence data where possible.

Confidence intervals for the regression line were estimated using the jackknife estimate of the variance-covariance matrix between the estimated intercept and slope of the regression. The areas of the symbols on the plots (main text Fig. 4) are proportional to the point weights, which correspond to the reciprocal of the variance of the error term in the linear regression. The larger circles indicate greater weight, i.e. smaller error.

Table S2: Matched serology and incidence datasets

	Incidence Datasets					Serology Datasets			
Country	Author	Survey Year	Region	Diagnosis	Ref	Author	Survey Year	Region	Ref
Brazil	Penna	2001/06	Amazon	Clinical	[12]	Silva-Nunez	2004	Ramal do Granada (Acre State Amazon)	[13]
Brazil	Cordeiro	2002/06	Pernambuco (Recife)	Lab confirmed	[14]	Braga	2005/06	Recife (NE)	[15]
Brazil	Cardoso	2000/09	Vitoria (~Rio)	Lab confirmed	[16]	Lima	1998	Sao Paulo	[17]
Brazil	Cordeiro	1995/01	Pernambuco (Recife)	Lab confirmed	[14]	Fernando	1996	Paco do Lumiar, Sao Jose de Ribamar, Estado do Maranho	[6]
Laos	Anker	2000/06	National	Clinical	[18]	Vallee	2006	Vientiane	[19]
Nicaragua	Hammond	1999/01	Leon	Clinical/Lab	[20]	Balmaseda	2001/06	Managua	[21]
Singapore	Anker	1999/05	National	Clinical/Lab	[18]	Yew	2004	National	[22]
Singapore	Ler	2000/07	National	Lab confirmed	[23]	Yap	2007	National	[24]
Taiwan	Lin	2003/09	Kaohsiung City	Lab confirmed	[25]	Shu	1997-1998	Liuchiu Hsiang	[9]
Thailand	Thai MoH*	2000	Bangkok^	Lab confirmed	[26]	Perret	2000	Bangkok	[27]
Thailand	Thai MoH*	2000	Ratchaburi^	Lab confirmed	[26]	Tuntaprasart	2000	Ratchaburi	[28]
Thailand	Thai MoH*	2010	Rayong	Lab confirmed	[26]	Rodriguez-Barraquer	2010	Rayong	[29]
Vietnam	Cuong	1998/09	Hanoi	Lab confirmed	[30]	Bartley	1996-1997	Dong Thap	[31]

*Although not cumulative incidence, these datasets were retained for analysis as they matched the corresponding seroprevalence surveys exactly by year and region. ^The two datasets reported DHF cases only; we therefore assumed all observed cases were due to secondary infections.

2 Results

2.1 Parameter Estimates

As expected, force of infection estimates varied widely between countries, with less variation seen within countries. Figures S2 and S3 show the distribution of the total force of infection (λ_{total}) grouped by country (calculated by multiplying the serotype-specific force of infection by the number of serotypes in circulation) from models 1A (single reporting rate, ρ, fitted to yearly incidence data) and 2A (age-dependent reporting rate, ρ_y and ρ_o, fitted to yearly incidence data) respectively. Individual estimates are given in Table S3 and Table S4.

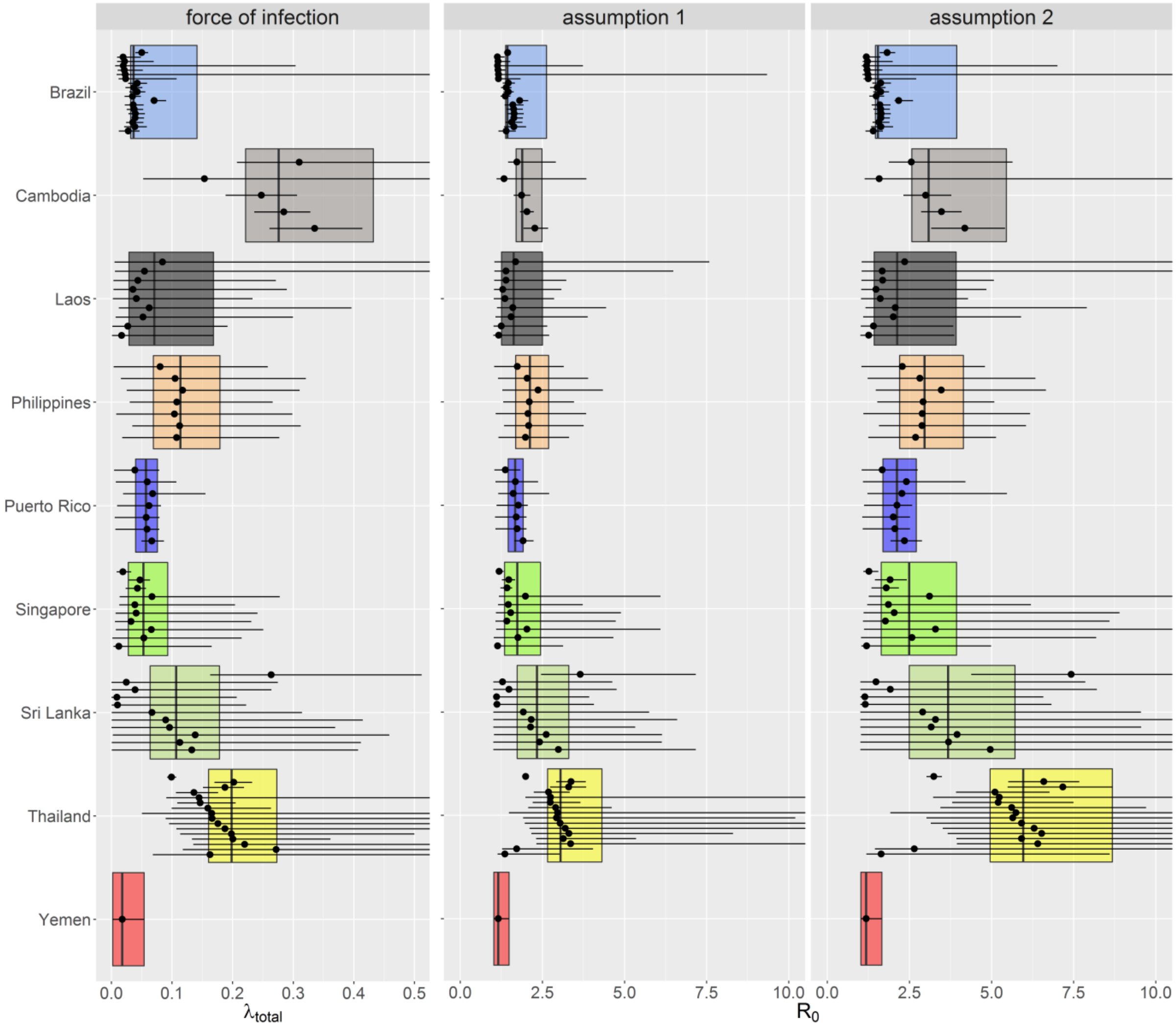

Figure S2: Total force of infection and corresponding R_0 estimates from model 1A (single reporting rate) and fitted to yearly incidence data grouped by country. Each point represents the posterior median estimate and the error bars the 95% CrI. The box represents the country-specific central estimate calculated by taking the mean values of the MCMC output for each country (the line and limits of the box represents the posterior median and the 95% CrI respectively).

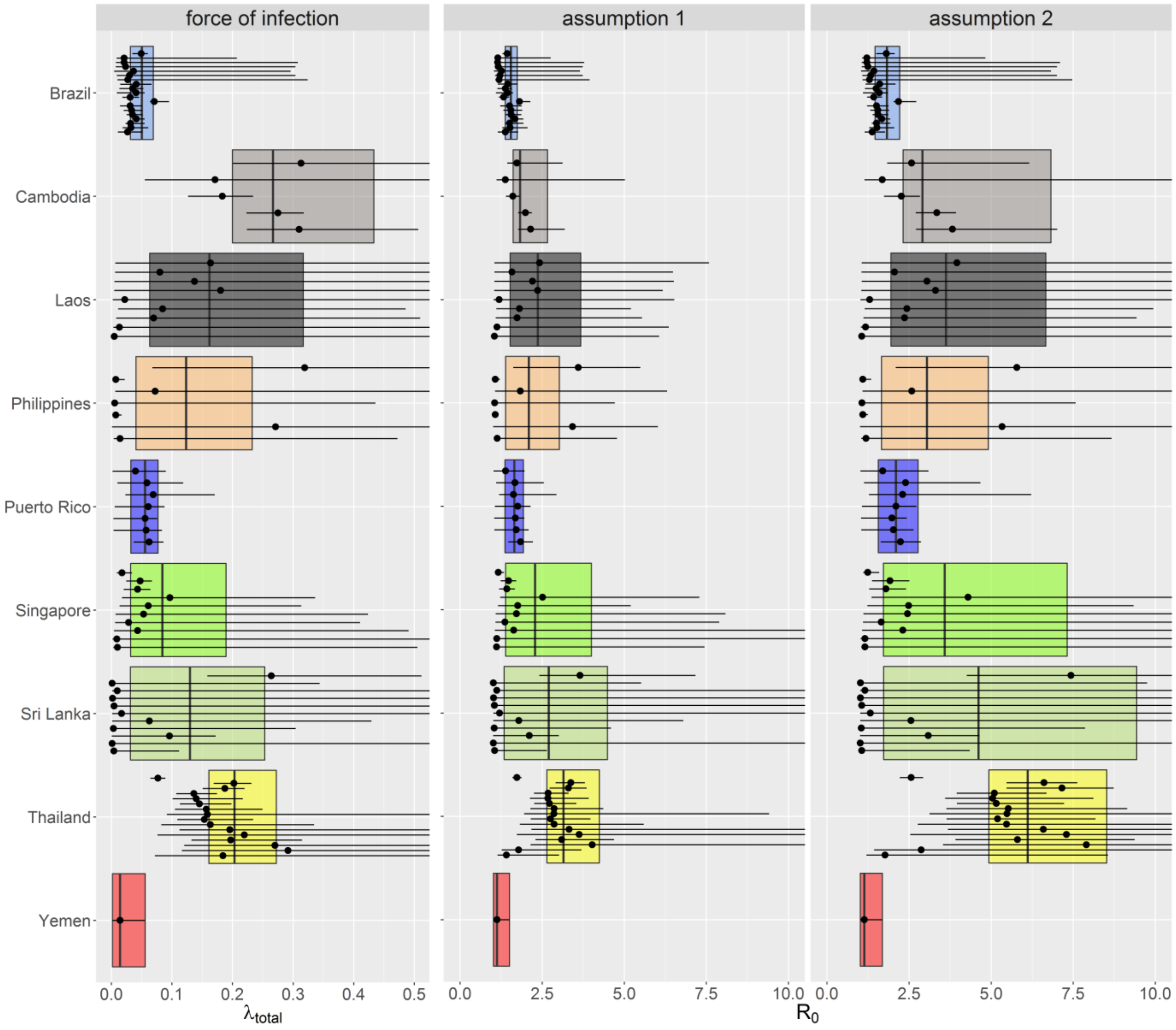

Figure S3: Total force of infection and corresponding R_0 estimates from model 2A (age-dependent reporting rate) and fitted to yearly incidence data grouped by country. Each point represents the posterior median estimate and the error bars the 95% CrI. The box represents the country-specific central estimate calculated by taking the mean values of the MCMC output for each country (the line and limits of the box represents the posterior median and the 95% CrI respectively).

Table S3: Summary parameter estimates from model 1 fitted to yearly incidence data (model 1A).

Author [Ref]	Year	Country	λ (95% CrI)	ρ (95% CrI)	β (95% CrI)	ψ (95% CrI)	γ_1 (95% CrI)	γ_3 (95% CrI)	λ_{tot} (95% CrI)	R_0 * (95% CrI)		LnL
										1	2	
Cordeiro [32]	1995	Brazil	0.014 (0.006-0.023)	0.263 (0.147-0.827)	NA	0.012 (0.004-0.067)	0.024 (0.003-0.058)	0.550 (0.038-0.980)	0.028 (0.012-0.046)	1.40 (1.17-1.69)	1.40 (1.17-1.69)	-17854
	1996		0.019 (0.011-0.030)	0.388 (0.262-0.810)	NA	0.010 (0.003-0.048)			0.039 (0.021-0.059)	1.64 (1.33-2.01)	1.64 (1.33-2.01)	-40383
	1997		0.018 (0.012-0.026)	0.614 (0.412-0.954)	NA	0.009 (0.003-0.039)			0.035 (0.024-0.053)	1.57 (1.38-1.89)	1.57 (1.38-1.89)	-57709
	1998		0.020 (0.017-0.027)	0.882 (0.649-0.994)	NA	0.005 (0.001-0.030)			0.039 (0.034-0.054)	1.63 (1.54-1.90)	1.63 (1.54-1.90)	-93278
	1999		0.020 (0.014-0.028)	0.574 (0.417-0.844)	NA	0.005 (0.002-0.023)			0.040 (0.028-0.055)	1.64 (1.44-1.92)	1.64 (1.44-1.92)	-64112
	2000		0.019 (0.013-0.026)	0.458 (0.331-0.726)	NA	0.006 (0.002-0.033)			0.038 (0.025-0.053)	1.63 (1.40-1.91)	1.63 (1.40-1.91)	-50580
	2001		0.018 (0.011-0.027)	0.289 (0.199-0.515)	NA	0.002 (0.001-0.007)			0.036 (0.022-0.053)	1.60 (1.36-1.93)	1.60 (1.36-1.93)	-31022
	2002		0.024 (0.022-0.030)	0.963 (0.845-0.999)	NA	0.042 (0.016-0.154)			0.071 (0.065-0.090)	1.81 (1.73-2.07)	2.18 (2.07-2.61)	-209223
	2003		0.012 (0.007-0.016)	0.380 (0.280-0.660)	NA	0.008 (0.002-0.034)			0.035 (0.022-0.049)	1.37 (1.23-1.53)	1.48 (1.28-1.73)	-47533
	2004		0.014 (0.010-0.019)	0.075 (0.059-0.111)	NA	0.005 (0.002-0.033)			0.043 (0.030-0.056)	1.46 (1.31-1.63)	1.63 (1.40-1.88)	-11581
	2005		0.012 (0.008-0.017)	0.173 (0.128-0.297)	NA	0.007 (0.002-0.036)			0.037 (0.023-0.051)	1.40 (1.24-1.56)	1.53 (1.30-1.78)	-23548
	2006		0.014 (0.009-0.020)	0.213 (0.161-0.335)	NA	0.009 (0.003-0.046)			0.043 (0.028-0.059)	1.46 (1.29-1.66)	1.63 (1.37-1.94)	-34400
Penna [12]	2001		0.006 (0.003-0.027)	0.437 (0.043-0.937)	0.008 (0.001-0.620)	0.024 (0.003-0.397)			0.024 (0.012-0.108)	1.17 (1.08-1.84)	1.25 (1.11-2.71)	-14639
	2002		0.006 (0.002-0.202)	0.268 (0.022-0.851)	0.009 (0.001-0.792)	0.033 (0.002-0.510)			0.023 (0.009-0.808)	1.16 (1.06-9.34)	1.24 (1.08-18.84)	-9046
	2003		0.005 (0.003-0.013)	0.363 (0.107-0.859)	0.006 (0.001-0.108)	0.021 (0.003-0.269)			0.022 (0.011-0.052)	1.15 (1.07-1.38)	1.22 (1.09-1.68)	-10371
	2004		0.005 (0.002-0.076)	0.199 (0.011-0.893)	0.008 (0.001-0.940)	0.047 (0.005-0.656)			0.020 (0.006-0.304)	1.14 (1.04-3.74)	1.20 (1.05-7.01)	-5540

*Assumption 1: individuals can be infected up to four times. Assumption 2: individuals are immune after two infections.

Table S3 continued (2/5).

Author [Ref]	Year	Country	λ (95% CrI)	ρ (95% CrI)	β (95% CrI)	ψ (95% CrI)	γ_1 (95% CrI)	γ_3 (95% CrI)	λ_{tot} (95% CrI)	R_0 * (95% CrI)		LnL
										1	2	
Penna [12]	2005	Brazil	0.006 (0.002-0.018)	0.282 (0.044-0.883)	0.008 (0.001-0.312)	0.035 (0.004-0.349)	0.024 (0.003-0.058)	0.550 (0.038-0.980)	0.022 (0.010-0.070)	1.15 (1.07-1.52)	1.23 (1.08-1.99)	-8556
	2006		0.005 (0.002-0.012)	0.315 (0.071-0.818)	0.004 (0.001-0.117)	0.014 (0.001-0.235)			0.019 (0.010-0.049)	1.13 (1.07-1.35)	1.19 (1.08-1.62)	-7823
Vong [33]	2006	Cambodia	0.084 (0.065-0.104)	0.189 (0.164-0.200)	NA	0.014 (0.007-0.030)	0.128 (0.068-0.279)	0.508 (0.030-0.977)	0.335 (0.261-0.414)	2.27 (1.93-2.67)	4.19 (3.17-5.42)	-20854
	2007		0.071 (0.059-0.082)	0.960 (0.844-0.999)	NA	0.006 (0.003-0.012)			0.285 (0.236-0.328)	2.03 (1.82-2.24)	3.48 (2.86-4.09)	-110705
	2008		0.062 (0.047-0.077)	0.302 (0.263-0.327)	NA	0.015 (0.008-0.030)			0.247 (0.188-0.307)	1.87 (1.63-2.13)	3.00 (2.32-3.78)	-34280
Wichmann [34]	2006		0.038 (0.013-0.194)	0.337 (0.186-0.946)	NA	0.204 (0.008-0.944)			0.154 (0.053-0.775)	1.32 (1.11-3.84)	1.58 (1.14-13.22)	-31
	2007		0.078 (0.052-0.172)	0.897 (0.745-0.994)	NA	0.129 (0.002-0.871)			0.310 (0.207-0.687)	1.72 (1.46-2.91)	2.56 (1.87-5.64)	-543
Anker [18]	2000	Laos	0.004 (0.000-0.042)	0.045 (0.009-0.879)	NA	0.174 (0.009-0.895)	0.265 (0.062-0.705)	0.589 (0.046-0.981)	0.017 (0.001-0.168)	1.17 (1.01-2.71)	1.26 (1.01-3.87)	-1051
	2001		0.007 (0.001-0.048)	0.076 (0.019-0.906)	NA	0.171 (0.006-0.912)			0.027 (0.002-0.192)	1.25 (1.02-2.65)	1.41 (1.02-3.83)	-3630
	2002		0.013 (0.002-0.075)	0.132 (0.044-0.687)	NA	0.212 (0.011-0.915)			0.052 (0.007-0.300)	1.55 (1.07-3.88)	2.01 (1.09-5.90)	-8186
	2003		0.016 (0.003-0.099)	0.173 (0.060-0.622)	NA	0.278 (0.028-0.934)			0.062 (0.012-0.396)	1.60 (1.12-4.44)	2.07 (1.16-7.90)	-17292
	2004		0.010 (0.001-0.058)	0.049 (0.016-0.769)	NA	0.140 (0.002-0.873)			0.041 (0.003-0.233)	1.36 (1.02-2.86)	1.62 (1.03-4.29)	-3280
	2005		0.009 (0.001-0.072)	0.090 (0.023-0.854)	NA	0.190 (0.013-0.894)			0.036 (0.004-0.289)	1.29 (1.03-3.08)	1.49 (1.03-4.84)	-5243
	2006		0.011 (0.001-0.068)	0.085 (0.027-0.829)	NA	0.201 (0.013-0.897)			0.044 (0.004-0.271)	1.39 (1.03-3.23)	1.68 (1.04-5.08)	-5948
Khampapongpane [38]	2010		0.014 (0.001-0.161)	0.102 (0.024-0.473)	0.124 (0.004-0.951)	0.080 (0.015-0.506)			0.055 (0.006-0.644)	1.39 (1.04-6.49)	1.67 (1.05-12.46)	-33953
Prasith [37]	2010		0.021 (0.001-0.178)	0.088 (0.040-0.561)	0.376 (0.009-0.971)	0.386 (0.069-0.934)			0.085 (0.005-0.712)	1.69 (1.04-7.58)	2.36 (1.04-13.03)	-22762

*Assumption 1: individuals can be infected up to four times. Assumption 2: individuals are immune after 2 infections.

Table S3 continued (3/5)

Author [Ref]	Year	Country	λ (95% CrI)	ρ (95% CrI)	β (95% CrI)	ψ (95% CrI)	γ_1 (95% CrI)	γ_3 (95% CrI)	λ_{tot} (95% CrI)	R_0 * (95% CrI)		LnL
										1	2	
Anker [18]	1998	Philippines	0.027 (0.004-0.069)	0.028 (0.015-0.073)	NA	0.168 (0.002-0.897)	0.070 (0.003-0.563)	0.537 (0.033-0.979)	0.108 (0.018-0.277)	1.98 (1.16-3.32)	2.69 (1.25-5.14)	-33865
	1999		0.028 (0.009-0.078)	0.007 (0.003-0.014)	NA	0.166 (0.002-0.904)			0.113 (0.035-0.312)	2.09 (1.33-3.76)	2.89 (1.57-6.05)	-9156
	2001		0.026 (0.002-0.075)	0.018 (0.008-0.105)	NA	0.223 (0.008-0.937)			0.104 (0.008-0.299)	2.06 (1.08-3.84)	2.89 (1.10-6.17)	-23843
	2002		0.027 (0.008-0.067)	0.016 (0.008-0.034)	NA	0.309 (0.037-0.930)			0.108 (0.030-0.266)	2.10 (1.30-3.46)	2.92 (2.52-5.09)	-16213
	2003		0.029 (0.006-0.078)	0.026 (0.013-0.052)	NA	0.340 (0.049-0.921)			0.117 (0.026-0.311)	2.37 (1.28-4.34)	3.47 (1.48-6.66)	-28567
	2004		0.026 (0.004-0.080)	0.016 (0.009-0.041)	NA	0.207 (0.006-0.933)			0.105 (0.016-0.321)	2.04 (1.15-3.90)	2.82 (1.23-6.34)	-22020
	2005		0.020 (0.001-0.064)	0.030 (0.013-0.639)	NA	0.243 (0.003-0.918)			0.081 (0.004-0.258)	1.74 (1.04-3.15)	2.29 (1.04-4.80)	-32194
Rigau-Perez [35]	1994	Puerto Rico	0.022 (0.017-0.029)	0.348 (0.236-0.487)	NA	0.005 (0.002-0.017)	0.413 (0.110-0.884)	0.637 (0.067-0.985)	0.067 (0.050-0.087)	1.91 (1.65-2.23)	2.35 (1.93-2.88)	-50388
	1995		0.020 (0.002-0.026)	0.033 (0.022-0.355)	NA	0.007 (0.001-0.112)			0.059 (0.007-0.079)	1.73 (1.07-2.02)	2.06 (1.08-2.52)	-3062
	1996		0.019 (0.002-0.027)	0.030 (0.019-0.358)	NA	0.009 (0.001-0.160)			0.058 (0.006-0.080)	1.71 (1.06-2.02)	2.01 (1.07-2.52)	-2697
	1997		0.021 (0.003-0.027)	0.035 (0.023-0.249)	NA	0.006 (0.001-0.125)			0.062 (0.010-0.082)	1.77 (1.11-2.06)	2.12 (1.12-2.58)	-3370
Ramos [36]	2006		0.017 (0.005-0.039)	0.362 (0.202-0.807)	NA	0.046 (0.003-0.443)			0.068 (0.019-0.155)	1.62 (1.15-2.71)	2.27 (1.22-5.47)	-203
Sharp [37]	2010		0.015 (0.002-0.027)	0.165 (0.101-0.845)	NA	0.030 (0.012-0.116)			0.059 (0.007-0.107)	1.68 (1.07-2.37)	2.41 (1.09-4.21)	-24291
Tomashek [38]	2007		0.010 (0.001-0.020)	0.053 (0.029-0.420)	NA	0.026 (0.010-0.083)			0.039 (0.005-0.079)	1.37 (1.04-1.83)	1.67 (1.05-2.76)	-7042
Anker [18]	1999	Singapore	0.003 (0.001-0.041)	0.150 (0.026-0.886)	NA	0.152 (0.004-0.896)	0.053 (0.007-0.159)	0.549 (0.027-0.976)	0.013 (0.003-0.166)	1.14 (1.04-3.13)	1.20 (1.04-4.99)	-521
	2000		0.013 (0.001-0.054)	0.023 (0.015-0.832)	NA	0.228 (0.012-0.942)			0.054 (0.002-0.215)	1.76 (1.02-4.66)	2.58 (1.03-8.19)	-362
	2001		0.016 (0.002-0.063)	0.061 (0.048-0.436)	NA	0.292 (0.013-0.955)			0.066 (0.007-0.251)	2.03 (1.09-6.10)	3.30 (1.12-11.86)	-940

*Assumption 1: individuals can be infected up to four times. Assumption 2: individuals are immune after two infections.

Table S3 continued (4/5)

Author [Ref]	Year	Country	λ (95% CrI)	ρ (95% CrI)	β (95% CrI)	ψ (95% CrI)	γ_1 (95% CrI)	γ_3 (95% CrI)	λ_{tot} (95% CrI)	R_0 * (95% CrI)		LnL
										1	2	
Anker [18]	2002	Singapore	0.008<br>(0.001-0.058)	0.129<br>(0.065-0.916)	NA	0.215<br>(0.011-0.945)	0.053<br>(0.007-0.159)	0.549<br>(0.027-0.976)	0.032<br>(0.006-0.231)	1.42<br>(1.07-4.73)	1.77<br>(1.09-8.59)	-1506
	2003		0.010<br>(0.003-0.060)	0.136<br>(0.079-0.834)	NA	0.249<br>(0.012-0.953)			0.041<br>(0.007-0.241)	1.53<br>(1.08-4.89)	2.04<br>(1.11-8.90)	-1859
	2004		0.010<br>(0.003-0.051)	0.342<br>(0.169-0.904)	NA	0.260<br>(0.014-0.952)			0.039<br>(0.013-0.204)	1.46<br>(1.15-3.73)	1.86<br>(1.22-6.20)	-4135
	2005		0.017<br>(0.003-0.069)	0.289<br>(0.211-0.843)	NA	0.310<br>(0.013-0.950)			0.067<br>(0.014-0.278)	1.98<br>(1.17-6.09)	3.11<br>(1.26-11.75)	-6857
Koh [39]	2005		0.011<br>(0.006-0.014)	0.266<br>(0.220-0.424)	NA	0.007<br>(0.002-0.035)			0.043<br>(0.024-0.057)	1.43<br>(1.22-1.58)	1.79<br>(1.35-2.18)	-20559
Ler [23]	2005		0.012<br>(0.007-0.016)	0.264<br>(0.220-0.377)	NA	0.006<br>(0.001-0.041)			0.047<br>(0.028-0.064)	1.47<br>(1.27-1.67)	1.91<br>(1.45-2.42)	-25085
	2007		0.005<br>(0.002-0.008)	0.336<br>(0.189-0.878)	NA	0.005<br>(0.001-0.030)			0.019<br>(0.009-0.033)	1.18<br>(1.08-1.32)	1.27<br>(1.10-1.56)	-14719
Anker [18]	1996	Sri Lanka	0.033<br>(0.001-0.102)	0.004<br>(0.002-0.427)	NA	0.320<br>(0.038-0.919)	0.218<br>(0.075-0.539)	0.623<br>(0.049-0.975)	0.133<br>(0.001-0.407)	2.99<br>(1.01-7.17)	4.97<br>(1.01-12.37)	-1139
	1997		0.028<br>(0.001-0.103)	0.002<br>(0.001-0.346)	NA	0.214<br>(0.003-0.929)			0.113<br>(0.001-0.412)	2.41<br>(1.01-6.14)	3.69<br>(1.01-11.46)	-810
	1998		0.035<br>(0.001-0.115)	0.003<br>(0.002-0.093)	NA	0.175<br>(0.002-0.923)			0.138<br>(0.001-0.458)	2.62<br>(1.02-6.14)	3.95<br>(1.03-11.69)	-989
	1999		0.024<br>(0.001-0.092)	0.004<br>(0.002-0.673)	NA	0.227<br>(0.005-0.929)			0.096<br>(0.001-0.369)	2.14<br>(1.01-5.33)	3.17<br>(1.01-9.56)	-1174
	2000		0.022<br>(0.001-0.104)	0.007<br>(0.004-0.665)	NA	0.223<br>(0.004-0.924)			0.090<br>(0.001-0.415)	2.16<br>(1.01-6.60)	3.30<br>(1.10-12.46)	-2302
	2001		0.017<br>(0.001-0.079)	0.012<br>(0.006-0.846)	NA	0.279<br>(0.027-0.942)			0.067<br>(0.001-0.315)	1.92<br>(1.01-5.75)	2.90<br>(1.01-9.55)	-2218
	2002		0.003<br>(0.000-0.056)	0.041<br>(0.006-0.911)	NA	0.130<br>(0.004-0.869)			0.010<br>(0.001-0.222)	1.12<br>(1.01-4.07)	1.16<br>(1.01-6.82)	-2262
	2003		0.002<br>(0.000-0.052)	0.024<br>(0.003-0.794)	NA	0.127<br>(0.006-0.931)			0.009<br>(0.001-0.207)	1.11<br>(1.01-3.92)	1.15<br>(1.01-6.58)	-1215
	2004		0.010<br>(0.001-0.066)	0.017<br>(0.008-0.854)	NA	0.186<br>(0.010-0.923)			0.039<br>(0.001-0.264)	1.48<br>(1.01-4.76)	1.92<br>(1.01-8.21)	-2864
	2005		0.006<br>(0.000-0.069)	0.006<br>(0.002-0.673)	NA	0.163<br>(0.006-0.898)			0.022<br>(0.001-0.275)	1.29<br>(1.01-4.63)	1.49<br>(1.01-7.86)	-881

*Assumption 1: individuals can be infected up to four times. Assumption 2: individuals are immune after two infections.

Table S3 continued (5/5).

Author [Ref]	Year	Country	λ (95% CrI)	ρ (95% CrI)	β (95% CrI)	ψ (95% CrI)	γ_1 (95% CrI)	γ_3	λ_{tot} (95% CrI)	R_0 * (95% CrI)		LnL
										1	2	
Kulatilaka [40]	2010	Sri Lanka	0.066<br>(0.041-0.128)	0.001<br>(0.001-0.001)	0.084<br>(0.030-0.536)	0.009<br>(0.003-0.046)	0.218<br>(0.075-0.539)	0.623<br>(0.049-0.975)	0.264<br>(0.163-0.512)	3.65<br>(2.46-7.17)	7.43<br>(4.38-16.65)	-1565
Limkittikul [41]	2000	Thailand	0.055<br>(0.034-0.209)	0.012<br>(0.003-0.016)	0.060<br>(0.011-0.734)	0.032<br>(0.008-0.208)	0.009<br>(0.001-0.040)	0.527<br>(0.027-0.976)	0.220<br>(0.135-0.838)	3.36<br>(2.32-12.32)	6.41<br>(3.94-24.95)	-29875
	2001		0.050<br>(0.033-0.090)	0.091<br>(0.057-0.121)	0.059<br>(0.011-0.178)	0.032<br>(0.008-0.147)			0.201<br>(0.133-0.361)	3.14<br>(2.31-5.35)	5.91<br>(3.93-10.79)	-225605
	2002		0.049<br>(0.028-0.125)	0.080<br>(0.017-0.104)	0.057<br>(0.013-0.770)	0.035<br>(0.009-0.192)			0.198<br>(0.144-0.500)	3.31<br>(2.17-8.31)	6.53<br>(2.67-17.57)	-188228
	2003		0.047<br>(0.027-0.204)	0.038<br>(0.010-0.058)	0.088<br>(0.012-0.760)	0.044<br>(0.010-0.263)			0.188<br>(0.107-0.818)	3.20<br>(2.11-14.47)	6.30<br>(3.52-30.64)	-107289
	2004		0.044<br>(0.024-0.214)	0.023<br>(0.005-0.036)	0.090<br>(0.011-0.886)	0.043<br>(0.009-0.217)			0.176<br>(0.095-0.857)	3.04<br>(1.97-15.31)	5.92<br>(3.15-32.37)	-66730
	2005		0.042<br>(0.022-0.145)	0.028<br>(0.009-0.043)	0.087<br>(0.010-0.599)	0.035<br>(0.007-0.183)			0.166<br>(0.090-0.579)	2.93<br>(1.91-10.20)	5.65<br>(3.02-21.82)	-79945
	2006		0.041<br>(0.013-0.207)	0.027<br>(0.007-0.044)	0.108<br>(0.011-0.714)	0.050<br>(0.010-0.216)			0.166<br>(0.051-0.829)	2.96<br>(1.48-15.33)	5.74<br>(1.92-32.31)	-80484
	2007		0.040<br>(0.025-0.066)	0.051<br>(0.029-0.066)	0.046<br>(0.006-0.179)	0.029<br>(0.008-0.124)			0.159<br>(0.100-0.263)	2.90<br>(2.07-4.61)	5.61<br>(3.44-9.71)	-112810
	2008		0.037<br>(0.027-0.051)	0.076<br>(0.042-0.090)	0.031<br>(0.006-0.163)	0.015<br>(0.004-0.082)			0.147<br>(0.109-0.205)	2.74<br>(2.20-3.66)	5.20<br>(3.80-7.50)	-159451
	2009		0.036<br>(0.023-0.169)	0.043<br>(0.008-0.059)	0.054<br>(0.007-0.818)	0.022<br>(0.005-0.159)			0.145<br>(0.091-0.678)	2.75<br>(1.98-12.94)	5.24<br>(3.22-26.94)	-103220
	2010		0.034<br>(0.027-0.044)	0.100<br>(0.075-0.119)	0.029<br>(0.005-0.078)	0.009<br>(0.003-0.047)			0.136<br>(0.106-0.176)	2.69<br>(2.24-3.34)	5.11<br>(3.92-6.77)	-215302
Wichmann [34]	2006		0.041<br>(0.017-0.156)	0.369<br>(0.211-0.947)	NA	0.161<br>(0.005-0.889)			0.163<br>(0.069-0.624)	1.36<br>(1.14-3.02)	1.64<br>(1.19-8.60)	-31
	2007		0.068<br>(0.029-0.191)	0.623<br>(0.471-0.963)	NA	0.229<br>(0.017-0.908)			0.272<br>(0.118-0.763)	1.72<br>(1.27-4.04)	2.65<br>(1.45-12.97)	-114
Bangkok^ [26]	2000		0.047<br>(0.038-0.055)	0.055<br>(0.052-0.058)	NA	0.013<br>(0.006-0.029)	0	0	0.188<br>(0.151-0.219)	3.30<br>(2.73-3.84)	7.17<br>(5.49-8.69)	-11467
Ratchaburi^ [26]	2000		0.050<br>(0.043-0.058)	0.108<br>(0.103-0.115)	NA	0.012<br>(0.005-0.033)	0	0	0.202<br>(0.170-0.232)	3.37<br>(2.92-3.82)	6.59<br>(5.51-7.67)	-2810
Rayong [26]	2010		0.025<br>(0.023-0.027)	0.114<br>(0.106-0.123)	NA	0.001<br>(0.001-0.002)	0.009<br>(0.001-0.040)	0.527<br>(0.027-0.976)	0.099<br>(0.092-0.107)	1.99<br>(1.91-2.08)	3.24<br>(3.02-3.49)	-4128
Ghouth [42]	2010	Yemen	0.006<br>(0.001-0.018)	0.163<br>(0.055-0.886)	NA	0.080<br>(0.016-0.451)	0.224<br>(0.006-0.933)	0.491<br>(0.025-0.982)	0.018<br>(0.002-0.054)	1.16<br>(1.02-1.48)	1.18<br>(1.02-1.66)	-1792

* Assumption 1: individuals can be infected up to four times. Assumption 2: individuals are immune after two infections. ^All cases reported = DHF, we have assumed that all cases were due to secondary cases and fixed γ_1 and γ_3 = 0.

Table S4: Summary parameter estimates from model 2 fitted to yearly incidence data (model 2A).

Author [Ref]	Year	Country	λ (95%CrI)	ρ_{young} (95%CrI)	ρ_{old} (95%CrI)	β (95%CrI)	Cutoff (95%CrI)	ψ (95%CrI)	γ_1 (95%CrI)	γ_3 (95%CrI)	λ_{tot} (95%CrI)	R_0 * (95%CrI)		LnL
												1	2	
Cordeiro [32]	1995	Brazil	0.013 (0.005-0.026)	0.260 (0.084-0.947)	0.285 (0.054-0.941)	NA	65 (10-80)	0.008 (0.001-0.049)	0.024 (0.003-0.058)	0.550 (0.038-0.980)	0.027 (0.011-0.051)	1.38 (1.15-1.77)	1.38 (1.15-1.77)	-17854
	1996		0.016 (0.009-0.031)	0.467 (0.171-0.952)	0.382 (0.068-0.923)	NA	65 (15-80)	0.010 (0.002-0.060)			0.032 (0.019-0.061)	1.52 (1.29-2.05)	1.52 (1.23-2.05)	-40383
	1997		0.016 (0.012-0.028)	0.668 (0.275-0.975)	0.515 (0.081-0.931)	NA	65 (10-80)	0.008 (0.002-0.043)			0.032 (0.024-0.055)	1.51 (1.37-1.93)	1.51 (1.37-1.93)	-57709
	1998		0.021 (0.017-0.028)	0.686 (0.388-0.986)	0.837 (0.113-0.990)	NA	20 (15-80)	0.005 (0.001-0.038)			0.041 (0.034-0.055)	1.67 (1.55-1.93)	1.67 (1.55-1.93)	-93277
	1999		0.017 (0.013-0.026)	0.654 (0.290-0.964)	0.488 (0.082-0.924)	NA	65 (10-80)	0.003 (0.001-0.022)			0.035 (0.025-0.052)	1.55 (1.39-1.87)	1.55 (1.39-1.87)	-64111
	2000		0.017 (0.010-0.026)	0.514 (0.190-0.968)	0.458 (0.057-0.938)	NA	65 (15-80)	0.004 (0.001-0.028)			0.033 (0.020-0.052)	1.55 (1.32-1.89)	1.55 (1.32-1.89)	-50580
	2001		0.016 (0.007-0.025)	0.336 (0.190-0.924)	0.352 (0.053-0.935)	NA	65 (5-80)	0.005 (0.001-0.029)			0.031 (0.015-0.051)	1.51 (1.23-1.88)	1.51 (1.23-1.88)	-31021
	2002		0.024 (0.021-0.032)	0.949 (0.752-0.998)	0.683 (0.041-0.993)	NA	80 (10-80)	0.044 (0.015-0.169)			0.071 (0.064-0.095)	1.81 (1.72-2.14)	2.18 (2.03-2.72)	-209223
	2003		0.010 (0.006-0.015)	0.487 (0.275-0.972)	0.413 (0.072-0.956)	NA	65 (5-80)	0.007 (0.001-0.040)			0.031 (0.018-0.046)	1.33 (1.19-1.50)	1.42 (1.22-1.68)	-47533
	2004		0.014 (0.003-0.018)	0.078 (0.057-0.704)	0.376 (0.021-0.972)	NA	80 (5-80)	0.005 (0.001-0.046)			0.041 (0.009-0.055)	1.44 (1.09-1.61)	1.60 (1.10-1.86)	-11581

*Assumption 1: individuals can be infected up to four times. Assumption 2: individuals are immune after two infections.

Table S4 continued (2/8).

Author [Ref]	Year	Country	λ (95%CrI)	ρ_{young} (95%CrI)	ρ_{old} (95%CrI)	B (95%CrI)	Cutoff (95%CrI)	ψ (95%CrI)	γ_1 (95%CrI)	γ_3 (95%CrI)	λ_{tot} (95%CrI)	R_0 * (95%CrI)		LnL
												1	2	
Cordeiro [32]	2005	Brazil	0.012 (0.004-0.018)	0.182 (0.116-0.767)	0.302 (0.035-0.965)	NA	80 (5-80)	0.007 (0.002-0.041)	0.024 (0.003-0.058)	0.550 (0.038-0.980)	0.035 (0.013-0.053)	1.38 (1.13-1.58)	1.50 (1.16-1.82)	-23548
	2006		0.014 (0.005-0.022)	0.220 (0.129-0.892)	0.281 (0.046-0.947)	NA	80 (5-80)	0.010 (0.003-0.056)			0.041 (0.015-0.066)	1.45 (1.15-1.76)	1.61 (1.18-2.09)	-34400
Penna [12]	2001		0.007 (0.003-0.081)	0.265 (0.009-0.910)	0.466 (0.066-0.976)	0.022 (0.001-0.875)	70 (15-90)	0.049 (0.002-0.671)			0.027 (0.010-0.324)	1.19 (1.07-3.95)	1.29 (1.09-7.47)	-14641
	2002		0.008 (0.002-0.076)	0.113 (0.009-0.879)	0.370 (0.043-0.938)	0.053 (0.000-0.946)	70 (15-90)	0.093 (0.003-0.851)			0.030 (0.009-0.304)	1.21 (1.06-3.74)	1.34 (1.08-7.01)	-9048
	2003		0.009 (0.001-0.074)	0.096 (0.014-0.816)	0.395 (0.060-0.948)	0.110 (0.001-0.962)	70 (15-90)	0.127 (0.004-0.748)			0.037 (0.005-0.296)	1.26 (1.03-3.66)	1.43 (1.04-6.83)	-10374
	2004		0.006 (0.002-0.076)	0.124 (0.006-0.787)	0.310 (0.033-0.954)	0.018 (0.001-0.874)	70 (15-90)	0.065 (0.004-0.787)			0.024 (0.007-0.304)	1.17 (1.05-3.74)	1.25 (1.06-7.01)	-5541
	2005		0.005 (0.002-0.077)	0.251 (0.017-0.870)	0.435 (0.054-0.960)	0.009 (0.001-0.681)	70 (15-90)	0.045 (0.004-0.510)			0.021 (0.008-0.311)	1.15 (1.05-3.78)	1.26 (1.06-7.10)	-8558
	2006		0.005 (0.002-0.052)	0.231 (0.013-0.749)	0.383 (0.047-0.944)	0.009 (0.001-0.465)	70 (15-90)	0.031 (0.001-0.459)			0.021 (0.009-0.207)	1.15 (1.06-2.76)	1.22 (1.08-4.83)	-7825
Vong [33]	2006	Cambodia	0.077 (0.056-0.127)	0.190 (0.123-0.220)	0.099 (0.025-0.797)	NA	16 (6-21)	0.011 (0.004-0.027)	0.128 (0.068-0.279)	0.508 (0.030-0.977)	0.310 (0.224-0.506)	2.15 (1.77-3.19)	3.83 (2.72-7.02)	-20853
	2007		0.069 (0.056-0.078)	0.957 (0.759-0.999)	0.312 (0.044-0.947)	NA	20 (6-21)	0.004 (0.002-0.010)			0.275 (0.223-0.318)	1.99 (1.77-2.19)	3.35 (2.71-3.94)	-110702
	2008		0.046 (0.032-0.059)	0.345 (0.282-0.420)	0.042 (0.013-0.128)	NA	18 (17-18)	0.005 (0.002-0.012)			0.183 (0.127-0.234)	1.61 (1.40-1.81)	2.27 (1.73-2.82)	-34269

*Assumption 1: individuals can be infected up to four times. Assumption 2: individuals are immune after two infections.

Table S4 continued (3/8).

Author [Ref]	Year	Country	λ (95%CrI)	ρ_{young} (95%CrI)	ρ_{old} (95%CrI)	B (95%CrI)	Cutoff (95%CrI)	ψ (95%CrI)	γ_1 (95%CrI)	γ_3 (95%CrI)	λ_{tot} (95%CrI)	R_0 * (95%CrI)		LnL
												1	2	
Wichmann [34]	2006	Cambodia	0.043 (0.014-0.238)	0.310 (0.140-0.927)	0.558 (0.044-0.976)	NA	15 (5-15)	0.227 (0.010-0.929)	0.265 (0.062-0.705)	0.589 (0.046-0.981)	0.171 (0.056-0.952)	1.38 (1.11-5.02)	1.69 (1.15-19.78)	-31
	2007		0.078 (0.050-0.187)	0.882 (0.630-0.993)	0.626 (0.021-0.985)	NA	15 (5-15)	0.157 (0.002-0.884)			0.313 (0.201-0.747)	1.73 (1.44-3.12)	2.58 (1.83-6.16)	-543
Anker [18]	2000	Laos	0.001 (0.000-0.150)	0.410 (0.002-0.947)	0.291 (0.026-0.949)	NA	15 (5-15)	0.217 (0.010-0.913)	0.070 (0.003-0.563)	0.537 (0.033-0.979)	0.005 (0.001-0.600)	1.05 (1.01-6.06)	1.06 (1.01-10.06)	-1052
	2001		0.003 (0.001-0.166)	0.300 (0.006-0.956)	0.277 (0.041-0.964)	NA	15 (5-15)	0.238 (0.008-0.918)			0.014 (0.004-0.663)	1.12 (1.03-6.36)	1.18 (1.04-13.31)	-3631
	2002		0.017 (0.002-0.127)	0.120 (0.019-0.937)	0.251 (0.072-0.922)	NA	15 (5-15)	0.235 (0.007-0.933)			0.070 (0.008-0.510)	1.74 (1.08-5.54)	2.36 (1.11-9.44)	-8186
	2003		0.021 (0.003-0.121)	0.162 (0.040-0.959)	0.405 (0.112-0.971)	NA	15 (5-15)	0.238 (0.010-0.931)			0.085 (0.011-0.486)	1.81 (1.11-5.20)	2.43 (1.15-9.94)	-17292
	2004		0.006 (0.001-0.176)	0.196 (0.007-0.957)	0.241 (0.030-0.904)	NA	15 (5-15)	0.225 (0.005-0.928)			0.022 (0.003-0.706)	1.19 (1.02-6.53)	1.30 (1.02-14.35)	-3281
	2005		0.045 (0.001-0.179)	0.026 (0.011-0.969)	0.309 (0.050-0.961)	NA	15 (5-15)	0.230 (0.009-0.918)			0.181 (0.005-0.714)	2.36 (1.04-6.17)	3.31 (1.05-13.61)	-5243
	2006		0.034 (0.001-0.169)	0.032 (0.011-0.950)	0.310 (0.050-0.944)	NA	15 (5-15)	0.219 (0.010-0.923)			0.137 (0.005-0.677)	2.20 (1.05-6.51)	3.05 (1.06-14.10)	-5948
Khampa-pongpane [43]	2010		0.020 (0.002-0.161)	0.083 (0.017-0.712)	0.240 (0.046-0.925)	0.114 (0.003-0.869)	40 (5-40)	0.090 (0.010-0.615)			0.080 (0.006-0.644)	1.58 (1.04-6.49)	2.06 (1.05-12.46)	-33953
Prasith [44]	2010		0.041 (0.002-0.178)	0.066 (0.007-0.586)	0.364 (0.041-0.952)	0.391 (0.016-0.965)	80 (5-80)	0.365 (0.034-0.942)			0.161 (0.007-0.712)	2.42 (1.05-7.58)	3.96 (1.06-13.03)	-22762

*Assumption 1: individuals can be infected up to four times. Assumption 2: individuals are immune after two infections.

Table S4 continued (4/8)

Author [Ref]	Year	Country	λ (95%CrI)	ρ_{young} (95%CrI)	ρ_{old} (95%CrI)	β (95%CrI)	Cutoff (95%CrI)	ψ (95%CrI)	γ_1 (95%CrI)	γ_3 (95%CrI)	λ_{tot} (95%CrI)	R_0 * (95%CrI)		LnL
												1	2	
Anker [18]	1998	Philippines	0.004 (0.001-0.118)	0.369 (0.007-0.970)	0.130 (0.017-0.879)	NA	15 (5-15)	0.284 (0.007-0.924)	0.070 (0.003-0.563)	0.537 (0.033-0.979)	0.014 (0.004-0.472)	1.13 (1.04-4.78)	1.19 (1.05-8.67)	-33864
	1999		0.068 (0.001-0.147)	0.004 (0.001-0.939)	0.147 (0.006-0.857)	NA	15 (5-15)	0.361 (0.014-0.940)			0.271 (0.001-0.588)	3.43 (1.01-6.02)	5.34 (1.01-11.83)	-9157
	2001		0.002 (0.001-0.004)	0.644 (0.167-0.981)	0.102 (0.019-0.671)	NA	15 (5-15)	0.239 (0.008-0.897)			0.007 (0.002-0.017)	1.07 (1.02-1.17)	1.10 (1.02-1.25)	-23843
	2002		0.001 (0.001-0.109)	0.610 (0.005-0.975)	0.148 (0.010-0.818)	NA	15 (5-15)	0.337 (0.033-0.936)			0.006 (0.002-0.436)	1.06 (1.02-4.72)	1.07 (1.02-7.58)	-16213
	2003		0.018 (0.002-0.134)	0.050 (0.004-0.927)	0.044 (0.008-0.768)	NA	15 (5-15)	0.374 (0.034-0.936)			0.073 (0.007-0.535)	1.83 (1.07-6.31)	2.58 (1.09-10.72)	-28567
	2004		0.002 (0.001-0.006)	0.536 (0.104-0.980)	0.086 (0.012-0.802)	NA	15 (5-15)	0.250 (0.010-0.926)			0.007 (0.003-0.022)	1.07 (1.02-1.22)	1.09 (1.03-1.35)	-22019
	2005		0.080 (0.017-0.142)	0.010 (0.005-0.033)	0.182 (0.020-0.898)	NA	15 (15-15)	0.391 (0.027-0.967)			0.319 (0.068-0.568)	3.60 (1.62-5.49)	5.78 (2.10-10.62)	-32194
Rigau-Perez [35]	1994	Puerto Rico	0.021 (0.012-0.029)	0.459 (0.283-0.927)	0.472 (0.175-0.956)	NA	55 (20-99)	0.003 (0.001-0.017)	0.413 (0.110-0.884)	0.637 (0.067-0.985)	0.063 (0.037-0.086)	1.84 (1.47-2.22)	2.24 (1.64-2.86)	-50387
	1995		0.019 (0.001-0.028)	0.041 (0.026-0.747)	0.378 (0.020-0.972)	NA	85 (30-85)	0.008 (0.001-0.181)			0.058 (0.004-0.084)	1.71 (1.04-2.09)	2.03 (1.05-2.64)	-3062
	1996		0.019 (0.010-0.026)	0.037 (0.025-0.801)	0.379 (0.026-0.959)	NA	85 (30-85)	0.011 (0.001-0.296)			0.056 (0.004-0.076)	1.68 (1.04-1.96)	1.98 (1.04-2.43)	-2697
	1997		0.020 (0.001-0.025)	0.042 (0.027-0.829)	0.340 (0.019-0.977)	NA	85 (30-85)	0.009 (0.001-0.214)			0.061 (0.006-0.088)	1.76 (1.06-2.15)	2.10 (1.07-2.73)	-3371

*Assumption 1: individuals can be infected up to four times. Assumption 2: individuals are immune after two infections.

Table S4 continued (5/8)

Author [Ref]	Year	Country	λ (95%CrI)	ρ_{young} (95%CrI)	ρ_{old} (95%CrI)	β (95%CrI)	Cutoff (95%CrI)	ψ (95%CrI)	γ_1 (95%CrI)	γ_3 (95%CrI)	λ_{tot} (95%CrI)	R_0 * (95%CrI)		LnL
												1	2	
Ramos [36]	2006	Puerto Rico	0.017 (0.006-0.043)	0.443 (0.179-0.928)	0.470 (0.078-0.959)	NA	40 (00-61)	0.038 (0.001-0.450)	0.413 (0.110-0.884)	0.637 (0.067-0.985)	0.069 (0.023-0.171)	1.63 (1.19-2.93)	2.30 (1.28-6.22)	-203
Sharp [37]	2010		0.015 (0.003-0.030)	0.199 (0.108-0.944)	0.276 (0.114-0.925)	NA	50 (150-81)	0.020 (0.004-0.076)			0.059 (0.010-0.119)	1.67 (1.10-2.55)	2.39 (1.13-4.68)	-24289
Tomashek [38]	2007		0.010 (0.001-0.023)	0.066 (0.035-0.936)	0.240 (0.044-0.947)	NA	70 (15-85)	0.023 (0.007-0.075)			0.040 (0.002-0.090)	1.39 (1.02-1.97)	1.70 (1.02-3.10)	-7042
Anker [18]	1999	Singapore	0.003 (0.001-0.126)	0.321 (0.004-0.951)	0.313 (0.039-0.934)	NA	15 (5-15)	0.217 (0.005-0.948)	0.053 (0.007-0.159)	0.549 (0.027-0.976)	0.010 (0.004-0.505)	1.11 (1.04-7.44)	1.16 (1.04-14.84)	-521
	2000		0.002 (0.001-0.162)	0.351 (0.002-0.975)	0.171 (0.017-0.919)	NA	15 (5-15)	0.264 (0.019-0.943)			0.010 (0.002-0.647)	1.13 (1.03-12.07)	1.18 (1.03-23.43)	-362
	2001		0.011 (0.001-0.123)	0.136 (0.008-0.947)	0.167 (0.033-0.940)	NA	15 (5-15)	0.320 (0.16-0.958)			0.044 (0.005-0.491)	1.64 (1.06-11.61)	2.31 (1.07-22.52)	-940
	2002		0.007 (0.002-0.103)	0.241 (0.010-0.961)	0.280 (0.065-0.938)	NA	15 (5-15)	0.280 (0.015-0.956)			0.029 (0.007-0.410)	1.36 (1.08-7.86)	1.65 (1.10-15.94)	-1506
	2003		0.013 (0.002-0.106)	0.133 (0.011-0.946)	0.275 (0.076-0.961)	NA	15 (5-15)	0.307 (0.013-0.955)			0.053 (0.007-0.424)	1.72 (1.08-8.08)	2.45 (1.11-16.38)	-1859
	2004		0.015 (0.003-0.078)	0.228 (0.031-0.946)	0.441 (0.169-0.952)	NA	15 (5-15)	0.305 (0.010-0.959)			0.061 (0.014-0.313)	1.77 (1.16-5.20)	2.49 (1.23-9.34)	-4135
	2005		0.024 (0.004-0.084)	0.227 (0.046-0.943)	0.367 (0.149-0.942)	NA	15 (5-15)	0.316 (0.019-0.950)			0.097 (0.018-0.336)	2.51 (1.22-7.29)	4.30 (1.36-14.45)	-6857

*Assumption 1: individuals can be infected up to four times. Assumption 2: individuals are immune after two infections.

Table S4 continued (6/8).

Author [Ref]	Year	Country	λ (95%CrI)	ρ_{young} (95%CrI)	ρ_{old} (95%CrI)	B (95%CrI)	Cutoff (95%CrI)	ψ (95%CrI)	γ_1 (95%CrI)	γ_3 (95%CrI)	λ_{tot} (95%CrI)	R_0 * (95%CrI)		LnL
												**1**	**2**	
Koh [39]	2005	Singapore	0.011 (0.005-0.016)	0.265 (0.191-0.777)	0.361 (0.089-0.945)	NA	65 (25-85)	0.007 (0.002-0.036)	0.053 (0.007-0.159)	0.549 (0.027-0.976)	0.043 (0.021-0.065)	1.43 (1.19-1.67)	1.80 (1.29-2.41)	-20559
Ler [23]	2005		0.012 (0.006-0.017)	0.267 (0.199-0.649)	0.336 (0.028-0.955)	NA	81 (15-81)	0.007 (0.001-0.053)			0.048 (0.025-0.067)	1.48 (1.23-1.72)	1.92 (1.37-2.54)	-25085
	2007		0.004 (0.002-0.009)	0.382 (0.169-0.941)	0.458 (0.051-0.958)	NA	81 (15-81)	0.004 (0.001-0.031)			0.018 (0.009-0.034)	1.17 (1.08-1.34)	1.24 (1.10-1.59)	-14719
Anker [18]	1996	Sri Lanka	0.001 (0.001-0.028)	0.352 (0.001-0.970)	0.094 (0.004-0.854)	NA	15 (55-15)	0.358 (0.034-0.947)	0.218 (0.075-0.539)	0.623 (0.049-0.975)	0.004 (0.001-0.112)	1.05 (1.01-2.65)	1.06 (1.01-4.35)	-1139
	1997		0.001 (0.000-0.192)	0.603 (0.001-0.979)	0.122 (0.008-0.720)	NA	15 (55-15)	0.210 (0.004-0.907)			0.002 (0.001-0.768)	1.02 (1.01-10.89)	1.02 (1.01-24.78)	-810
	1998		0.024 (0.000-0.043)	0.381 (0.001-0.961)	0.147 (0.004-0.846)	NA	15 (5-15)	0.359 (0.005-0.947			0.096 (0.001-0.172)	2.11 (1.01-3.01)	3.08 (1.01-4.64)	-990
	1999		0.001 (0.000-0.076)	0.333 (0.001-0.955)	0.165 (0.007-0.913)	NA	15 (5-15)	0.281 (0.005-0.936)			0.005 (0.001-0.304)	1.05 (1.01-4.59)	1.06 (1.01-7.86)	-1175
	2000		0.016 (0.001-0.107)	0.020 (0.002-0.968)	0.185 (0.008-0.926)	NA	15 (5-15)	0.312 (0.006-0.943)			0.063 (0.001-0.429)	1.79 (1.01-6.80)	2.56 (1.01-12.95)	-2302
	2001		0.004 (0.001-0.246)	0.134 (0.002-0.956)	0.170 (0.011-0.918)	NA	15 (5-15)	0.345 (0.026-0.936)			0.017 (0.001-0.982)	1.21 (1.01-14.83)	1.32 (1.02-32.20)	-2218
	2002		0.002 (0.001-0.234)	0.399 (0.002-0.973)	0.270 (0.015-0.936)	NA	15 (5-15)	0.198 (0.009-0.935)			0.004 (0.001-0.936)	1.05 (1.01-14.64)	1.06 (1.01-35.58)	-2263

*Assumption 1: individuals can be infected up to four times. Assumption 2: individuals are immune after two infections.

Table S4 continued (7/8).

Author [Ref]	Year	Country	λ (95%CrI)	ρ_{young} (95%CrI)	ρ_{old} (95%CrI)	B (95%CrI)	Cutoff (95%CrI)	ψ (95%CrI)	γ_1 (95%CrI)	γ_3 (95%CrI)	λ_{tot} (95%CrI)	R_0 *(95%CrI)		LnL
												1	2	
Anker [18]	2003	Sri Lanka	0.001 (0.000-0.213)	0.530 (0.001-0.977)	0.260 (0.015-0.884)	NA	15 (5-15)	0.181 (0.008-0.909)	0.218 (0.075-0.539)	0.623 (0.049-0.975)	0.002 (0.001-0.852)	1.03 (1.01-13.55)	1.03 (1.01-31.5)	-1215
	2004		0.003 (0.001-0.246)	0.156 (0.002-0.961)	0.242 (0.015-0.953)	NA	15 (5-15)	0.301 (0.009-0.957)			0.010 (0.001-0.983)	1.13 (1.01-15.83)	1.18 (1.02-38.87)	-2865
	2005		0.001 (0.000-0.086)	0.496 (0.001-0.958)	0.246 (0.013-0.891)	NA	15 (5-15)	0.166 (0.003-0.918)			0.002 (0.001-0.344)	1.02 (1.01-5.52)	1.02 (1.01-9.75)	-882
Kulatilaka [40]	2010		0.066 (0.040-0.128)	0.001 (0.001-0.002)	0.485 (0.002-0.979)	0.077 (0.029-0.445)	65 (55-65)	0.010 (0.003-0.057)			0.264 (0.159-0.512)	3.65 (2.41-7.17)	7.43 (4.26-16.65)	-1566
Limkittikul [41]	2000	Thailand	0.068 (0.030-0.189)	0.007 (0.002-0.016)	0.457 (0.010-0.974)	0.220 (0.012-0.901)	80 (55-80)	0.075 (0.010-0.310)	0.009 (0.001-0.040)	0.527 (0.027-0.976)	0.270 (0.120-0.756)	4.02 (2.15-11.10)	7.90 (3.54-22.5)	-29877
	2001		0.049 (0.033-0.079)	0.099 (0.068-0.123)	0.354 (0.030-0.960)	0.043 (0.004-0.117)	80 (35-80)	0.027 (0.008-0.133)			0.197 (0.132-0.315)	3.09 (2.30-4.69)	5.80 (3.92-9.37)	-225604
	2002		0.055 (0.019-0.225)	0.055 (0.026-0.095)	0.364 (0.014-0.963)	0.157 (0.023-0.447)	80 (25-80)	0.069 (0.014-0.275)			0.220 (0.076-0.900)	3.63 (1.73-15.55)	7.30 (2.54-39.49)	-188228
	2003		0.049 (0.028-0.208)	0.037 (0.008-0.060)	0.410 (0.022-0.970)	0.098 (0.006-0.927)	80 (45-80)	0.052 (0.009-0.263)			0.196 (0.113-0.832)	3.32 (2.17-14.74)	6.59 (3.69-27.66)	-107291
	2004		0.041 (0.021-0.084)	0.030 (0.013-0.067)	0.376 (0.017-0.958)	0.038 (0.002-0.290)	80 (15-80)	0.025 (0.007-0.129)			0.164 (0.083-0.335)	2.87 (1.82-5.60)	5.47 (2.77-11.87)	-66728
	2005		0.038 (0.027-0.058)	0.037 (0.023-0.045)	0.361 (0.021-0.970)	0.035 (0.002-0.132)	80 (35-80)	0.021 (0.007-0.101)			0.154 (0.109-0.234)	2.75 (2.15-3.97)	5.20 (3.65-8.18)	-79944

*Assumption 1: individuals can be infected up to four times. Assumption 2: individuals are immune after two infections.

Table S4 continued (8/8).

Author [Ref]	Year	Country	λ (95%CrI)	ρ_{young} (95%CrI)	ρ_{old} (95%CrI)	β (95%CrI)	Cutoff (95%CrI)	ψ (95%CrI)	γ_1 (95%CrI)	γ_3 (95%CrI)	λ_{tot} (95%CrI)	R_0 (95%CrI)*		LnL
												1	2	
Limkittikul [41]	2006	Thailand	0.040 (0.023-0.132)	0.033 (0.012-0.046)	0.408 (0.017-0.966)	0.066 (0.004-0.396)	80 (35-80)	0.041 (0.009-0.193)	0.009 (0.001-0.040)	0.527 (0.027-0.976)	0.159 (0.092-0.528)	2.86 (1.95-9.41)	5.48 (3.13-20.1)	-80483
	2007		0.039 (0.026-0.062)	0.052 (0.031-0.067)	0.345 (0.021-0.956)	0.043 (0.003-0.146)	80 (25-80)	0.028 (0.008-0.127)			0.157 (0.105-0.249)	2.87 (2.14-4.37)	5.52 (3.64-9.15)	-112810
	2008		0.036 (0.028-0.050)	0.077 (0.055-0.092)	0.355 (0.027-0.969)	0.029 (0.003-0.095)	80 (35-80)	0.014 (0.004-0.080)			0.145 (0.113-0.198)	2.72 (2.26-3.55)	5.16 (3.96-7.22)	-159451
	2009		0.035 (0.025-0.054)	0.047 (0.026-0.059)	0.318 (0.024-0.966)	0.037 (0.004-0.165)	80 (15-80)	0.017 (0.005-0.089)			0.140 (0.101-0.217)	2.67 (2.13-3.92)	5.05 (3.60-8.12)	-103220
	2010		0.034 (0.027-0.044)	0.101 (0.077-0.118)	0.348 (0.034-0.961)	0.028 (0.004-0.074)	80 (35-80)	0.009 (0.003-0.039)			0.136 (0.107-0.174)	2.68 (2.25-3.30)	5.10 (3.95-6.69)	-215303
Wichmann [34]	2006		0.046 (0.018-0.156)	0.331 (0.155-0.921)	0.562 (0.040-0.981)	NA	10 (5-15)	0.199 (0.005-0.865)			0.184 (0.072-0.622)	1.41 (1.15-3.01)	1.77 (1.20-8.56)	-31
	2007		0.073 (0.029-0.177)	0.603 (0.360-0.964)	0.600 (0.032-0.984)	NA	15 (5-15)	0.241 (0.013-0.904)			0.292 (0.116-0.707)	1.79 (1.29-3.70)	2.87 (1.44-11.33)	-114
Bangkok^ [26]	2000		0.047 (0.038-0.055)	0.055 (0.052-0.058)	0.508 (0.020-0.974)	NA	98 (98-98)	0.013 (0.006-0.032)	0	0	0.188 (0.151-0.220)	3.30 (2.73-3.85)	7.16 (5.47-8.74)	-11467
Ratchaburi^ [26]	2000		0.051 (0.042-0.058)	0.109 (0.103-0.115)	0.502 (0.024-0.972)	NA	98 (98-98)	0.012 (0.006-0.033)	0	0	0.202 (0.169-0.231)	3.38 (2.91-3.81)	6.61 (5.47-7.63)	-2810
Rayong [26]	2010		0.019 (0.016-0.022)	0.155 (0.134-0.187)	0.074 (0.062-0.088)	NA	28 (27-31)	0.000 (0.000-0.001)	0.009 (0.001-0.040)	0.527 (0.027-0.976)	0.077 (0.064-0.089)	1.73 (1.60-1.87)	2.56 (2.22-2.93)	-4115
Ghouth [42]	2010	Yemen	0.005 (0.001-0.019)	0.199 (0.052-0.959)	0.405 (0.033-0.968)	NA	80 (15-80)	0.093 (0.016-0.463)	0.224 (0.006-0.933)	0.491 (0.025-0.982)	0.015 (0.002-0.056)	1.13 (1.02-1.50)	1.15 (1.02-1.68)	-1792

*Assumption1 = 4 infection, assumption 2 = 2 infections. ^All cases reported = DHF, we have assumed that all cases were due to secondary cases and fixed γ_1 and γ_3 = 0.

Figures S4 and S5 show the distribution of the total force of infection (λ_{total}) grouped by country (calculated by multiplying the serotype-specific force of infection by the number of serotypes in circulation) from models 1B (single reporting rate, ρ , fitted to cumulative incidence data) and 2B (age-dependent reporting rate, ρ_y and ρ_o , fitted to cumulative incidence data) respectively. Individual estimates are given in Table S5 and Table S6.

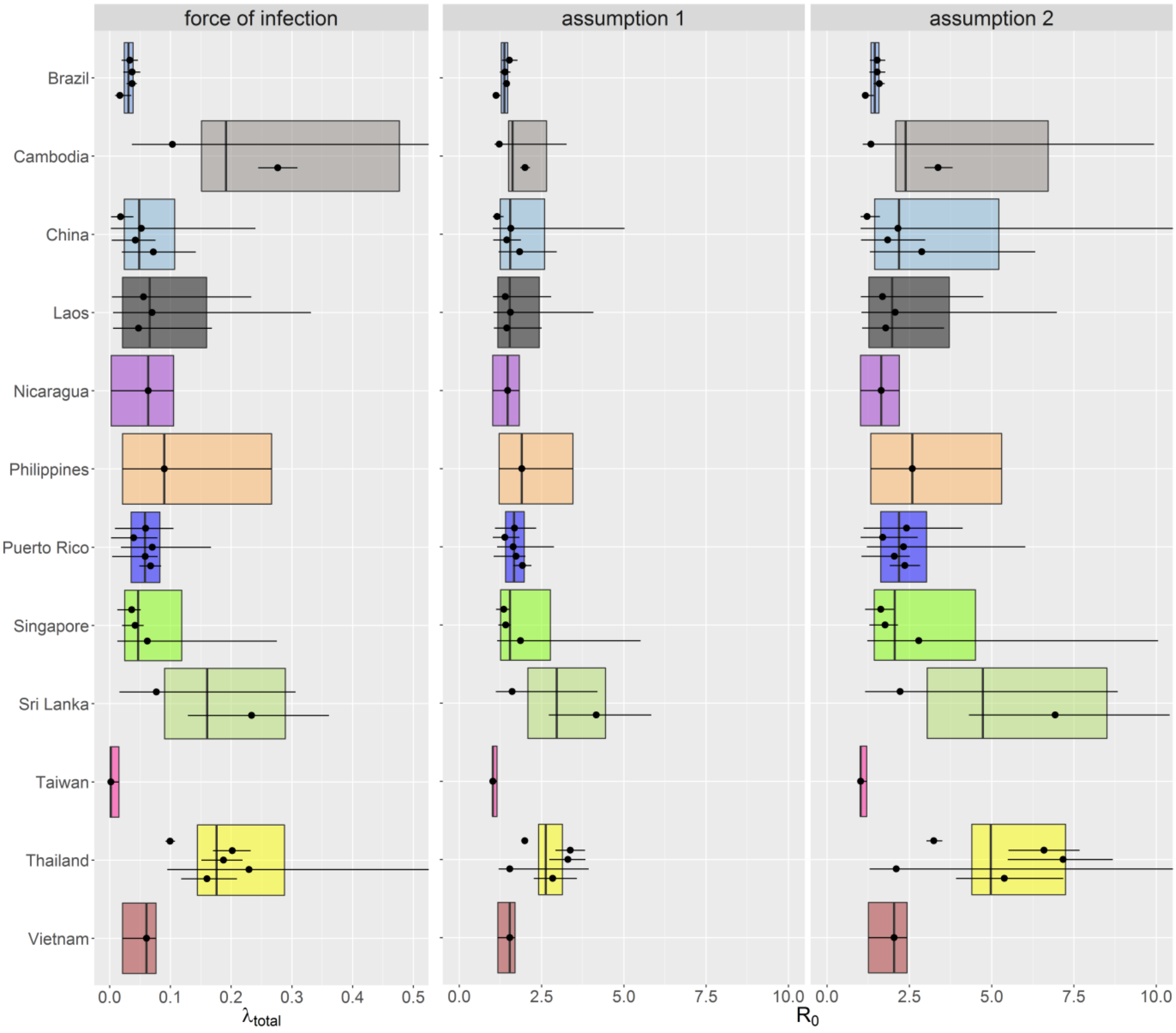

Figure S4: Total force of infection and corresponding R_0 estimates from model 1B (single reporting rate) and fitted to cumulative incidence data grouped by country. Each point represents the posterior median estimate and the error bars the 95% CrI. The box represents the country-specific central estimate calculated by taking the mean values of the MCMC output for each country (the line and limits of the box represents the posterior median and the 95% CrI respectively).

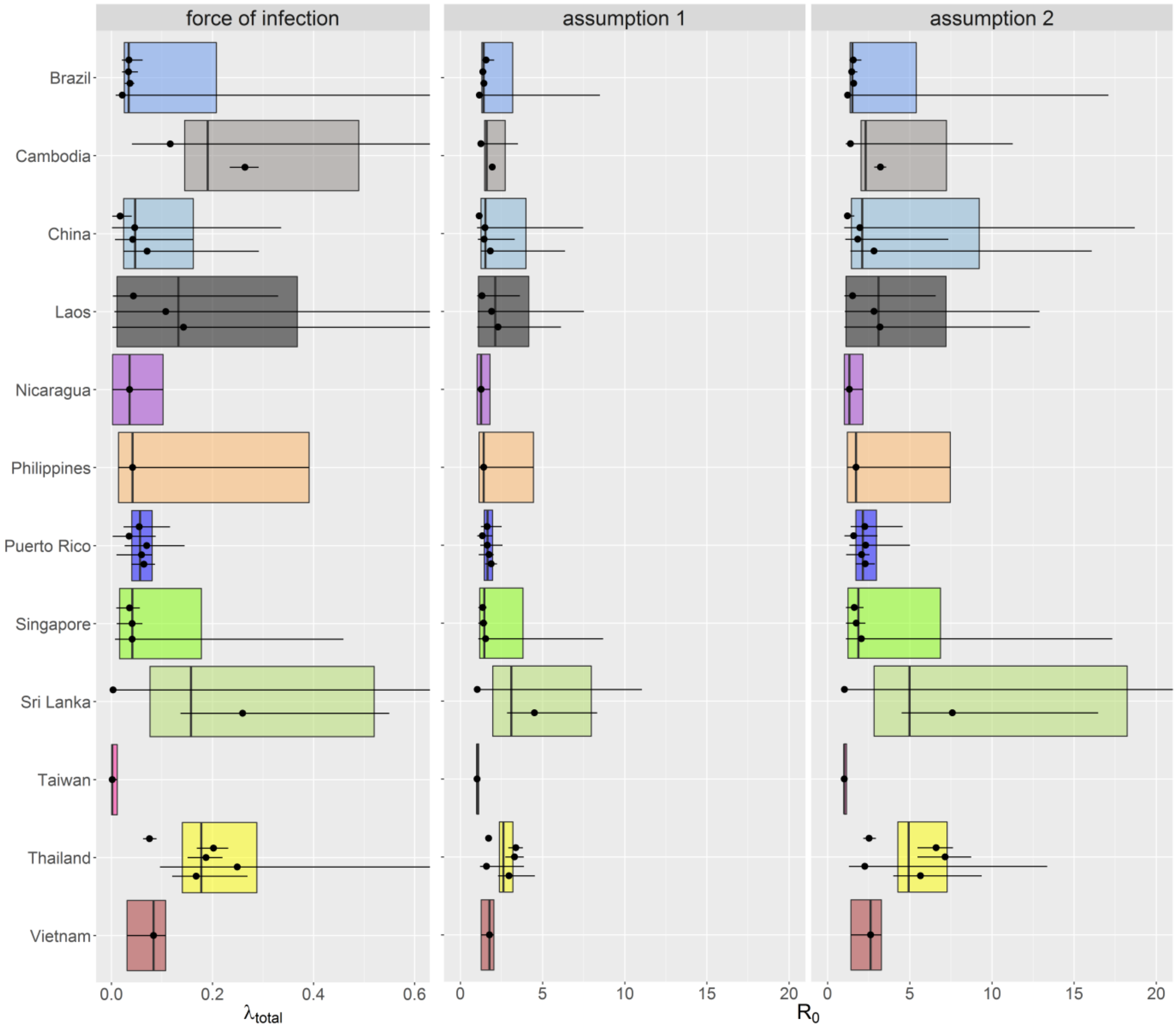

Figure S5: Total force of infection and corresponding R_0 estimates from model 2B (age-dependent reporting rate) and fitted to cumulative incidence data grouped by country. Each point represents the posterior median estimate and the error bars the 95% CrI. The box represents the country-specific central estimate calculated by taking the mean values of the MCMC output for each country (the line and limits of the box represents the posterior median and the 95% CrI respectively).

Table S5: Summary parameter estimates from model 1 fitted to cumulative incidence data where available by country (single reporting rate, model 1B).

Author [Ref]	Year	Country	λ (95% CrI)	ρ (95%CrI)	B (95%CrI)	φ (95%CrI)	γ_1 (95%CrI)	γ_3 (95%CrI)	λ_{tot} (95%CrI)	R_0 (95%CrI)*		LnL
										1	2	
Cordeiro [32]	1995/01	Brazil	0.017 (0.010-0.023)	0.552 (0.377-0.958)	NA	0.008 (0.002-0.040)	0.033 (0.003-0.119)	0.494 (0.039-0.977)	0.033 (0.020-0.047)	1.52 (1.30-1.77)	1.52 (1.30-1.77)	-355182
	2002/06		0.012 (0.008-0.017)	0.485 (0.356-0.830)	NA	0.007 (0.002-0.036)			0.037 (0.023-0.051)	1.40 (1.24-1.56)	1.53 (1.29-1.77)	-326485
Cardoso [16]	2000/09		0.012 (0.009-0.015)	0.078 (0.064-0.104)	NA	0.002 (0.001-0.008)			0.037 (0.028-0.045)	1.44 (1.32-1.54)	1.59 (1.42-1.75)	-4333
Penna [12]	2001/06		0.004 (0.002-0.009)	0.428 (0.158-0.929)	0.005 (0.001-0.035)	0.023 (0.003-0.177)			0.017 (0.009-0.036)	1.12 (1.06-1.26)	1.16 (1.08-1.42)	-55909
Vong [33]	2006/08	Cambodia	0.069 (0.061-0.077)	0.179 (0.147-0.189)	NA	0.003 (0.002-0.007)	0.041 (0.002-0.239)	0.378 (0.015-0.967)	0.277 (0.245-0.309)	2.00 (1.86-2.15)	3.37 (2.96-3.82)	-55933
Wichmann [34]	2006/07		0.026 (0.009-0.168)	0.267 (0.103-0.946)	NA	0.167 (0.005-0.906)			0.104 (0.037-0.671)	1.22 (1.07-3.26)	1.33 (1.09-9.94)	-31
Luo [45]	1978/88	China	0.018 (0.005-0.035)	0.000 (0.000-0.001)	0.416 (0.065-0.745)	0.002 (0.000-0.027)	0.043 (0.003-0.312)	0.505 (0.033-0.975)	0.072 (0.0020-0.142)	1.83 (1.20-2.96)	2.88 (1.30-6.32)	-12911
	1989/99		0.011 (0.001-0.019)	0.000 (0.000-0.001)	0.049 (0.002-0.642)	0.002 (0.000-0.025)			0.042 (0.003-0.076)	1.45 (1.03-1.87)	1.84 (1.04-2.98)	-12430
	2000/09		0.013 (0.001-0.060)	0.000 (0.000-0.001)	0.263 (0.016-0.561)	0.057 (0.016-0.267)			0.052 (0.002-0.240)	1.57 (1.02-5.02)	2.16 (1.02-12.31)	-4413
Guo [46]	2005/11		0.004 (0.001-0.010)	0.000 (0.000-0.005)	0.006 (0.000-0.082)	0.027 (0.009-0.110)			0.018 (0.003-0.039)	1.15 (1.02-1.35)	1.22 (1.02-1.61)	-3459
Anker[18]	2000/06	Laos	0.012 (0.001-0.042)	0.095 (0.040-0.359)	NA	0.191 (0.005-0.921)	0.182 (0.008-0.910)	0.532 (0.028-0.974)	0.048 (0.006-0.168)	1.45 (1.05-2.50)	1.78 (1.06-3.55)	-45133
Khampapongpane [43]	2010		0.014 (0.001-0.058)	0.150 (0.025-0.707)	0.065 (0.002-0.871)	0.067 (0.007-0.376)			0.063 (0.004-0.233)	1.40 (1.03-2.79)	1.69 (1.03-4.75)	-33953
Prasith [44]	2010		0.017 (0.001-0.083)	0.143 (0.049-0.401)	0.158 (0.005-0.903)	0.293 (0.051-0.950)			0.070 (0.006-0.331)	1.56 (1.04-4.08)	2.07 (1.05-6.98)	-22762
Hammond [20]	1999/01	Nicaragua	0.021 (0.001-0.035)	0.026 (0.014-0.802)	NA	0.014 (0.004-0.059)	0.482 (0.032-0.968)	0.505 (0.025-0.971)	0.064 (0.002-0.105)	1.47 (1.02-1.82)	1.64 (1.02-2.19)	-1182
Anker [18]	1998-2005	Philippines	0.023 (0.005-0.067)	0.103 (0.049-0.268)	NA	0.241 (0.019-0.939)	0.365 (0.025-0.948)	0.455 (0.025-0.968)	0.090 (0.021-0.267)	1.90 (1.21-3.45)	2.59 (1.33-5.30)	-166118

*Assumption 1: individuals can be infected up to four times. Assumption 2: individuals are immune after two infections.

Table S5 continued (2/2)

Author [Ref]	Year	Country	λ (95%CrI)	ρ (95%CrI)	β (95%CrI)	φ (95%CrI)	γ_1 (95%CrI)	γ_3 (95%CrI)	λ_{tot} (95%CrI)	R_0 (95%CrI)*		LnL
										1	2	
Rigau-Perez [35]	1994	Puerto Rico	0.022 (0.016-0.028)	0.346 (0.234-0.485)	NA	0.005 (0.023-0.019)	0.417 (0.112-0.914)	0.664 (0.052-0.984)	0.067 (0.049-0.085)	1.92 (1.64-2.20)	2.36 (1.91-2.82)	-50388
	1995/97		0.020 (0.001-0.026)	0.032 (0.021-0.587)	NA	0.007 (0.001-0.178)			0.059 (0.004-0.079)	1.72 (1.05-2.01)	2.04 (1.05-2.51)	-9112
Ramos [36]	2006		0.018 (0.005-0.042)	0.359 (0.203-0.804)	NA	0.046 (0.002-0.410)			0.070 (0.019-0.167)	1.64 (1.15-2.88)	2.33 (1.21-6.03)	-203
Sharp [37]	2010		0.015 (0.002-0.026)	0.161 (0.094-0.616)	NA	0.029 (0.0011-0.102)			0.059 (0.009-0.105)	1.68 (1.09-2.43)	2.41 (1.11-4.12)	-24291
Tomashek [38]	2007		0.010 (0.001-0.020)	0.053 (0.030-0.695)	NA	0.026 (0.010-0.076)			0.040 (0.002-0.079)	1.38 (1.02-1.83)	1.69 (1.02-2.75)	-7042
Anker [18]	1999/05	Singapore	0.016 (0.003-0.069)	0.134 (0.087-0.474)	NA	0.323 (0.017-0.952)	0.056 (0.002-0.307)	0.519 (0.026-0.975)	0.062 (0.013-0.276)	1.86 (1.15-5.51)	2.78 (1.22-10.06)	-16182
Koh [39]	2005		0.011 (0.005-0.014)	0.268 (0.212-0.465)	NA	0.008 (0.003-0.040)			0.042 (0.020-0.056)	1.41 (1.19-1.57)	1.77 (1.29-2.15)	-20559
Ler [23]	2005/07		0.009 (0.003-0.013)	0.242 (0.181-0.646)	NA	0.006 (0.00310.046)			0.036 (0.013-0.051)	1.36 (1.12-1.52)	1.63 (1.16-2.03)	-40149
Anker [18]	1996/05	Sri Lanka	0.019 (0.004-0.077)	0.069 (0.037-0.156)	NA	0.189 (0.004-0.911)	0.183 (0.014-0.669)	0.440 (0.030-0.966)	0.077 (0.016-0.306)	1.61 (1.12-4.20)	2.22 (1.16-8.83)	-16555
Kulatilaka [40]	2010		0.058 (0.032-0.090)	0.001 (0.000-0.001)	0.058 (0.014-0.103)	0.008 (0.003-0.028)			0.234 (0.129-0.361)	4.16 (2.725.83)	6.93 (4.31-10.41)	-1565
Lin [25]	2003/09	Taiwan	0.001 (0.000-0.004)	0.135 (0.006-0.901)	NA	0.010 (0.003-0.053)	0.034 (0.006-0.389)	0.516 (0.019-0.980)	0.002 (0.001-0.015)	1.02 (1.01-1.14)	1.02 (1.01-1.20)	-4241
Limkittikul [41]	2000/10	Thailand	0.040 (0.030-0.052)	0.060 (0.049-0.070)	0.026 (0.006-0.065)	0.015 (0.005-0.062)	0.018 (0.002-0.069)	0.532 (0.030-0.978)	0.160 (0.118-0.210)	2.84 (2.26-3.58)	5.39 (3.92-7.18)	-1387845
Wichmann [34]	2006/07		0.057 (0.024-0.198)	0.470 (0.351-0.945)	NA	0.203 (0.013-0.907)			0.230 (0.095-0.790)	1.53 (1.20-3.94)	2.10 (1.29-13.76)	-146
Bangkok [26]	2000		0.047 (0.038-0.055)	0.055 (0.052-0.058)	NA	0.013 (0.006-0.029)			0.188 (0.151-0.219)	3.30 (2.73-3.84)	7.17 (5.49-8.69)	-11467
Ratchaburi [26]	2000		0.050 (0.043-0.058)	0.108 (0.103-0.115)	NA	0.012 (0.005-0.033)			0.202 (0.170-0.232)	3.37 (2.92-3.82)	6.59 (5.51-7.67)	-2810
Rayong [26]	2010		0.025 (0.023-0.027)	0.113 (0.103-0.122)	NA	0.001 (0.001-0.002)			0.099 (0.092-0.108)	1.99 (1.91-2.09)	3.25 (3.02-3.50)	-4128
Cuong [30]	1998/09	Vietnam	0.015 (0.005-0.019)	0.024 (0.019-0.031)	NA	0.010 (0.004-0.028)	0.045 (0.002-0.610)	0.504 (0.021-0.979)	0.061 (0.021-0.076)	1.53 (1.17-1.69)	2.04 (1.26-2.43)	-58901

*Assumption 1: individuals can be infected up to four times. Assumption 2: individuals are immune after two infections.

Table S6: Summary parameter estimates from model 2 fitted to cumulative incidence data by country (age-dependent reporting rate, model 2B).

Author [Ref]	Year	Country	λ (95%CrI)	ρ_{young} (95%CrI)	ρ_{old} (95%CrI)	B (95%CrI)	Cutoff (95%CrI)	φ (95%CrI)	γ_1 (95%CrI)	γ_3 (95%CrI)	λ_{tot} (95%CrI)	R_0 (95%CrI)*		LnL
												**1**	**2**	
Cordeiro [32]	1995/01	Brazil	0.018 (0.010-0.030)	0.481 (0.163-0.943)	0.460 (0.096-0.931)	NA	65 (15-79)	0.007 (0.001-0.041)	0.050 (0.003-0.178)	0.491 (0.028-0.964)	0.036 (0.021-0.062)	1.57 (1.32-2.05)	1.57 (1.32-2.05)	-355181
	2002/06		0.012 (0.007-0.018)	0.531 (0.319-0.958)	0.494 (0.059-0.923)	NA	65 (100-79)	0.006 (0.002-0.039)			0.035 (0.021-0.053)	1.37 (1.22-1.58)	1.48 (1.27-1.81)	-326485
Cardoso [16]	2000/09		0.012 (0.009-0.015)	0.061 (0.015-0.119)	0.350 (0.025-0.973)	NA	90 (20-90)	0.002 (0.001-0.008)			0.037 (0.027-0.046)	1.43 (1.30-1.55)	1.58 (1.38-1.76)	-4333
Penna [12]	2001/06		0.006 (0.002-0.183)	0.262 (0.004-0.910)	0.390 (0.046-0.968)	0.014 (0.001-0.613)	50 (15-90)	0.040 (0.003-0.276)			0.024 (0.009-0.734)	1.17 (1.06-8.49)	1.25 (1.07-17.09)	-55911
Vong [33]	2006/08	Cambodia	0.066 (0.059-0.073)	0.183 (0.156-0.193)	0.051 (0.007-0.251)	NA	19 (14-21)	0.002 (0.001-0.005)	0.036 (0.002-0.190)	0.491 (0.028-0.964)	0.265 (0.234-0.292)	1.94 (1.81-2.07)	3.22 (2.84-3.58)	-55927
Wichmann [34]	2006/07		0.030 (0.010-0.179)	0.244 (0.078-0.931)	0.495 (0.038-0.960)	NA	15 (5-15)	0.183 (0.007-0.889)			0.117 (0.041-0.715)	1.25 (1.08-3.50)	1.40 (1.10-11.27)	-31
Luo [45]	1978/88	China	0.017 (0.006-0.073)	0.001 (0.000-0.001)	0.486 (0.024-0.969)	0.378 (0.043-0.748)	90 (90-90)	0.002 (0.001-0.024)	0.051 (0.002-0.366)	0.546 (0.031-0.981)	0.071 (0.024-0.292)	1.82 (1.24-6.36)	2.82 (1.39-16.07)	-12911
	1989/99		0.011 (0.002-0.040)	0.001 (0.000-0.001)	0.486 (0.032-0.975)	0.087 (0.002-0.701)	90 (90-90)	0.003 (0.001-0.036)			0.042 (0.007-0.162)	1.44 (1.07-3.30)	1.84 (1.08-7.34)	-12430
	2000/09		0.012 (0.001-0.084)	0.001 (0.00-0.008)	0.511 (0.024-0.978)	0.199 (0.002-0.550)	90 (90-90)	0.052 (0.014-0.256)			0.047 (0.001-0.336)	1.50 (1.01-7.48)	1.98 (1.01-18.69)	-4413
Guo [46]	2005/11		0.004 (0.001-0.010)	0.001 (0.00-0.007)	0.487 (0.013-0.973)	0.007 (0.001-0.121)	90 (90-90)	0.029 (0.009-0.104)			0.018 (0.002-0.040)	1.15 (1.02-1.36)	1.22 (1.02-1.63)	-3459

*Assumption 1: individuals can be infected up to four times. Assumption 2: individuals are immune after two infections.

Table S6 continued (2/3).

Author [Ref]	Year	Country	λ (95%CrI)	ρ_{young} (95%CrI)	ρ_{old} (95%CrI)	β (95%CrI)	Cutoff (95%CrI)	φ (95%CrI)	γ_1 (95%CrI)	γ_3 (95%CrI)	λ_{tot} (95%CrI)	R_0 (95%CrI)*		LnL
												1	2	
Anker [18]	2000/06	Laos	0.036 (0.001-0.158)	0.040 (0.010-0.949)	0.401 (0.066-0.955)	NA	15 (5-15)	0.204 (0.004-0.908)	0.223 (0.006-0.901)	0.468 (0.039-0.971)	0.143 (0.002-0.632)	2.29 (1.02-6.13)	3.19 (1.02-12.33)	-45134
Khampa-pongpane [43]	2010		0.011 (0.001-0.083)	0.136 (0.016-0.862)	0.238 (0.050-0.885)	0.055 (0.003-0.576)	30 (5-40)	0.072 (0.007-0.432)			0.044 (0.003-0.330)	1.31 (1.02-3.62)	1.52 (1.02-6.58)	-33953
Prasith [44]	2010		0.027 (0.002-0.176)	0.083 (0.019-0.374)	0.426 (0.035-0.963)	0.270 (0.009-0.937)	80 (15-80)	0.348 (0.074-0.926)			0.108 (0.006-0.703)	1.89 (1.04-7.51)	2.82 (1.05-12.89)	-22762
Hammond [20]	1999/01	Nicaragua	0.012 (0.001-0.034)	0.051 (0.014-0.937)	0.407 (0.022-0.947)	NA	55 (10-55)	0.017 (0.004-0.057)	0.459 (0.030-0.972)	0.488 (0.030-0.974)	0.036 (0.002-0.102)	1.26 (1.01-1.80)	1.32 (1.01-2.15)	-1183
Anker [18]	1998-2005	Philippines	0.010 (0.004-0.098)	0.277 (0.047-0.943)	0.188 (0.043-0.829)	NA	15 (5-15)	0.301 (0.016-0.927)	0.166 (0.015-0.336)	0.428 (0.020-0.965)	0.042 (0.014-0.391)	1.41 (1.14-4.45)	1.73 (1.20-7.46)	-166118
Rigau-Perez [35]	1994	Puerto Rico	0.022 (0.013-0.029)	0.456 (0.280-0.920)	0.506 (0.263-0.967)	NA	55 (20-99)	0.003 (0.001-0.015)	0.163 (0.006-0.605)	0.560 (0.044-0.971)	0.065 (0.040-0.087)	1.89 (1.51-2.24)	2.29 (1.70-2.88)	-50387
	1995/97		0.020 (0.003-0.027)	0.040 (0.026-0.526)	0.353 (0.030-0.968)	NA	85 (30-85)	0.008 (0.003-0.173)			0.060 (0.010-0.080)	1.74 (1.11-2.03)	2.07 (1.13-2.55)	-9113
Ramos [36]	2006		0.017 (0.007-0.036)	0.460 (0.205-0.898)	0.470 (0.053-0.957)	NA	40 (10-61)	0.034 (0.014-0.346)			0.070 (0.026-0.145)	1.64 (1.21-2.56)	2.32 (1.33-5.01)	-203
Sharp [37]	2010		0.014 (0.006-0.030)	0.197 (0.102-0.942)	0.238 (0.110-0.929)	NA	50 (20-81)	0.019 (0.003-0.067)			0.055 (0.024-0.116)	1.62 (1.25-2.51)	2.27 (1.41-4.56)	-24289
Tomashek [38]	2007		0.010 (0.001-0.022)	0.089 (0.038-0.916)	0.218 (0.045-0.923)	NA	60 (20-85)	0.022 (0.005-0.080)			0.035 (0.003-0.088)	1.34 (1.02-1.94)	1.59 (1.02-3.03)	-7041

*Assumption 1: individuals can be infected up to four times. Assumption 2: individuals are immune after two infections.

Table S6 continued (3/3).

**Author [Ref]**	**Year**	**Country**	λ **(95%CrI)**	ρ_{young} **(95%CrI)**	ρ_{old} **(95%CrI)**	B **(95%CrI)**	**Cutoff (95%CrI)**	φ **(95%CrI)**	γ_1 **(95%CrI)**	γ_3 **(95%CrI)**	λ_{tot} **(95%CrI)**	R_0 **(95%CrI)***		**LnL**
												**1**	**2**	
Anker [18]	1999/ 05	Singapore	0.011 (0.002-0.115)	0.201 (0.012-0.936)	0.239 (0.056-0.942)	NA	15 (5-15)	0.312 (0.017-0.952)	0.057 (0.002-0.458)	0.564 (0.032-0.970)	0.041 (0.007-0.459)	1.54 (1.09-8.69)	2.05 (1.12-17.32)	-16182
Koh [39]	2005		0.010 (0.003-0.015)	0.267 (0.186-0.854)	0.375 (0.090-0.917)	NA	65 (25-85)	0.008 (0.002-0.047)			0.042 (0.011-0.061)	1.40 (1.09-1.63)	1.74 (1.12-2.30)	-20560
Ler [23]	2005/ 07		0.009 (0.002-0.014)	0.242 (0.152-0.721)	0.344 (0.031-0.945)	NA	80 (15-80)	0.006 (0.001-0.057)			0.036 (0.010-0.056)	1.35 (1.09-1.58)	1.63 (1.11-2.19)	-40150
Anker [18]	1996/ 05	Sri Lanka	0.001 (0.000-0.186)	0.159 (0.001-0.950)	0.121 (0.006-0.766)	NA	15 (5-15)	0.225 (0.011-0.907)	0.272 (0.25-0.899)	0.515 (0.031-0.973)	0.004 (0.001-0.744)	1.02 (1.00-11.04)	1.03 (1.00-27.67)	-16555
Kulatilaka [40]	2010		0.065 (0.034-0.137)	0.001 (0.001-0.001)	0.501 (0.007-0.974)	0.086 (0.026-0.394)	65 (65-65)	0.009 (0.003-0.039)			0.260 (0.137-0.550)	4.51 (2.83-8.32)	7.60 (4.51-16.46)	-1566
Lin [25]	2003/ 09	Taiwan	0.001 (0.000-0.003)	0.186 (0.008-0.933)	0.411 (0.031-0.961)	NA	75 (15-85)	0.010 (0.003-0.053)	0.036 (0.005-0.387)	0.514 (0.031-0.969)	0.002 (0.001-0.011)	1.01 (1.01-1.11)	1.02 (1.01-1.14)	-4241
Limkittikul [41]	200./ 10	Thailand	0.042 (0.030-0.067)	0.051 (0.035-0.068)	0.354 (0.022-0.974)	0.053 (0.011-0.137)	80 (15-80)	0.023 (0.006-0.105)	0.022 (0.002-0.065)	0.546 (0.024-0.978)	0.168 (0.120-0.269)	2.95 (2.29-4.52)	5.66 (3.99-9.38)	-1387846
Wichmann [34]	2006/ 07		0.062 (0.024-0.195)	0.465 (0.278-0.944)	0.588 (0.037-0.985)	NA	10 (5-15)	0.176 (0.006-0.902)			0.249 (0.096-0.779)	1.59 (1.20-3.87)	2.26 (1.30-13.37)	-146
Cuong [30]	1998/ 09	Vietnam	0.021 (0.008-0.027)	0.011 (0.005-0.056)	0.025 (0.017-0.815)	NA	15 (15-79)	0.003 (0.001-0.021)	0.057 (0.002-0.270)	0.543 (0.038-0.982)	0.084 (0.031-0.107)	1.76 (1.26-2.02)	2.61 (1.42-3.27)	-58897

The baseline reporting rate (ρ), which is defined as the probability of detecting a secondary infection, was 10% (range: 1% - 79%) and 16% (range: 1%-55%) when fitted to yearly and cumulative incidence data respectively, assuming a single reporting rate for all ages. When we allowed for age-dependent reporting rates, estimates of ρ increased to between 26% - 33% and 13% - 37% when fitted to yearly and cumulative incidence data respectively. Figure S4 shows the dataset-specific estimates of the baseline reporting rate (ρ , ρ_{young} , and ρ_{old}) and the probability of detecting a primary (γ_1) case relative to the baseline reporting rate, and the probability of detecting a tertiary/quaternary (γ_3) case relative to a primary case by model type. The probability of detecting a primary case relative to a secondary case was consistently low at less than 25% for the majority of data sets and models.

When we allowed for reporting rates to vary by age, we found that the median probability that a secondary infection was detected was higher in older compared with younger individuals (Figure S5). However, stratifying parameter estimates by country, when ρ_{old} was higher than ρ_{young} the corresponding estimate of the age threshold at which reporting rates changed ($A_{threshold}$) were high (Figure S5, Table S4 and Table S6). In such cases the reporting rate ρ_{young} applied to the majority of age groups and the estimated values were comparable to the corresponding baseline reporting rates estimated from the model variants which did not incorporate age-dependent reporting.

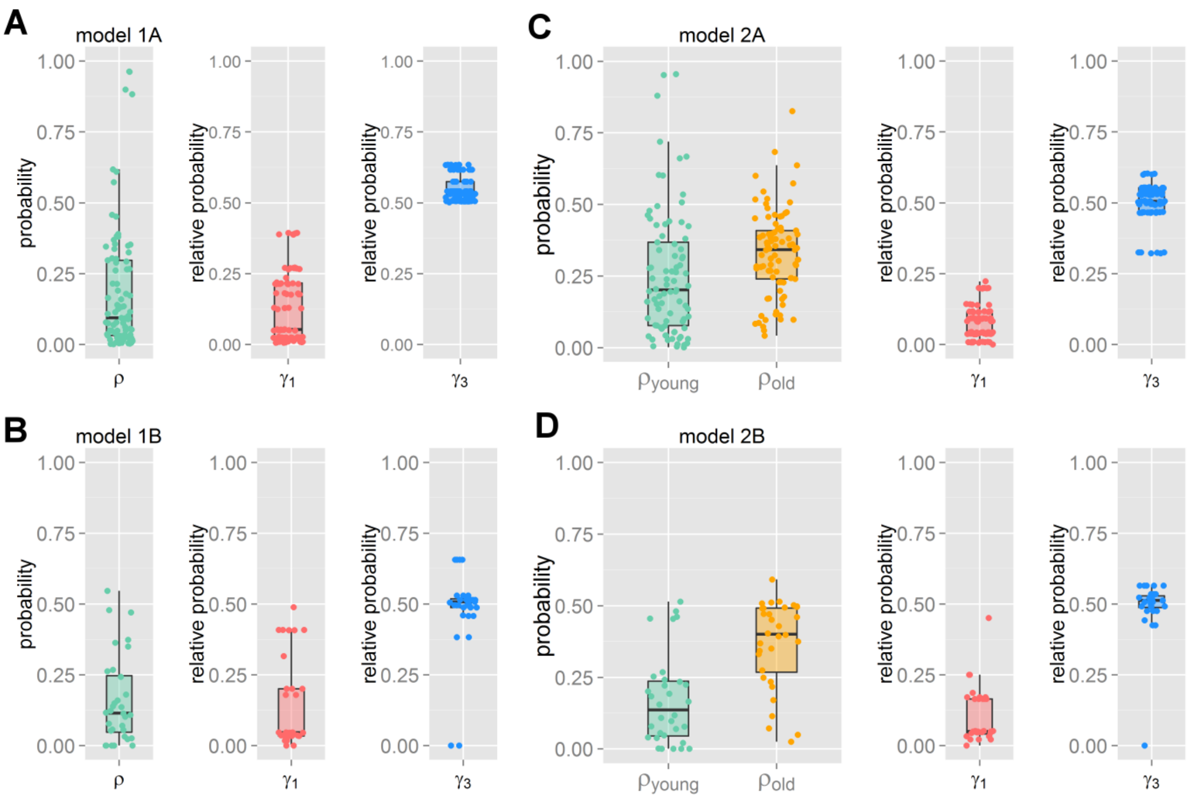

Figure S4: Boxplots of estimated reporting rates by model type: A) model 1A, B) model 1B, C) model 2A and D) model 2B. Each point represents the posterior median estimate for one dataset. ρ, ρ_{young} , ρ_{old} **= baseline reporting rate or probability of detecting a secondary infection for all ages, individuals younger than cut off, and individuals older than the cut off age respectively.** γ_1 **= probability of detecting a primary infection relative to a secondary infection, and** γ_3 **= probability of detecting a tertiary/quaternary infection relative to a primary infection. Models 1A and 1B were fitted to yearly and cumulative incidence data respectively assuming a non-age dependent reporting rate, while models 2A and 2B were fitted to yearly and cumulative incidence data respectively assuming an age-dependent reporting rate.**

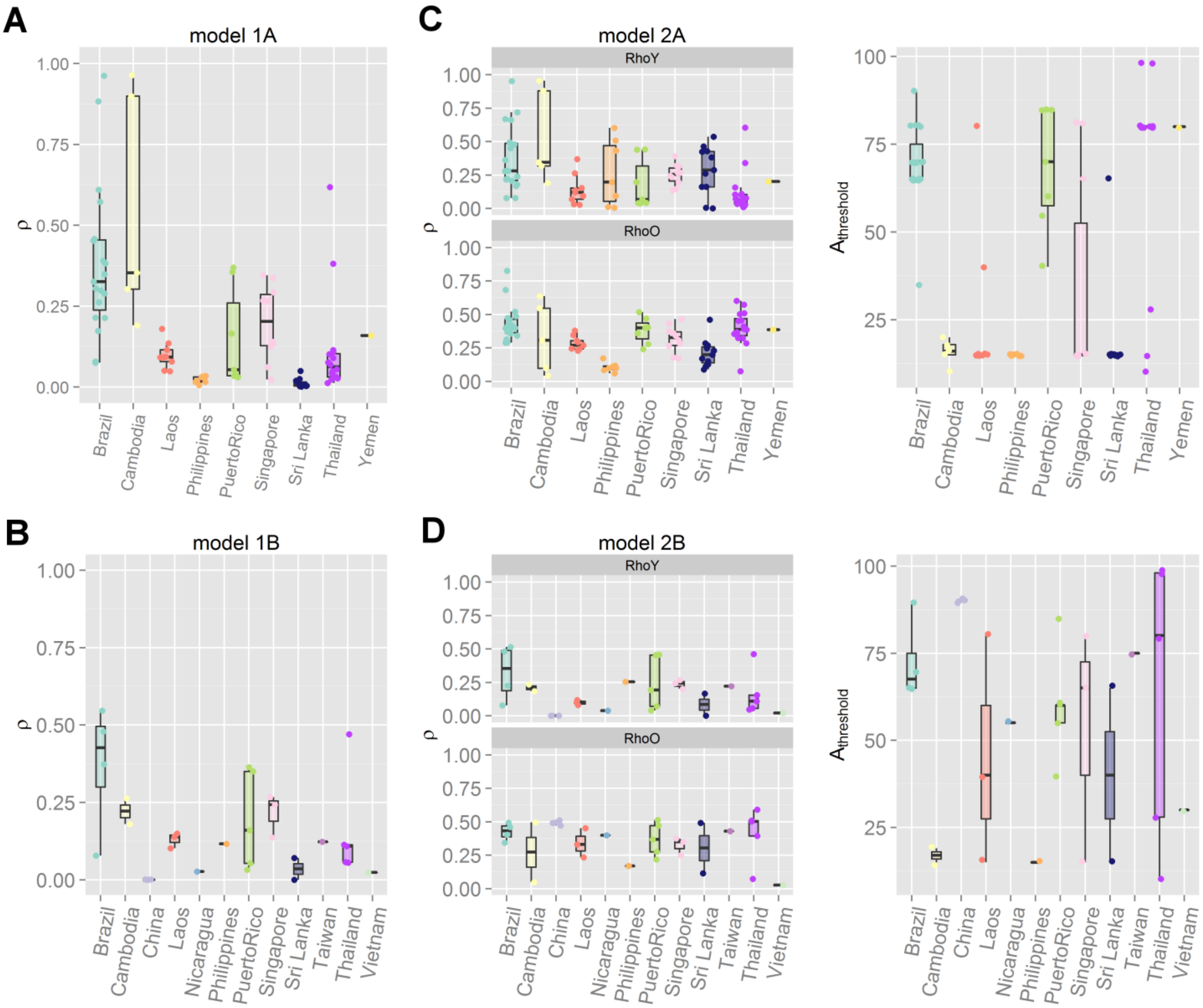

FigureS5: Comparison of baseline reporting rates (ρ, ρ_y, ρ_o) and estimated age (years) at which reporting rates change ($A_{threshold}$) by model. A) Model 1A, with single reporting rate fitted to yearly incidence data, B) model 1B, with single reporting rate fitted to cumulative incidence data, C) model 2A, with age-dependent reporting rate fitted to yearly incidence data, and D) model 2B, with age-dependent reporting rate fitted to cumulative incidence data. Each point represents the posterior median estimate for one dataset.

The baseline reporting rates ρ (Figure S5) varied substantially by country, likely reflecting differences in healthcare seeking behaviour and surveillance.

Comparing models fitted to yearly incidence and cumulative incidence data, models 2 provided a better fit to the data 62% and 59% of the time respectively, as assessed by the DIC (Table S7 and Table S8).

2.2 DIC Comparison

Table S7: Summary DIC comparison of model fits to yearly incidence data. Models 1 (single reporting rate) and 2 (age-dependent reporting rate) fitted to yearly incidence data. Smaller DIC for each dataset is highlighted in bold where the difference is greater than 5.

**Author [Ref]**	**Year**	**Number of serotypes**	**Country**	**DIC**	
				**Model 1**	**Model 2**
Cordeiro [32]	1995	2	Brazil	35710	**35703**
	1996	2		80771	80769
	1997	2		115423	**115414**
	1998	2		186560	**186511**
	1999	2		128229	**128227**
	2000	3		101163	101163
	2001	3		62048	**62035**
	2002	3		418449	418448
	2003	3		95070	**94633**
	2004	3		23166	23163
	2005	3		47099	47095
	2006	3		68804	**68797**
Penna [12]	2001	4		**27771**	28830
	2002	4		**13788**	17822
	2003	4		**20633**	20681
	2004	4		**3032**	10996
	2005	4		**16643**	16998
	2006	4		**15566**	15592
Vong [33]	2006	4	Cambodia	41711	41710
	2007	4		221410	**221390**
	2008	4		68562	**68544**
Wichmann [34]	2006	4		65	65
	2007	4		1088	1087
Anker [18]	2000	4	Laos	2104	**2055**
	2001	4		7257	**6585**
	2002	4		16365	**15530**
	2003	4		34578	**32495**
	2004	4		6557	**4009**
	2005	4		10482	**9734**
	2006	4		11876	**10165**
Khampapongpane [43]	2010	4		**64888**	67566
Prasith [44]	2010	4		45456	**44129**
Anker [18]	1998	4	Philippines	67732	**65607**
	1999	4		18300	**11191**
	2001	4		**47633**	47674
	2002	4		32395	**32374**
	2003	4		57113	**53741**
	2004	4		**44004**	44018
	2005	3		64390	**64353**
Rigau-Perez [35]	1994	3	Puerto Rico	100776	**100727**
	1995	3		6127	6128
	1996	3		5393	5396
	1997	4		**6742**	6747
Ramos [36]	2006	4		409	409
Sharp [37]	2007	4		**48497**	48514
Tomashek [38]	2010	4		14076	**14014**
Anker [18]	1999	4	Singapore	1041	**785**
	2000	4		725	**474**
	2001	4		1879	**690**
	2002	4		3013	**917**
	2003	4		3719	**1133**
	2004	4		8270	**7606**
	2005	4		13714	**13514**
Koh [39]	2005	4		41122	41121
Ler [23]	2005	4		50174	50175
	2007	4		29440	29440

Table S7 continued (2/2).

Author	Year	# serotypes	Country	DIC	
				**Model 1**	**Model 2**
Anker [18]	1996	4	Sri Lanka	2279	**1967**
	1997	4		1621	1621
	1998	4		**1980**	2210
	1999	4		2350	**1800**
	2000	4		4605	**3594**
	2001	4		**4422**	4672
	2002	4		4509	**3736**
	2003	4		2426	2425
	2004	4		5722	**1706**
	2005	4		1763	**1738**
Kulatilaka [40]	1997	4		**3124**	3138
Limkittikul [41]	2000	4	Thailand	**58282**	59755
	2001	4		**450738**	451213
	2002	4		**371748**	376250
	2003	4		**206304**	214588
	2004	4		**124400**	133462
	2005	4		**157060**	159895
	2006	4		**156750**	160942
	2007	4		**225396**	225623
	2008	4		**318403**	318907
	2009	4		**190752**	206441
	2010	4		**430542**	430608
Wichmann [34]	2006	4		64	64
	2007	4		227	228
Bangkok [26]	2000	4		22938	22939
Ratchaburi [26]	2000	4		5624	5624
Rayong [26]	2010	4		8259	**8236**
Ghouth [42]	2010	3	Yemen	3582	**3576**

Table S8: Summary DIC comparison of model fits to cumulative incidence data. Models 1 (single reporting rate) and 2 (age-dependent reporting rate) fitted to cumulative incidence data. Smaller DIC for each dataset is highlighted in bold where the difference is greater than 5.

Author	Year	Country	Model 1	Model 2
Cordeiro [32]	1995/01	Brazil	710366	**710318**
	2002/06		**652964**	652966
Cardoso [16]	2000/09		**8670**	8671
Penna [12]	2001/06		111819	**111696**
Vong [33]	2006/08	Cambodia	111870	**111861**
Wichmann [34]	2006/07		64	64
Luo [45]	1978/88	China	25826	25826
	1989/99		**23810**	24861
	2000/09		8823	**8818**
Guo [46]	2005/11		6914	**6895**
Anker [18]	2000/06	Laos	90188	**58722**
Hammond [20]	1995/97	Nicaragua	2358	**2335**
Anker [18]	1998/05	Philippines	**332153**	331296
Rigau-Perez [35]	1994	Puerto Rico	100773	**100697**
	1995/97		**18219**	18231
Anker [18]	1999/05	Singapore	32360	**26429**
Ler [23]	2005/07		80303	80304
Anker [18]	1996/05	Sri Lanka	33110	**29570**
Lin [25]	2003/09	Taiwan	8458	8434
Limkittikul [41]	2000/10	Thailand	2775619	2775687
Wichmann [34]	2006/07		292	289
Cuong [30]	1998/09	Vietnam	117807	117801

2.3 Comparison of estimates obtained from seroprevalence and incidence data.

We used weighted regression to compare the force of infection estimates obtained from age-stratified seroprevalence data to cumulative incidence data. Estimates obtained from the model fitted to the cumulative incidence data assuming a single reporting rate (Model 1B) were comparable to force of infection estimates from seroprevalence data. The majority of the total force of infection (λ_{total}) estimates from incidence data (calculated by multiplying the serotype-specific force of infection by the number of serotypes in circulation) were comparable for both models to those obtained from seroprevalence data when λ_{total} was smaller than ~0.1, with greater uncertainty as force of infection increases (Figure S7). The estimates from model 2B (age-dependent reporting rates) produced very similar estimates.

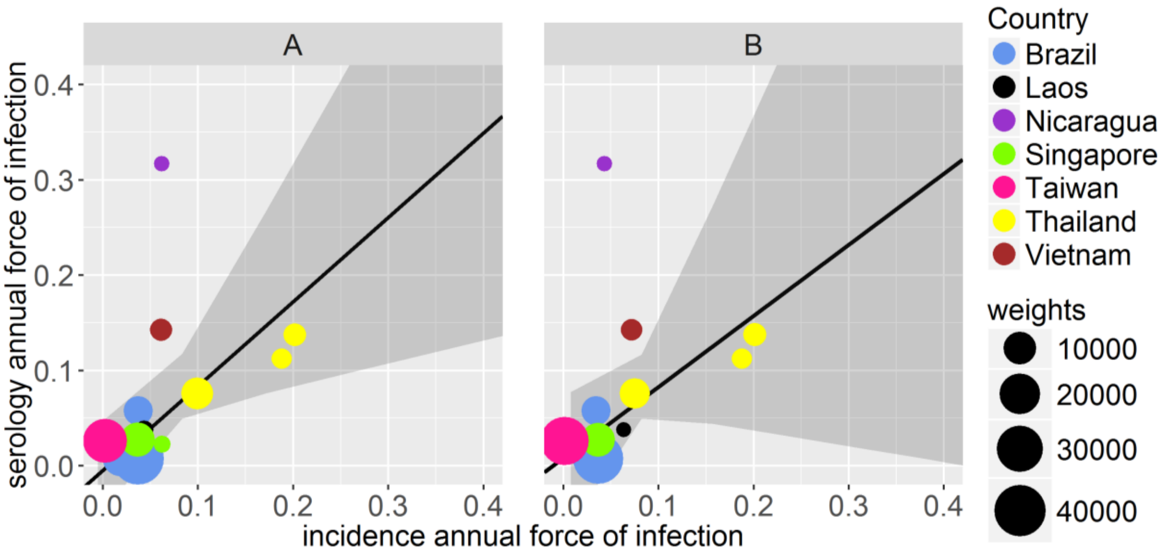

Figure S6: Comparison of weighted deming regression of force of infection estimates by country from cumulative incidence data and seroprevalence data for A) model 1B (non-age dependent reporting) and B) model 2B (age-dependent reporting rate). Each point is weighted depending on the error in both serology and incidence estimates, represented by the size of circles (larger circles indicating greater weight i.e. smaller error). See Table S2 for summary of matched datasets.

For the three locations in Thailand where region and time matching seroprevalence and incidence data were available [26], the force of infection estimates obtained from the models fitted to incidence data were comparable except for Ratchaburi where the estimate obtained from seroprevalence data was larger than that from incidence data (Figure S7).

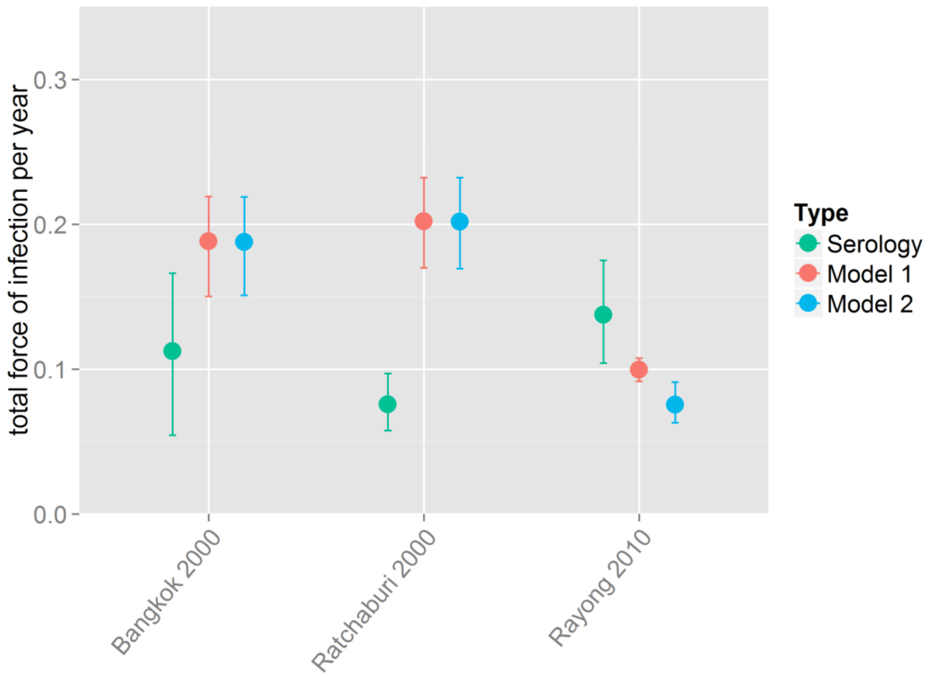

Figure S7 Comparison of force of infection estimates derived from incidence data and seroprevalence data. Comparison of posterior median estimates and 95% CrI of the total force of infection from Models 1 (single reporting rate) and 2 (age-dependent reporting rate) fitted to incidence data and model A (as described in [4]**) to age-stratified seroprevalence data from Thailand where incidence and serology data were available from the same year and location .**

2.4 Model Fits by country

Fig S8: Model fits of models 1 (single reporting rate) and 2 (age-dependent reporting rate) fitted to yearly incidence data from Brazil (Cordeiro *et al*). 95% exact confidence intervals shown around data points, posterior median (line) and 95% credible interval (shaded area) shown.

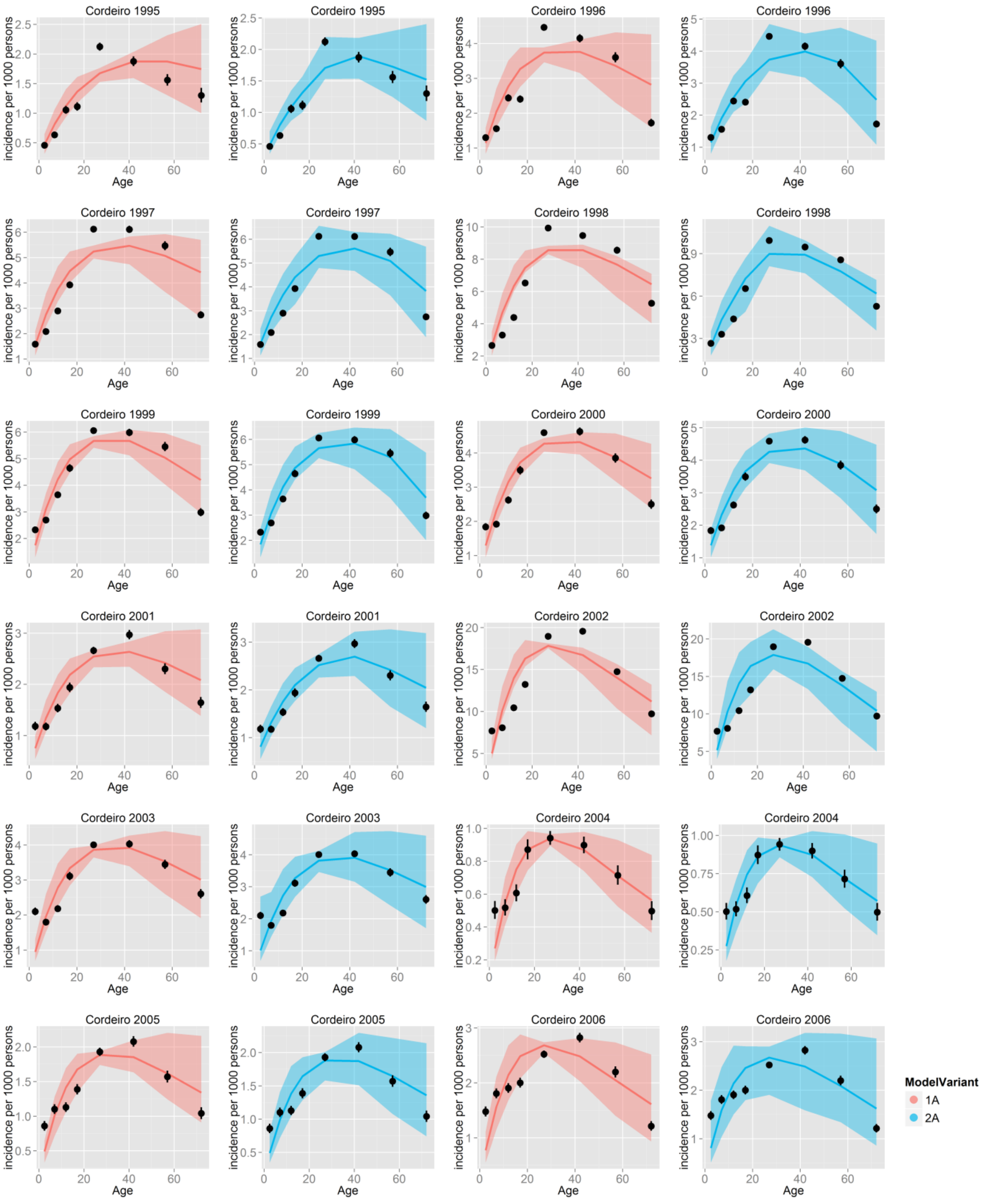

Fig S9: Model fits of models 1 (single reporting rate) and 2 (age-dependent reporting rate) fitted to yearly incidence data from Brazil (Penna *et al*). 95% exact confidence intervals shown around data points, posterior median (line) and 95% credible interval (shaded area) shown.

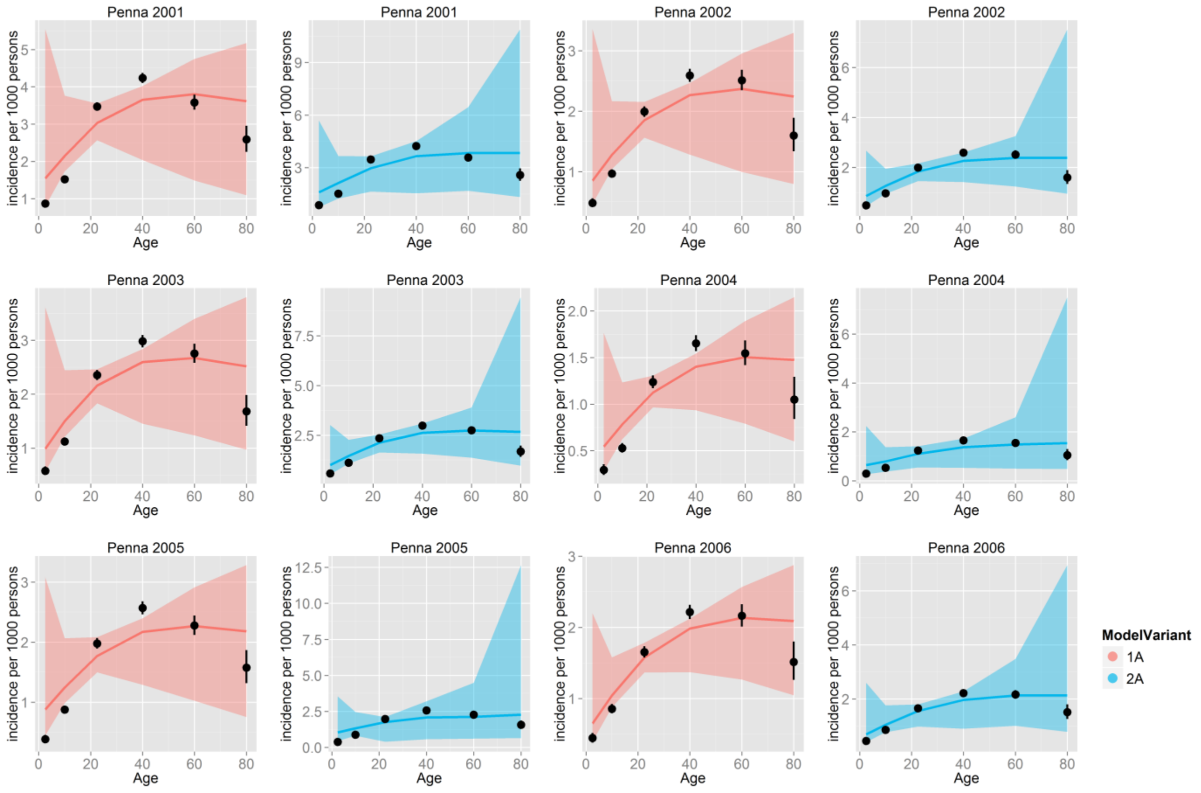

Fig S10: Model fits of model 1 (single reporting rate) and 2 (age-dependent reporting rate) fitted to cumulative incidence data from Brazil. 95% exact confidence interval around data points, posterior median (line) and 95% credible interval (shaded area) shown.

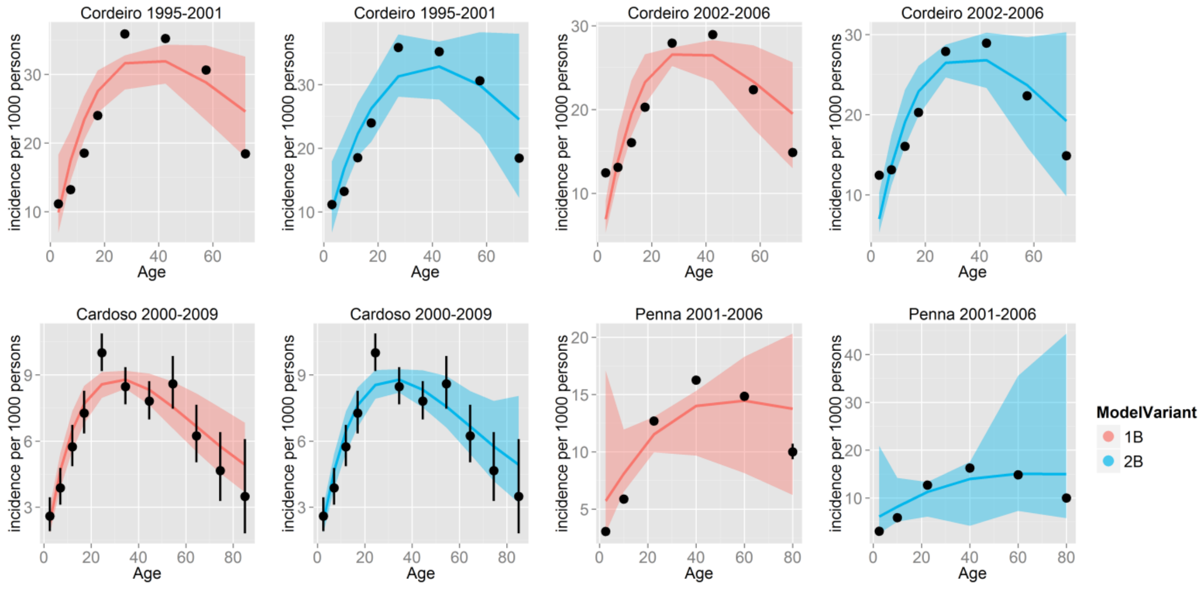

Fig S11: Model fits of models 1 (single reporting rate) and 2 (age-dependent reporting rate) fitted to yearly incidence data from Cambodia. 95% exact confidence interval around data points, posterior median (line) and 95% credible interval (shaded area) shown.

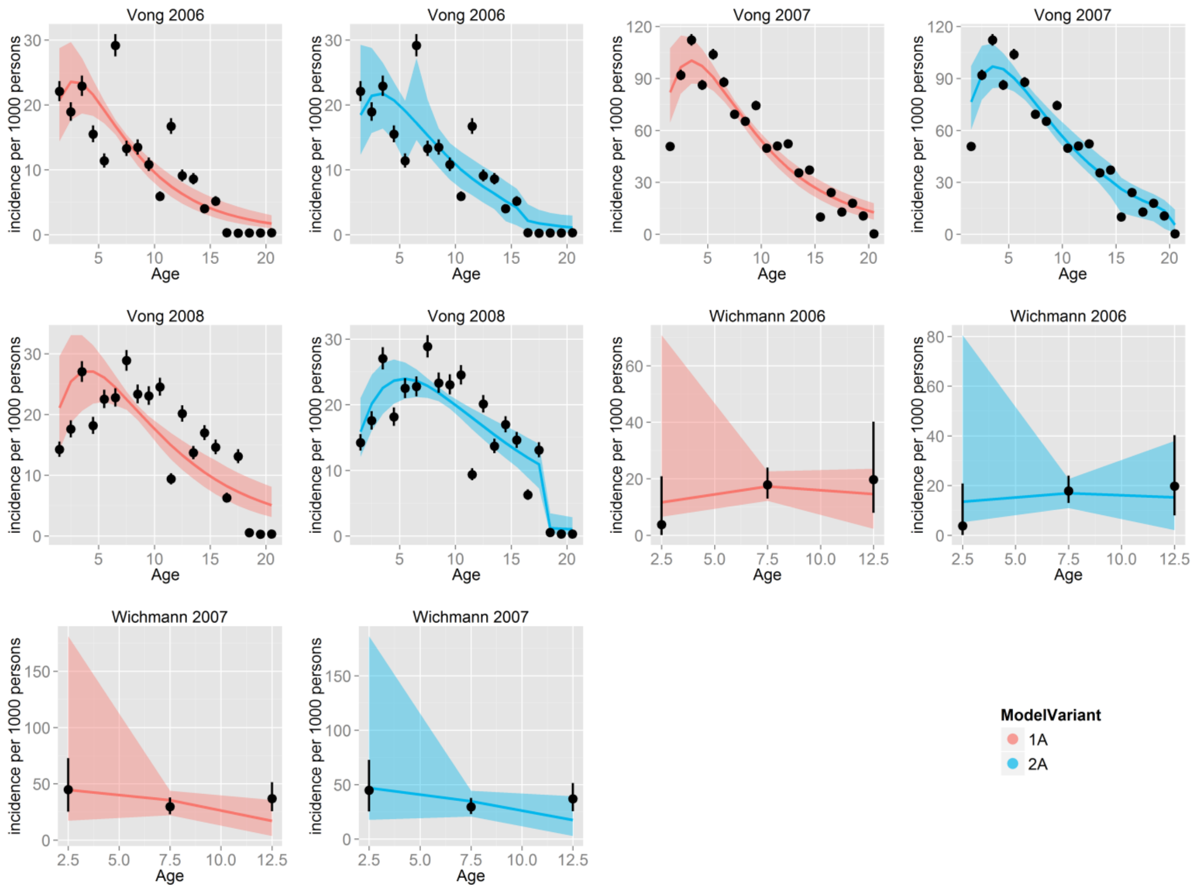

Fig S12: Model fits of models 1 (single reporting rate) and 2 (age-dependent reporting rate) fitted to cumulative incidence data from Cambodia. 95% exact confidence interval around data points, posterior median (line) and 95% credible interval (shaded area) shown.

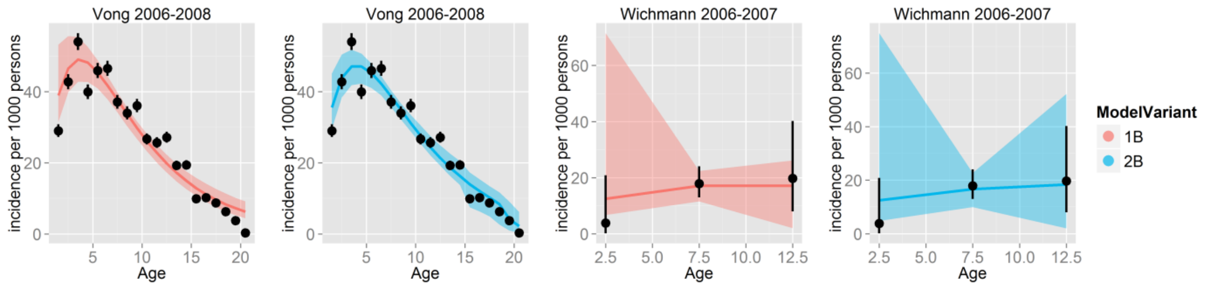

Fig S13: Model fits of models 1 (single reporting rate) and 2 (age-dependent reporting rate) fitted to cumulative incidence data from China. 95% exact confidence interval around data points, posterior median (line) and 95% credible interval (shaded area) shown.

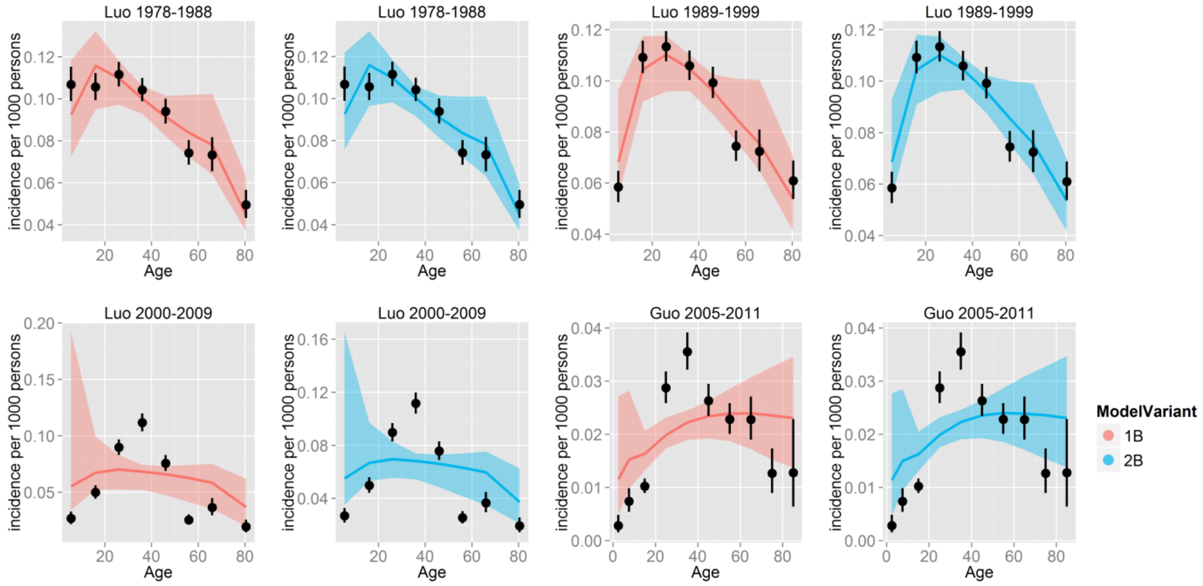

Fig S14: Model fits of model 1 (single reporting rate) and 2 (age-dependent reporting rate) fitted to yearly incidence data from Laos. 95% exact confidence interval around data points, posterior median (line) and 95% credible interval (shaded area) shown.

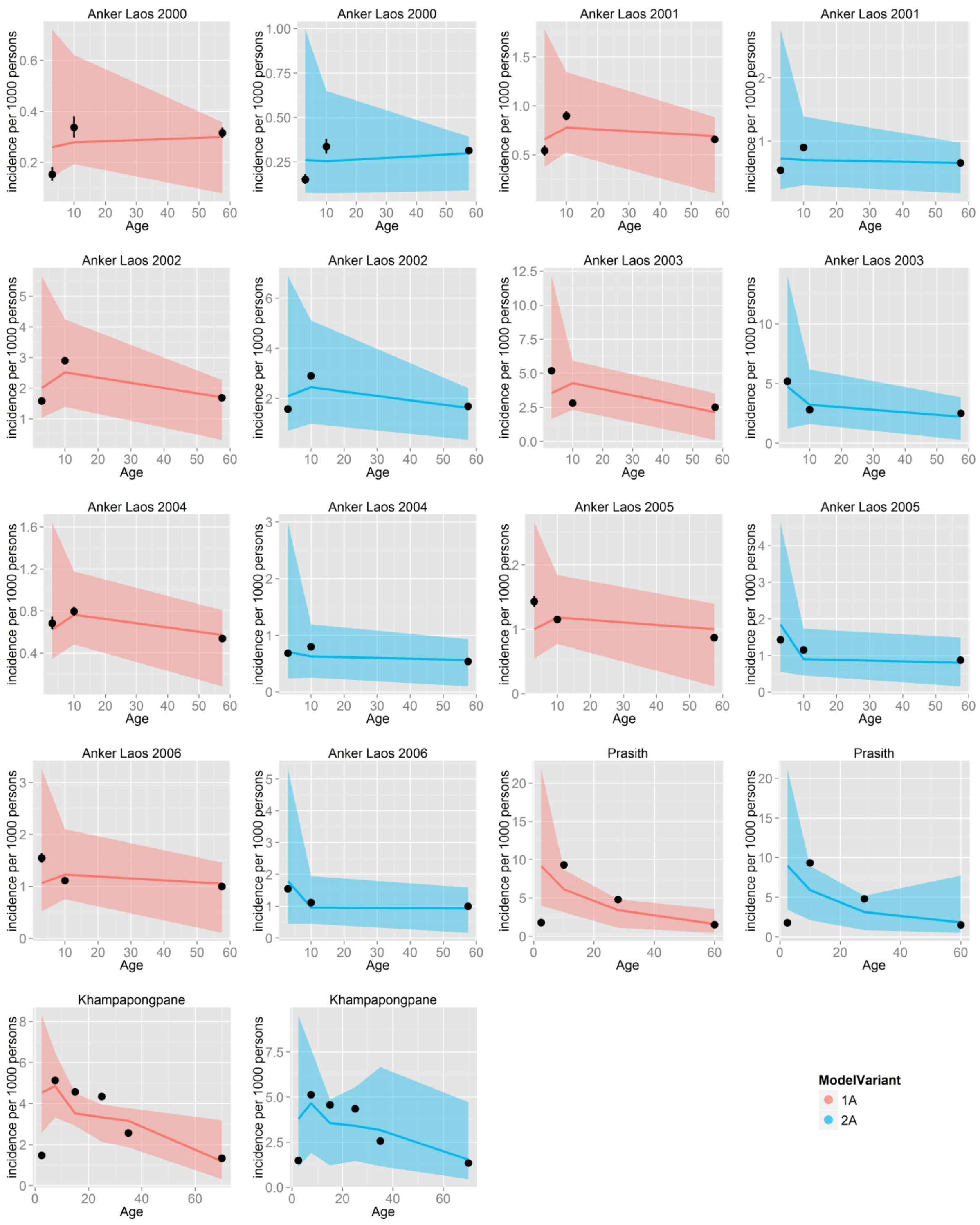

Fig S15: Model fits of model 1 (single reporting rate) and model 2 (age-dependent reporting rate) fitted to cumulative incidence data from Laos. 95% exact confidence interval around data points, posterior median (line) and 95% credible interval (shaded area) shown.

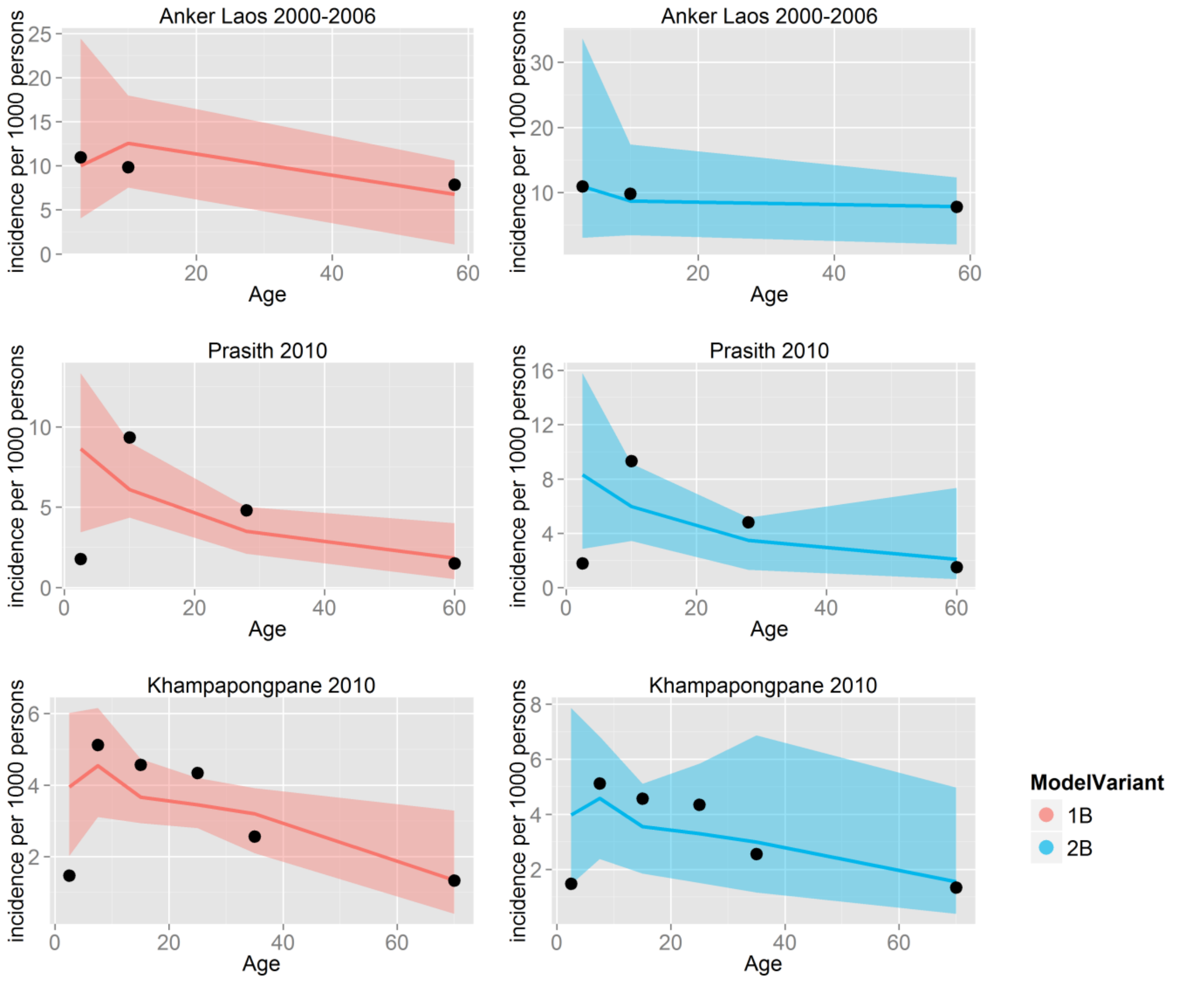

Fig S16: Model fits of model 1 (single reporting rate) and model 2 (age-dependent reporting rate) fitted to cumulative incidence data from Nicaragua. 95% exact confidence interval around data points, posterior median (line) and 95% credible interval (shaded area) shown.

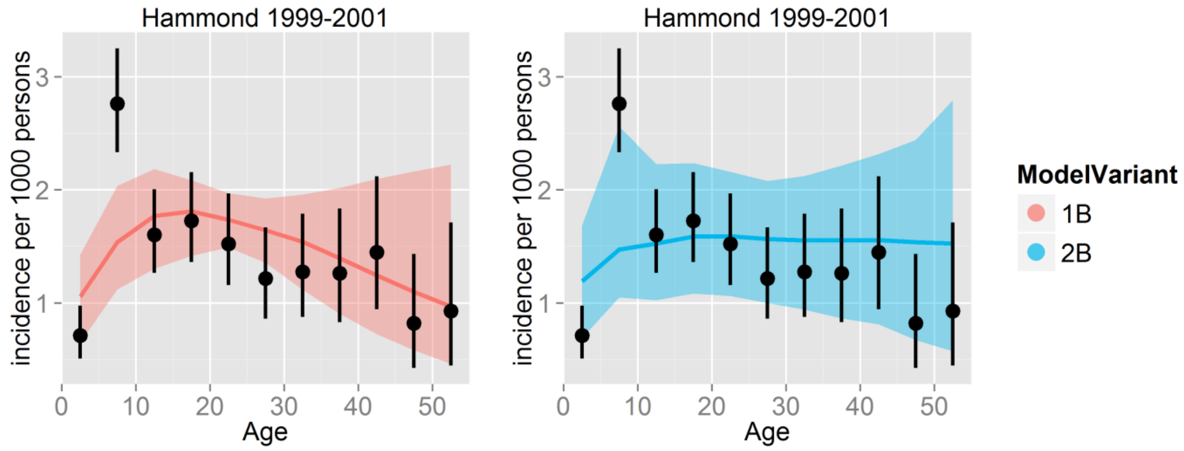

Fig S17: Model fits of model 1 (single reporting rate) and model 2 (age-dependent reporting rate) fitted to yearly incidence data from the Philippines. 95% exact confidence intervals around data points, posterior median (line) and 95% credible intervals (shaded area) shown.

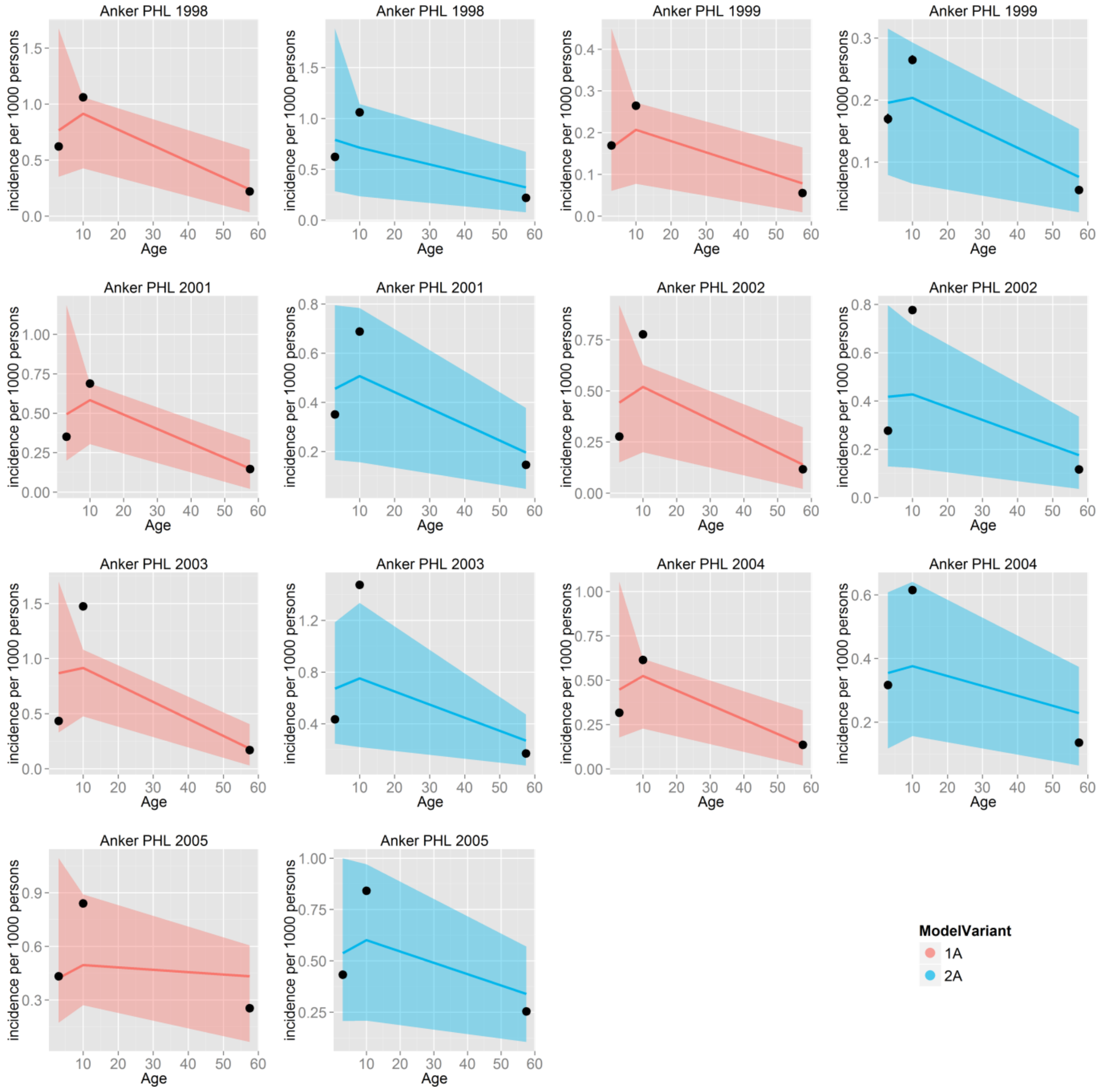

Fig S18: Model fits of model 1 (single reporting rate) and model 2 (age-dependent reporting rate) fitted to cumulative incidence data from the Philippines. 95% exact confidence intervals around data points, posterior median (line) and 95% credible interval (shaded area) shown.

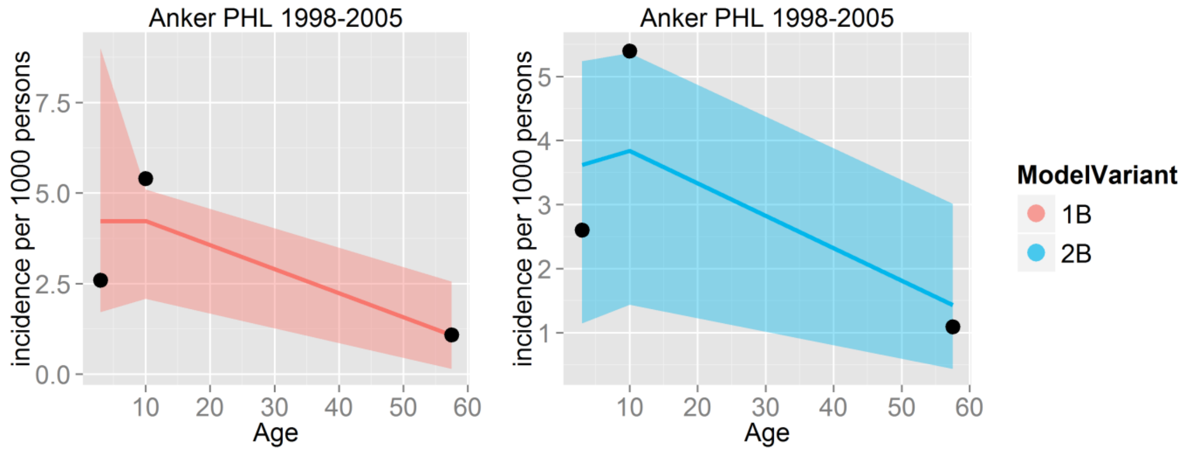

Fig S19: Model fits of model 1 (single reporting rate) and model 2 (age-dependent reporting rate) fitted to yearly incidence data from Puerto Rico. 95% exact confidence intervals around data points, posterior median (line) and 95% credible intervals (shaded area) shown.

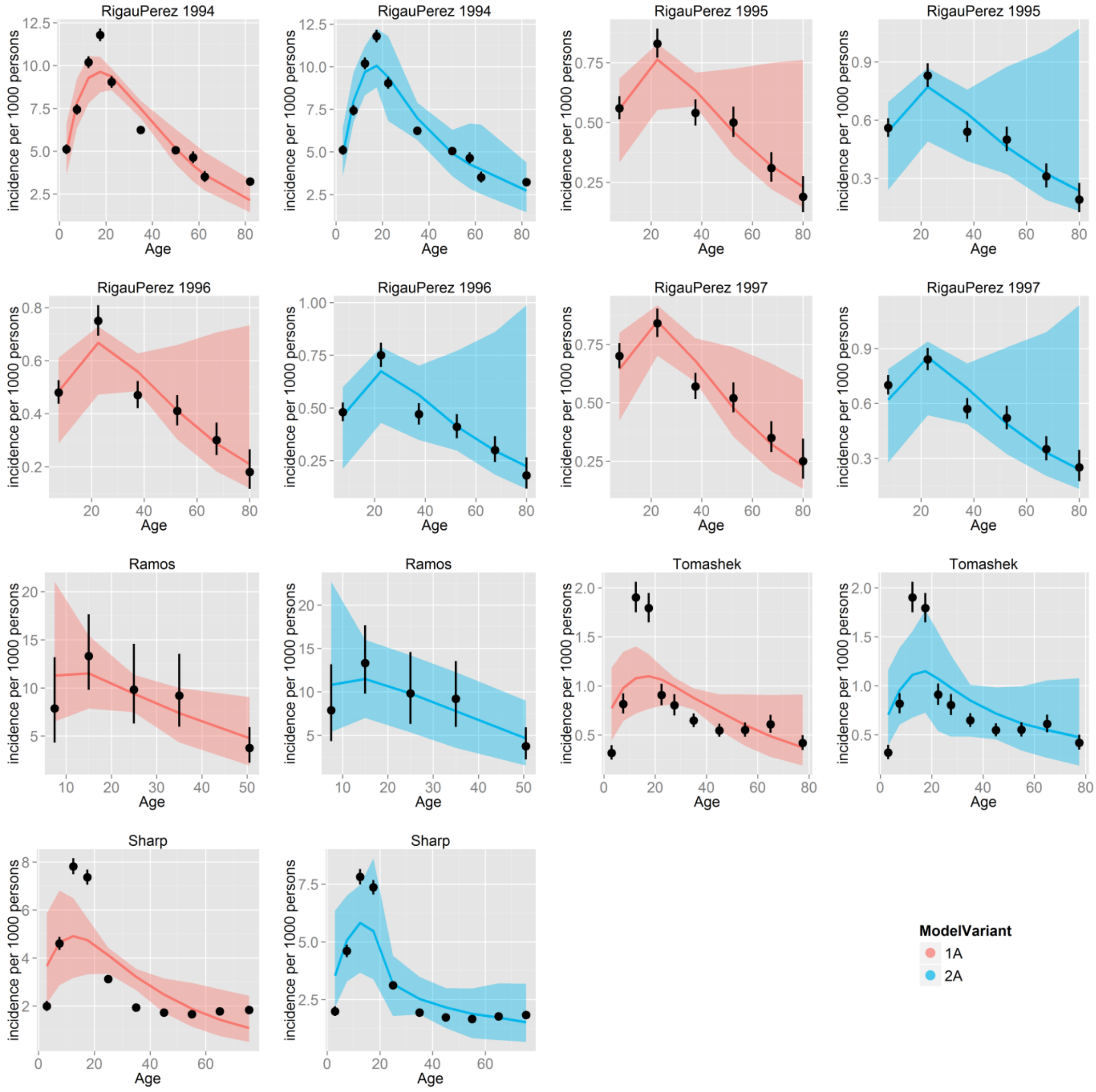

Fig S20: Model fits of model 1 (single reporting rate) and model 2 (age-dependent reporting rate) fitted to cumulative incidence data from Puerto Rico. 95% exact confidence interval around data point, posterior median (line) and 95% credible interval (shaded area) shown.

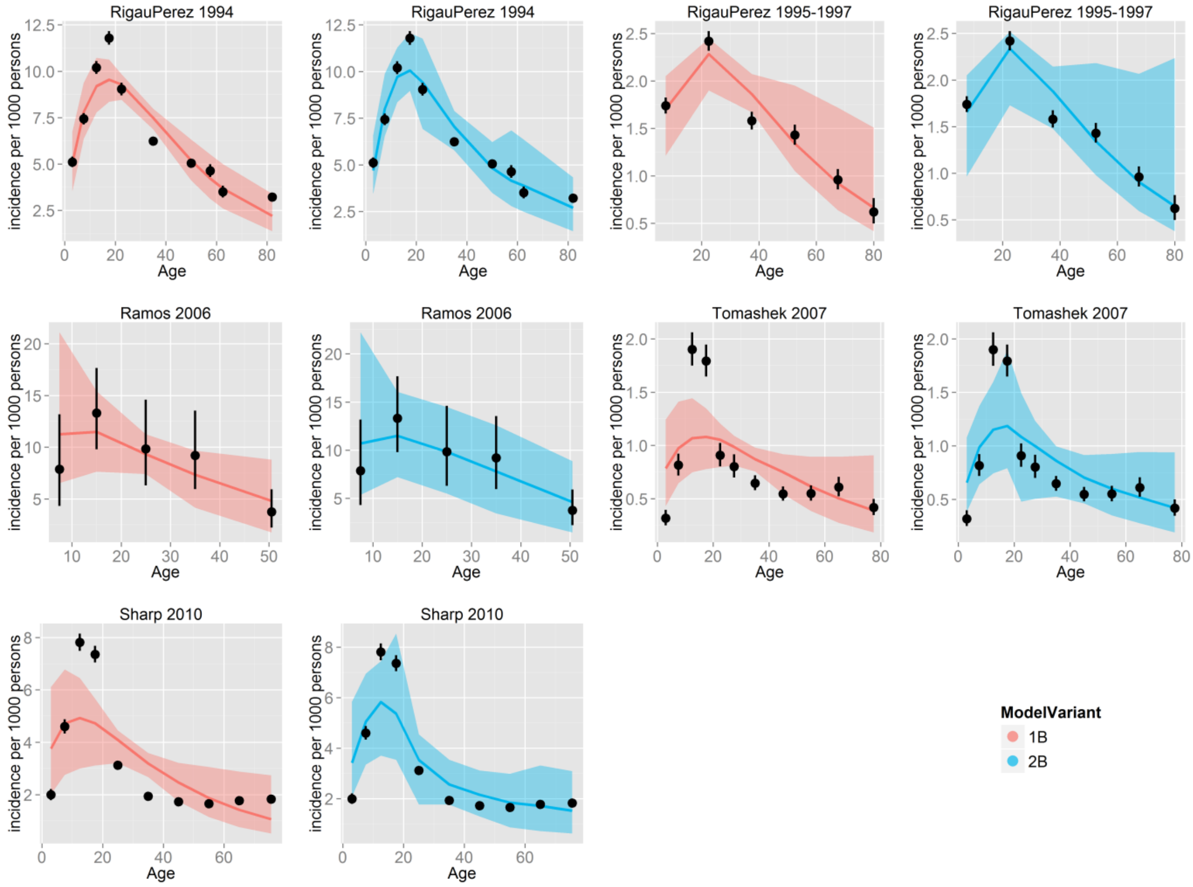

Fig S21: Model fits of model 1 (single reporting rate) and model 2 (age-dependent reporting rate) fitted to yearly incidence data from Singapore. 95% exact confidence interval around data point, posterior median (line) and 95% credible interval (shaded area) shown.

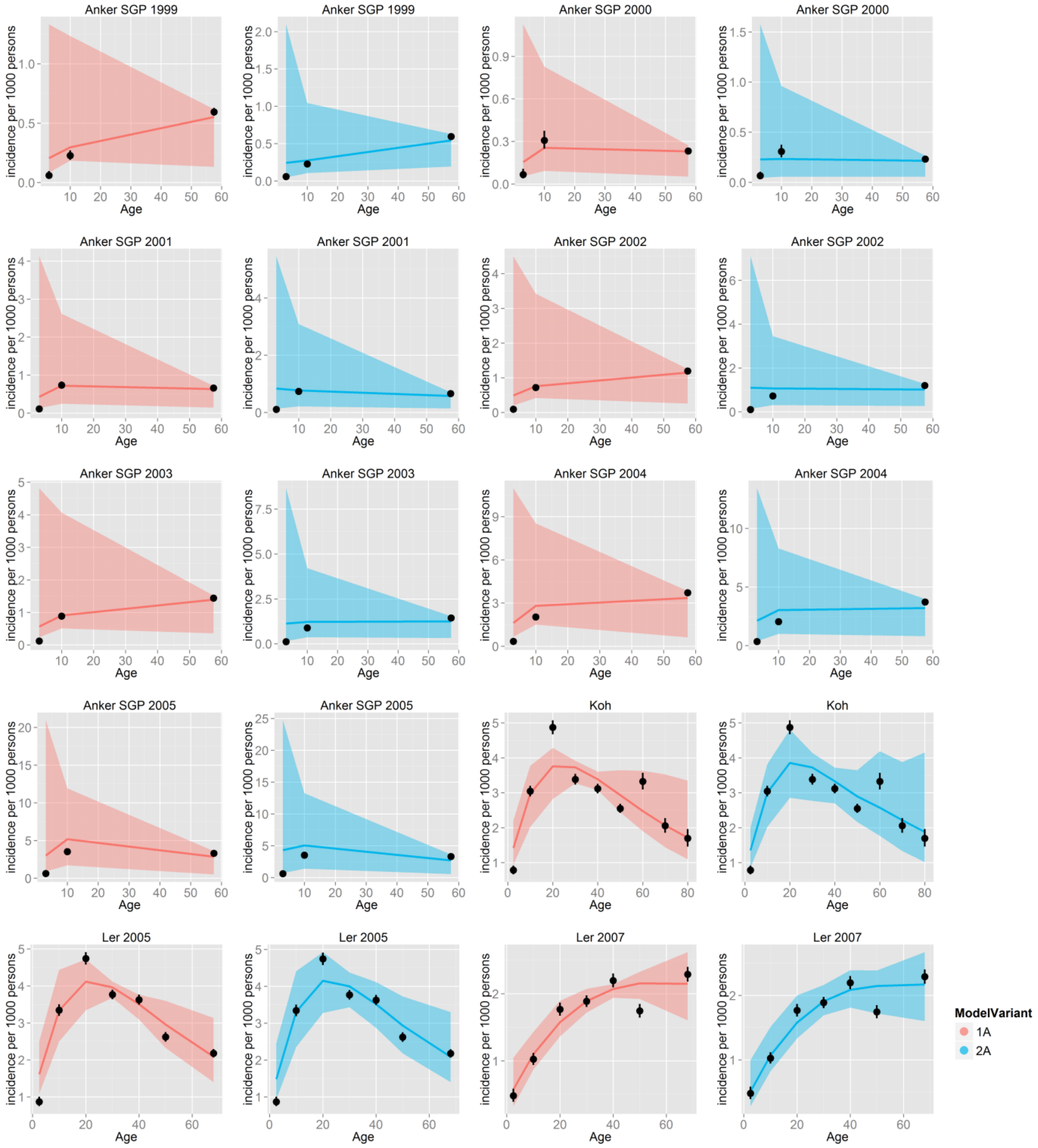

Fig S22: Model fits of model 1 (single reporting rate) and model 2 (age-dependent reporting rate) fitted to cumulative incidence data from Singapore. 95% exact confidence interval around data points, posterior median (line) and 95% credible interval (shaded area) shown.

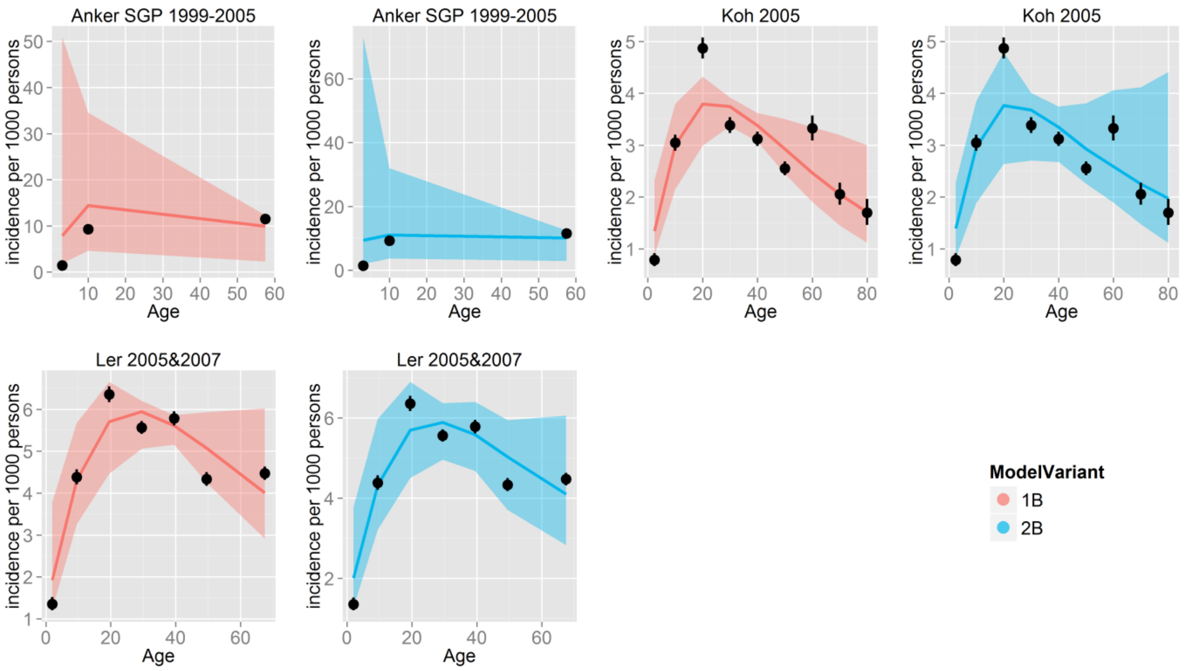

Fig S23: Model fits of model 1 (single reporting rate) and model 2 (age-dependent reporting rate) fitted to yearly incidence data from Sri Lanka. 95% exact confidence intervals around data points, posterior median (line) and 95% credible interval (shaded area) shown.

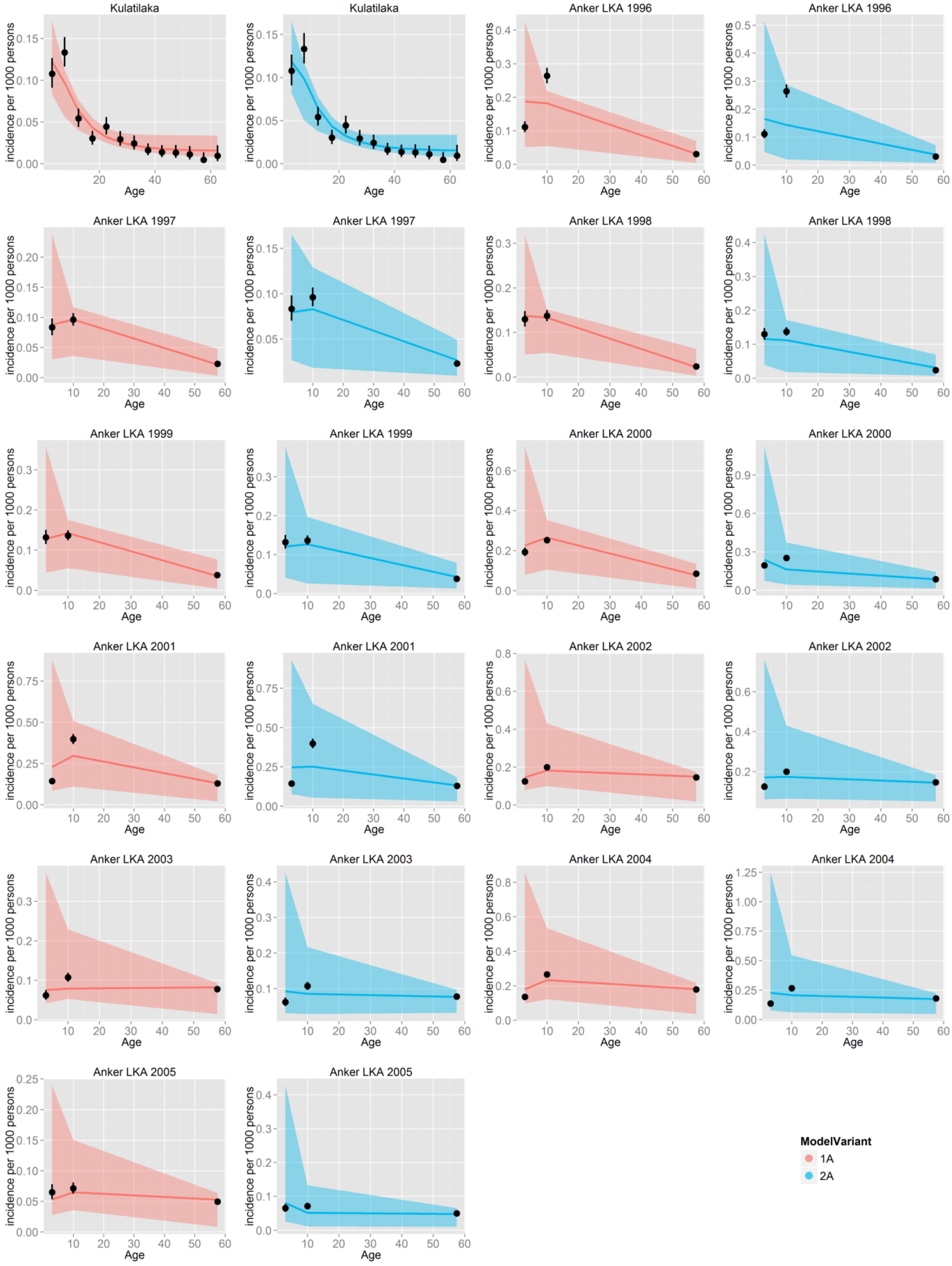

Fig S24: Model fits from model 1 (single reporting rate) and model 2 (age-dependent reporting rate) fitted to cumulative incidence data from Sri Lanka. 95% exact confidence interval around data points, posterior median (line) and 95% credible interval (shaded area) shown.

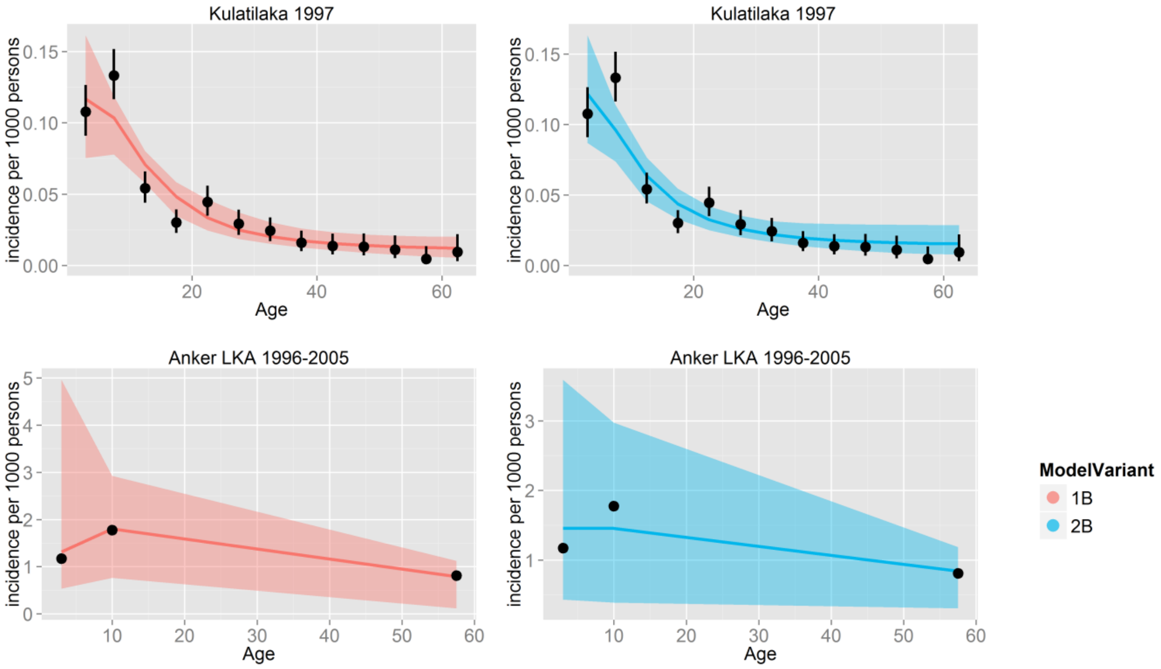

Fig S25: Model fits from model 1 (single reporting rate) and model 2 (age-dependent reporting rate) fitted to cumulative incidence data from Taiwan. 95% exact confidence intervals around data points, posterior median (line) and 95% credible intervals (shaded area) shown.

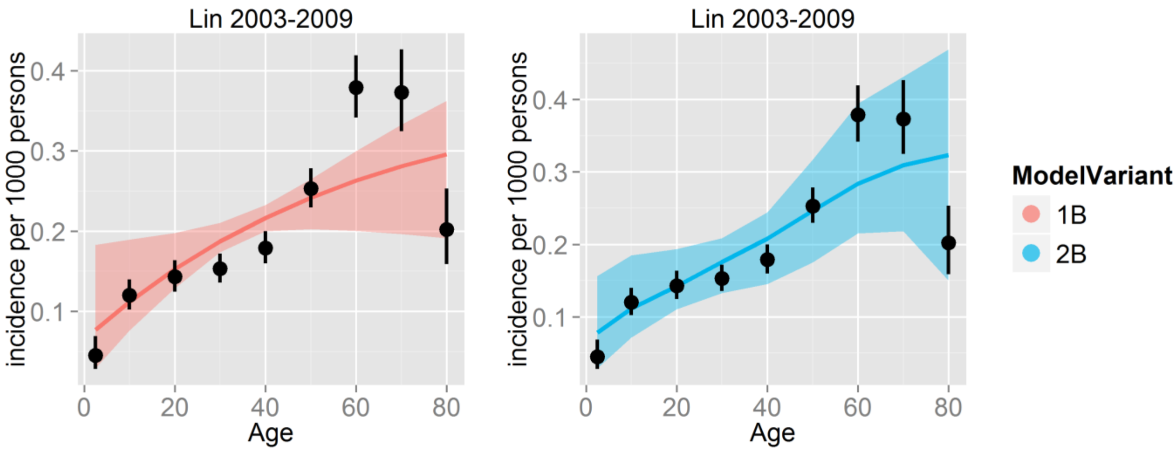

Fig S26: Model fits from model 1 (single reporting rate) and model 2 (age-dependent reporting rate) fitted to yearly incidence data from Thailand (Limkittikul *et al*). 95% exact confidence intervals around data points, posterior median (line) and 95% credible interval (shaded area) shown.

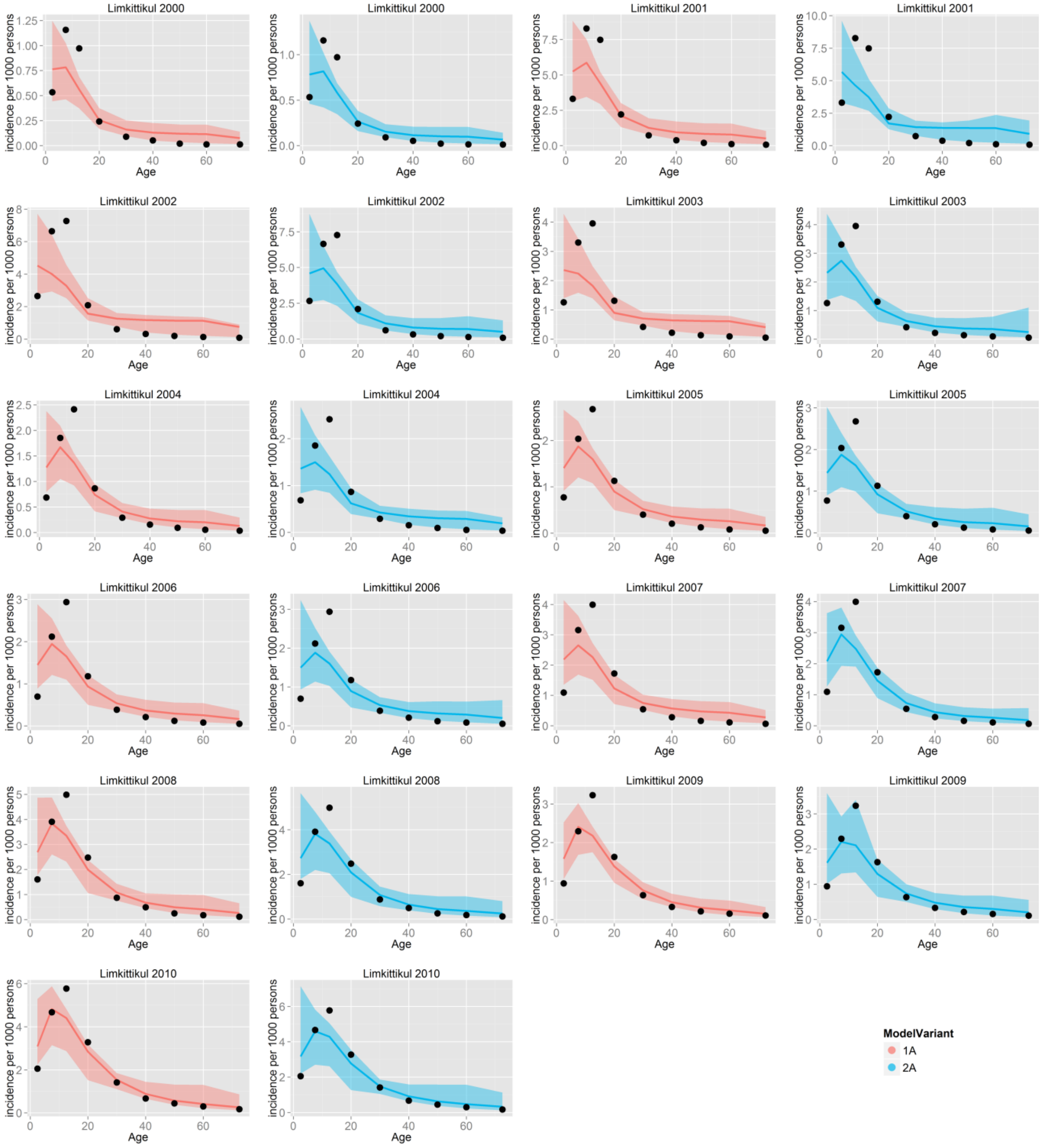

Fig S27: Model fits from model 1 (single reporting rate) and model 2 (age-dependent reporting rate) fitted to yearly incidence data from Thailand (continued). 95% exact confidence intervals around data points, posterior median (line) and 95% credible interval (shaded area) shown.

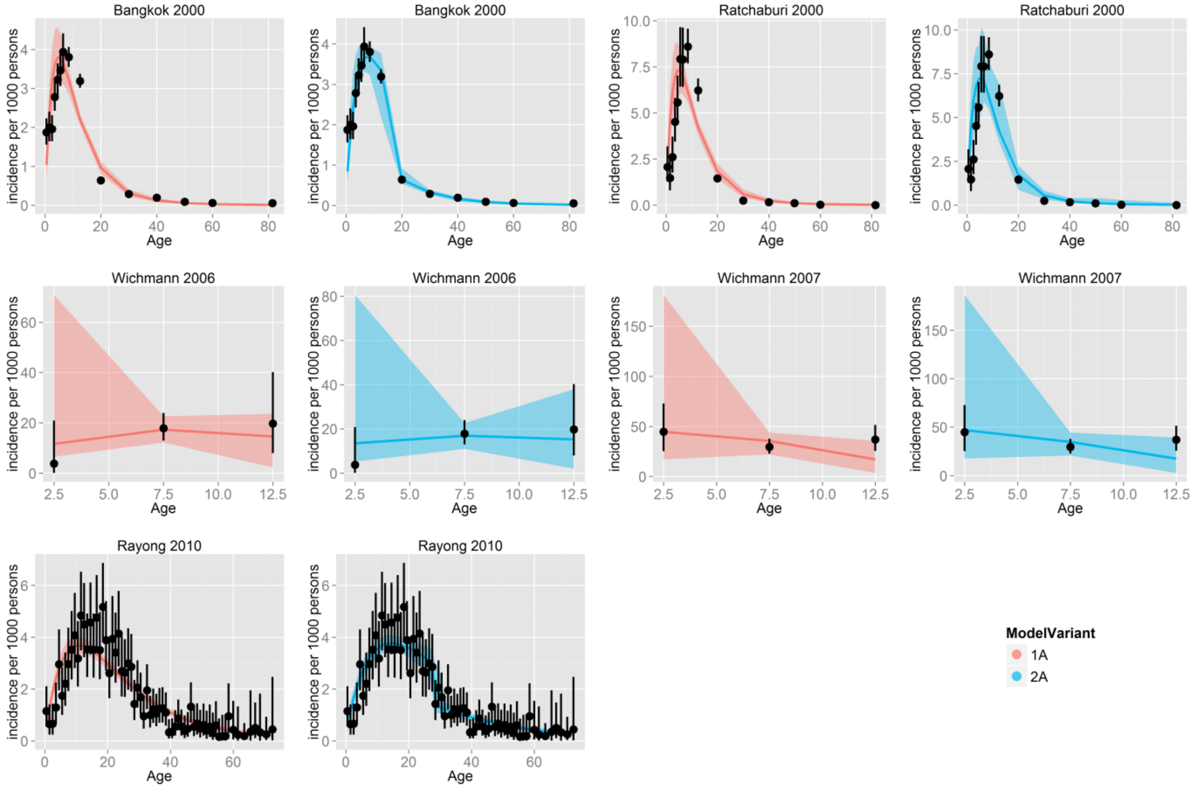

Fig S28: Model fits from model 1 (single reporting rate) and model 2 (age-dependent reporting rate) fitted to cumulative incidence data from Thailand. 95% exact confidence intervals around data points, posterior median (line) and 95% credible interval (shaded area) shown.

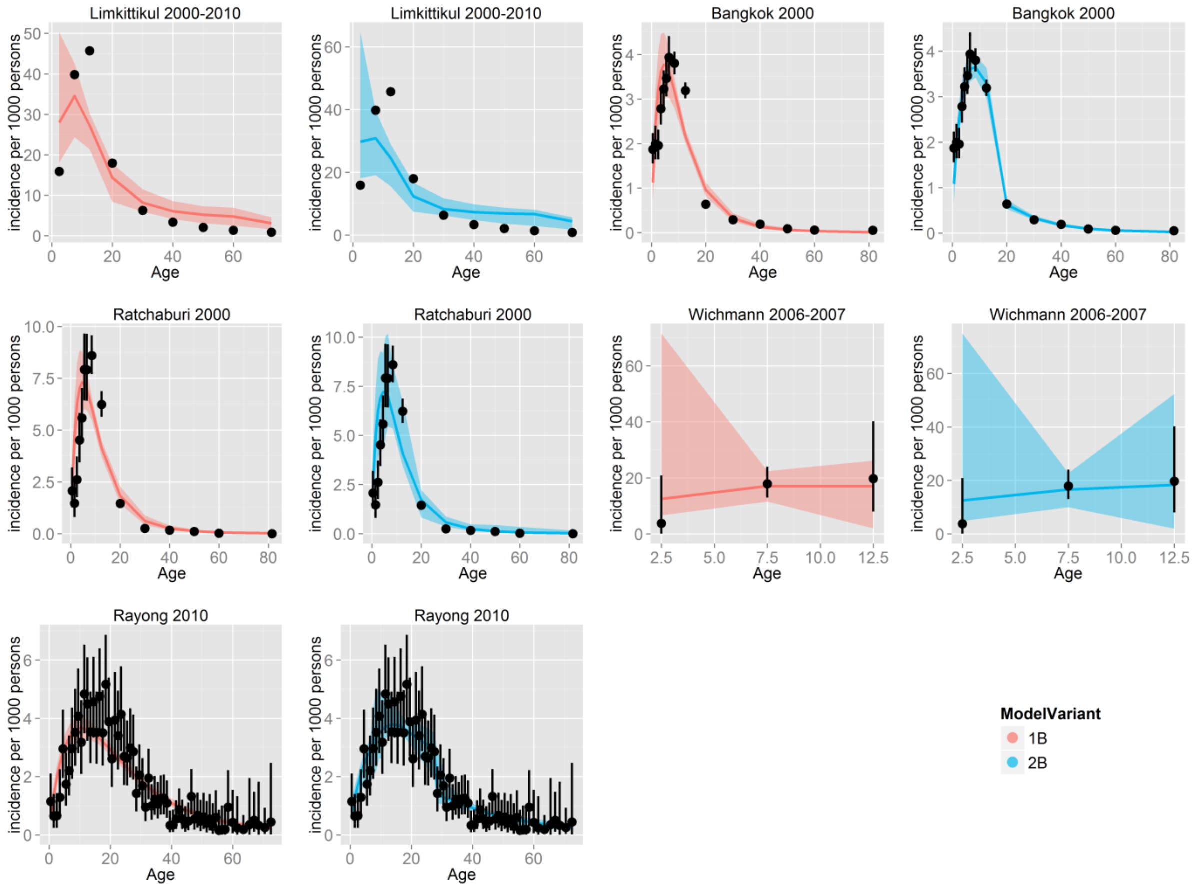

Fig S29: Model fits from model 1 (single reporting rate) and model 2 (age-dependent reporting rate) fitted to cumulative incidence data from Vietnam. 95% exact confidence intervals around data points, posterior median (line) and 95% credible interval (shaded area) shown.

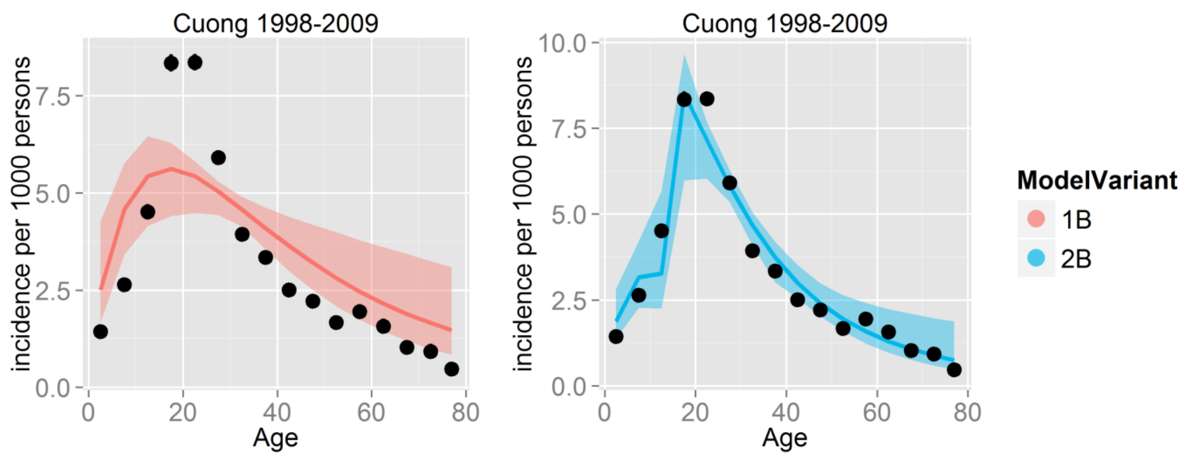

Fig S30: Model fits from model 1 (single reporting rate) and model 2 (age-dependent reporting rate) fitted to yearly incidence data from Yemen. 95% exact confidence intervals around data points, posterior median (line) and 95% credible interval (shaded area) shown.

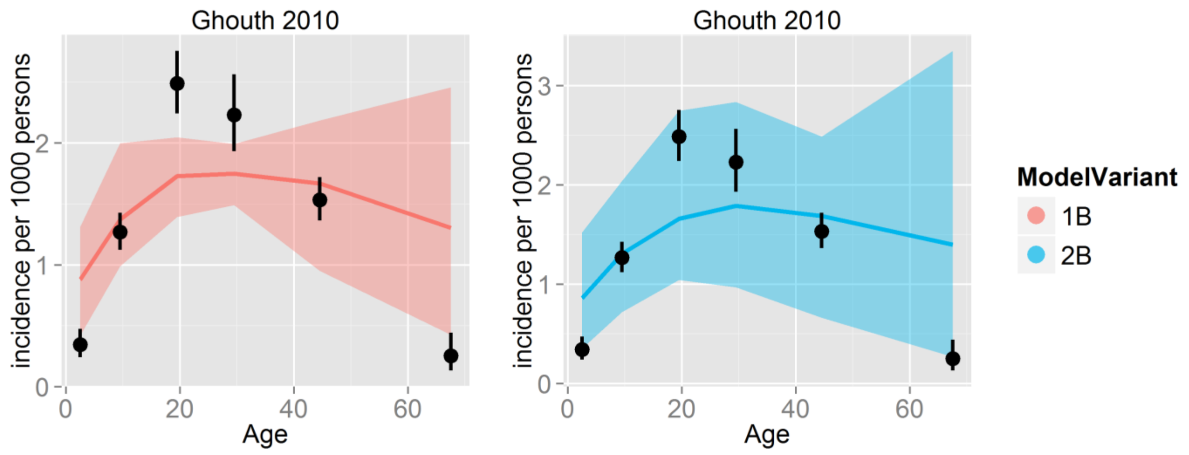

3 References

1. Halstead SB. Dengue in the Americas and Southeast Asia: do they differ? Rev Panam Salud Publica. 2007/03/08 ed. 2006;20: 407–415.

2. United Nations Population Division D of E and SA. World Population Prospects: The 2012 Revision. 2013.

3. Yu P, Shaw C a. An efficient algorithm for accurate computation of the Dirichlet-multinomial log-likelihood function. Bioinformatics. 2014;30: 1547–54. doi:10.1093/bioinformatics/btu079

4. Imai N, Dorigatti I, Cauchemez S, Ferguson NM. Estimating Dengue Transmission Intensity from Sero-Prevalence Surveys in Multiple Countries. PLoS Negl Trop Dis. 2015;9: e0003719. doi:10.1371/journal.pntd.0003719

5. Ferguson NM, Donnelly CA, Anderson RM. Transmission dynamics and epidemiology of dengue: insights from age-stratified sero-prevalence surveys. Philos Trans R Soc London Ser B-Biological Sci. 1999;354: 757–768. doi:10.1098/rstb.1999.0428

6. Fernando P, Vasconcelos C, Lima JWO, Raposo ML, Rodrigues SG, Travassos JFS, et al. Inquérito soro-epidemiológico na Ilha de São Luis durante epidemia de dengue no Maranhão Seroepidemiologic survey in São Luis Island , State of Maranhão , Brazil , during a dengue fever epidemics. 1999;32: 171–179.

7. Silva-Nunes M da, Souza V de. Risk factors for dengue virus infection in rural Amazonia: population-based cross-sectional surveys. Am J …. 2008;79: 485–494. Available: http://www.ajtmh.org/content/79/4/485.short

8. Lima VVLC de, Rangel O, Andrade VR, Silveira NYJ da, Oliveira SS de, Figueiredo LTM. Dengue: inquérito populacional para pesquisa de anticorpos e vigilância virológica no Município de Campinas, São Paulo, Brasil. Cad Saude Publica. 2007;23: 669–680. doi:10.1590/S0102-311X2007000300025

9. Shu PY, Chen LK, Chang SF, Yueh YY, Chow L, Chien LJ, et al. Potential application of nonstructural protein NS1 serotype-specific immunoglobulin G enzyme-linked immunosorbent assay in the seroepidemiologic study of dengue virus infection: Correlation of results with those of the plaque reduction neutralization test. J Clin Microbiol. 2002;40: 1840–1844. Available: http://ovidsp.ovid.com/ovidweb.cgi?T=JS&CSC=Y&NEWS=N&PAGE=fulltext&D=emed5&AN=2002168878

10. Ripley BD, Thompson M. Regression techniques for the detection of analytical bias. Analyst. 1987;112: 377. doi:10.1039/an9871200377

11. Therneau T. deming: Deming, Thiel-Sen and Passing-Bablock Regression [Internet]. 2014. Available: http://cran.r-project.org/package=deming

12. Penna G, Pinto LF, Soranz D, Glatt R. High incidence of diseases endemic to the Amazon region of Brazil, 2001-2006. Emerg Infect Dis. 2009;15: 626–32. doi:10.3201/eid1504.081329

13. Silva-Nunes M da, Malafronte R dos S, Luz B de A, Souza EA de, Martins LC, Rodrigues SG, et al. The Acre Project: the epidemiology of malaria and arthropod-borne virus infections in a rural Amazonian population. Cad Saude Publica. 2006;22: 1325–1334.

14. Cordeiro MTT, Silva AM, Brito C a a, Nascimento EJM, Magalhães MCF, Guimarães

GF, et al. Characterization of a dengue patient cohort in Recife, Brazil. Am J Trop Med Hyg. 2007;77: 1128–1134. Available: <Go to ISI>://WOS:000252123200026

15. Braga C, Luna CF, Martelli CM, de Souza W V, Cordeiro MT, Alexander N, et al. Seroprevalence and risk factors for dengue infection in socio-economically distinct areas of Recife, Brazil. Acta Trop. 2010;113: 234–240. doi:10.1016/j.actatropica.2009.10.021

16. Cardoso IM, Areias Cabidelle A de S, Borges P de CELP de C e L, Lang CF, Calenti FGFG, Nogueira L de O, et al. Dengue: clinical forms and risk groups in a high incidence city in the Southeastern region of Brazil. Rev Soc Bras Med Trop. 2011;44: 430–435. doi:10.1590/S0037-86822011005000044

17. de Lima VLC, Figueiredo LTM, Correa HR, Leite OF, Rangel O, Vido AA, et al. Dengue fever: a post-epidemic sero-epidemiological survey in an urban setting at a northwestern county of S. Paulo State - Brazil. Rev Saude Publica. 1999;33: 566–574.

18. Anker M, Arima Y. Male–female differences in the number of reported incident dengue fever cases in six Asian countries. West Pacific Surveill Response …. 2011;2: 1–7. doi:10.5365/wpsar.2011.2.1.002

19. Vallee J, Dubot-Peres A, Ounaphom P, Sayavong C, Bryant JE, Gonzalez J-P, et al. Spatial distribution and risk factors of dengue and Japanese encephalitis virus infection in urban settings: The case of Vientiane, Lao PDR. Trop Med Int Heal. 2009;14: 1134–1142. doi:http://dx.doi.org/10.1111/j.1365-3156.2009.02319.x

20. Hammond SN, Balmaseda A, Perez L, Tellez Y, Saborio SI, Mercado JC, et al. Differences in dengue severity in infants, children, and adults in a 3-year hospital-based study in Nicaragua. Am J Trop Med Hyg. 2005;73: 1063–1070.

21. Balmaseda A, Standish K, Mercado JC, Matute JC, Tellez Y, Saborio S, et al. Trends in patterns of dengue transmission over 4 years in a pediatric cohort study in Nicaragua. J Infect Dis. 2010;201: 5–14. doi:10.1086/648592

22. Yew YW, Ye T, Ang LW, Ng LC, Yap G, James L, et al. Seroepidemiology of dengue virus infection among adults in Singapore. Ann Acad Med Singapore. 2009;38: 667–675. Available: <Go to ISI>://WOS:000269938400002

23. Ler TS, Ang LW, Yap GSL, Ng LC, Tai JC, Jamesb L, et al. WPRO | Epidemiological characteristics of the 2005 and 2007 dengue epidemics in Singapore – similarities and distinctions. West Pacific Surveill Response J. WPRO | WHO Western Pacific Region; 2011;2: 24–29. Available: http://www.wpro.who.int/wpsar/volumes/02/2/2011_OR_Ler_etal/en/

24. Yap G, Li C, Mutalib A, Lai Y-L, Ng L-C. High rates of inapparent dengue in older adults in Singapore. Am J Trop Med Hyg. 2013;88: 1065–9. doi:10.4269/ajtmh.12-0150

25. Lin C-H, Schiøler KL, Jepsen MR, Ho C-K, Li S-H, Konradsen F. Dengue outbreaks in high-income area, Kaohsiung City, Taiwan, 2003-2009. Emerg Infect Dis. 2012;18: 1603–11. doi:10.3201/eid1810.111929

26. Bureau of Epidemiology, Ministry of Public Health of Thailand. Bureau of Epidemiology, Ministry of Public Health of Thailand (YEAR) Annual epidemiological surveillance report. Nonthaburi, Thailand.

27. Perret C, Chanthavanich P, Pengsaa K, Limkittikul K, Hutajaroen P, Bunn JE, et al.

Dengue infection during pregnancy and transplacental antibody transfer in Thai mothers. J Infect. 2005;51: 287–293. doi:10.1016/j.jinf.2004.10.003

28. Tuntaprasart W, Barbazan P, Nitatpattana N, Rongsriyam Y, Yoksan S, Gonzalez JP. Seroepidemiological survey among schoolchildren during the 2000-2001 dengue outbreak of Ratchaburi Province, Thailand. Southeast Asian J Trop Med Public Health. 2003;34: 564–568. Available: <Go to ISI>://BCI:BCI200400103433

29. Rodriguez-Barraquer I, Cordeiro MT, Braga C, de Souza W V, Marques ET, Cummings DAT. From re-emergence to hyperendemicity: the natural history of the dengue epidemic in Brazil. PLoS Negl Trop Dis. 2011;5: e935. doi:10.1371/journal.pntd.0000935

30. Cuong HQ, Hien NT, Duong TN, Phong T V, Cam NN, Farrar J, et al. Quantifying the Emergence of Dengue in Hanoi, Vietnam: 1998-2009. PLoS Negl Trop Dis. 2011;5: e1322–e1322. doi:10.1371/journal.pntd.0001322

31. Bartley LM, Carabin H, Vinh Chau N, Ho V, Luxemburger C, Hien TT, et al. Assessment of the factors associated with flavivirus seroprevalence in a population in Southern Vietnam. Epidemiol Infect. 2002;128: 213–220. doi:10.1017/s0950268801006495

32. Cordeiro MTT, Schatzmayr HGG, Nogueira RMR, De Oliveira VF, De Melo WT, de Carvalho EF. Dengue and dengue hemorrhagic fever in the State of Pernambuco, 1995-2006. Rev Soc Bras Med Trop. 2007;40: 605–611. doi:10.1590/s0037-86822007000600001

33. Vong S, Khieu V, Glass O, Ly S, Duong V, Huy R, et al. Dengue incidence in urban and rural Cambodia: results from population-based active fever surveillance, 2006-2008. PLoS Negl Trop Dis. 2010;4: e903. doi:10.1371/journal.pntd.0000903

34. Wichmann O, Yoon I-K, Vong S, Limkittikul K, Gibbons R V, Mammen MP, et al. Dengue in Thailand and Cambodia: an assessment of the degree of underrecognized disease burden based on reported cases. PLoS Negl Trop Dis. 2011;5: e996. doi:10.1371/journal.pntd.0000996

35. Rigau-Pérez JG, Ayala-López A, Vorndam AV, Clark GG, Rigau-Perez JG, Ayala-Lopez A, et al. Dengue activity in Puerto Rico during an interepidemic period (1995-1997). Am J Trop Med Hyg. 2001;64: 75–83. Available: <Go to ISI>://BCI:BCI200200235758

36. Ramos MM, Argueello DF, Luxemburger C, Quinones L, Munoz JL, Beatty M, et al. Epidemiological and clinical observations on patients with dengue in Puerto Rico: Results from the first year of enhanced surveillance - June 2005-May 2006. Am J Trop Med Hyg. 2008;79: 123–127. Available: <Go to ISI>://WOS:000257407700022

37. Sharp TM, Hunsperger E, Santiago GA, Munoz-Jordan JL, Santiago LM, Rivera A, et al. Virus-specific differences in rates of disease during the 2010 Dengue epidemic in Puerto Rico. PLoS Negl Trop Dis. 2013/04/18 ed. 2013;7: e2159. doi:10.1371/journal.pntd.0002159

38. Tomashek KM, Rivera A, Munoz-Jordan JL, Hunsperger E, Santiago L, Padro O, et al. Description of a Large Island-Wide Outbreak of Dengue in Puerto Rico, 2007. Am J Trop Med Hyg. 2009;81: 467–474.

39. Koh BKW, Lee CN, Kita Y, Choon ST, Li WA, Kit YW, et al. The 2005 dengue epidemic in Singapore: Epidemiology, prevention and control. Ann Acad Med

Singapore. 2008;37: 538–545. Available: http://ovidsp.ovid.com/ovidweb.cgi?T=JS&CSC=Y&NEWS=N&PAGE=fulltext&D=emed8&AN=2008387646

40. Kulatilaka TA, Jayakuru WS. Control of dengue/dengue haemorrhagic fever in Sri Lanka. Dengue Bull. 1998;22: 53–59. Available: <Go to ISI>://CABI:20000508522

41. Limkittikul K, Brett J, L'Azou M. Epidemiological Trends of Dengue Disease in Thailand (2000–2011): A Systematic Literature Review. PLoS Negl Trop Dis. 2014;8: e3241. doi:10.1371/journal.pntd.0003241

42. Ghouth AS, Amarasinghe A, Letson GW, Bin Ghouth AS. Dengue outbreak in Hadramout, Yemen, 2010: an epidemiological perspective. Am J Trop Med Hyg. 2012;86: 1072–1076. doi:10.4269/ajtmh.2012.11-0723

43. Khampapongpane B, Lewis HC, Ketmayoon P, Phonekeo D, Somoulay V, Khamsing A, et al. National dengue surveillance in the Lao People's Democratic Republic, 2006–2012: epidemiological and laboratory findings. West Pacific Surveill Response J. 2014;5: 2006–2012. doi:10.5365/wpsar.2014.5.1.001

44. Prasith N, Keosavanh O, Phengxay M. Assessment of gender distribution in dengue surveillance data, the Lao People's Democratic Republic. West Pacific Surveill Response J. 2011;4: 1–9. doi:10.5365/wpsar.2012.3.4.020

45. Luo L, Liang H, Hu Y, Liu W, Wang Y, Jing Q, et al. Epidemiological, virological, and entomological characteristics of dengue from 1978 to 2009 in Guangzhou, China. J Vector Ecol. 2012;37: 230–240. doi:10.1111/j.1948-7134.2012.00221.x

46. Guo R, Lin J, Li L-H, Ke C-W, He J, Zhong H, et al. The Prevalence and Endemic Nature of Dengue Infections in Guangdong, South China: An Epidemiological, Serological, and Etiological Study from 2005-2011. PLoS One. 2014;9: e85596. doi:10.1371/journal.pone.0085596
